# Copas‐Heckman‐Type Sensitivity Analysis for Publication Bias in Rare‐Event Meta‐Analysis Under Generalized Linear Mixed Models

**DOI:** 10.1002/sim.70595

**Published:** 2026-05-12

**Authors:** Yi Zhou, Taojun Hu, Yuji Sakamoto, Ao Huang, Xiao‐Hua Zhou, Satoshi Hattori

**Affiliations:** ^1^ Division of Mathematics and Informatics, Graduate School of Human Development and Environment Kobe University Kobe Japan; ^2^ Beijing International Center for Mathematical Research Peking University Beijing China; ^3^ Department of Biomedical Statistics Graduate School of Medicine, The University of Osaka Osaka Japan; ^4^ School of Medicine, Chonqing University Chongqing China; ^5^ Department of Biostatistics Peking University Beijing China; ^6^ Graduate School of Information Sciences, Tohoku University Sendai Japan; ^7^ Integrated Frontier Research for Medical Science Division, Institute for Open and Transdisciplinary Research Initiatives The University of Osaka Osaka Japan

**Keywords:** binomial distribution, continuity correction, hypergeometric distribution, random‐effects meta‐analysis, rare events, selection function

## Abstract

In systematic reviews and meta‐analyses, publication bias (PB) is one of the serious concerns and mainly induced by selective publication of academic literatures. Although many methods have been proposed to address PB, almost all of them are based on the normal‐normal (NN) random‐effects model, assuming that data are normally distributed at both the within‐study and between‐study levels. For rare‐event meta‐analysis where data contain rare occurrences of events, the standard NN random‐effects model may perform poorly. Instead, some generalized linear mixed models (GLMMs) which employ the exact distribution for the number of events at the within‐study level increase the estimation accuracy and have been widely used in practice. However, limited methods can be applied to address PB in the GLMMs. To address this limitation, we propose a framework of sensitivity analysis for evaluating the impact of PB in the contrast‐based GLMMs. The proposed framework is developed based on the famous Copas‐Heckman‐type sensitivity analysis methods by relating study‐specific true effect sizes with the latent Gaussian variable on the study sample sizes for adjusting PB. The proposed methods completely avoid the need for continuity corrections and can be easily implemented using standard software with low computational cost. Simulation studies are conducted to assess the performance of the proposed methods in adjusting PB and compare the results with related existing methods. Several real‐world examples are also analyzed to show the broad applicability of our proposal in evaluating the potential impact of PB in meta‐analysis of odds ratios and proportions with rare‐event outcomes.

## 1 Introduction

Systematic reviews and meta‐analyses are widely used in a variety of areas to summarize the overall effect of an intervention or the difference between two groups. Usually, the overall effect is estimated by a two‐level random‐effects model. At the within‐study level, it is assumed that the observed outcomes of each study follow a normal distribution with a study‐specific true mean and a known variance of outcomes. At the between‐study level, the true means across studies are distributed as a normal distribution with an overall mean and a between‐study variance, which are the parameters of interest. This method is considered to be the standard approach for meta‐analysis and is also known as the inverse‐variance method [1], the linear mixed‐effects model, or the normal‐normal (NN) random‐effects model (hereinafter referred to as the NN model). When the outcomes are binary, a common practice is to transform the outcomes into continuous values and apply a normal approximation in order to use the NN model. For example, in a meta‐analysis comparing two groups, it is often of interest whether an event (e.g., an adverse event) is more or less likely to occur in the treatment group. The treatment effect is commonly quantified by the odds ratio (OR) or the log‐transformed OR (lnOR), which compares the odds of the event between two groups.

A major issue with these measures arises when the observed number of events is close to or contains zero, resulting in the estimate of OR and its standard error (SE) being either zero or undefined. To deal with this, continuity correction methods [2] are commonly applied. However, these methods are often criticized for lacking a sound theoretical rationale, and no single method is consistently recommended [3]. Despite the limitations of the continuity correction methods, the small number of events, also referred to as rare events, can lead to problematic estimation when using the NN model. In the example above, the lnORs are typically modeled using the NN model. However, in situations of rare events or small sample sizes, the normal approximation used at the within‐study level becomes questionable. Furthermore, the NN model assumes independence between lnOR estimates and their SEs, which may not hold in the presence of rare events and also introduces bias [4, 5]. Alternatively, generalized linear mixed models (GLMMs) offer a more accurate estimation by modeling the number of events using exact likelihoods for discrete random variables [4, 5, 6]. The advantages and performance of various GLMMs have been extensively evaluated through simulation studies [4, 5, 6, 7, 8, 9, 10, 11]. These GLMMs can be classified into the contrast‐based models, which directly estimates the overall lnORs given fixed numbers of total events (e.g., model 1 and 7 in Jackson et al. [9]), or the arm‐based models, which focuses on modeling the log odds of each arm to estimate the overall lnORs (e.g., model 2–6 in Jackson et al. [9]) [12]. While both kinds of GLMMs are useful, the contrast‐based models estimate the overall relative measure (e.g., the overall lnOR) directly and requires less parameters without explicitly modeling the baseline measure. In most meta‐analysis, the overall lnORs are of more interest than the log odds; thus, we focus on the contrast‐based models in this paper. In the contrast‐based GLMMs, the widely used ones use hypergeometric or binomial distribution at the within‐study level and a normal distribution at the between‐study level [4, 5, 6]; in this paper, these models are in general referred to as the hypergeometric‐normal (HN) random‐effects model (hereinafter referred to as the HN model) and the binomial‐normal (BN) random‐effects model (hereinafter referred to as the BN model), respectively.

In systematic reviews, a common and unavoidable issue is publication bias (PB)–a phenomenon in which studies with significant results are more likely to be published–potentially leading to over‐optimistic conclusions in meta‐analyses [13]. In recent decades, many sophisticated methods incorporating selection functions into estimation frameworks have been proposed to quantitatively assess the potential impact of PB [13, 14]. Famous ones include the sensitivity analysis methods proposed by Copas and his colleagues, such as the Copas‐Heckman‐type sensitivity analysis methods [15, 16, 17], which extended the Heckman model [18, 19] in the field of econometrics into meta‐analysis models. Others include the t‐statistic‐based selection model [20] and the non‐parametric worst‐case bounds [21]. Among these, the Copas‐Heckman‐type sensitivity analysis methods [15, 16, 17] has been extensively developed [22, 23, 24, 25] and further extended to address PB in multivariate meta‐analyses [26, 27, 28, 29]. Almost all of these methods were developed based on the NN model or its multivariate versions. An exception is the method of Hattori and Zhou [26], which extended the Copas‐Heckman‐type sensitivity analysis methods into the meta‐analysis of diagnostic studies with bivariate exact within‐study likelihood.

Although numerous exact likelihood‐based models have been proposed for summarizing overall effects for rare‐event meta‐analyses, these models do not account for PB. To the best of our knowledge, limited research has been studied to address PB in models other than the NN model; the only known proposal is that of Hu et al. [30] This method extended the t‐statistic‐based selection model [20] into the HN and BN models; it assumes selective publication to be related to the t‐statistics (equivalently, p values) of studies and successfully adjusts PB using a sensitivity analysis approach. However, one of the limitations of the existing proposal [30] is its high computational burden. To facilitate estimation, its algorithm employs an approximation technique, which may also result in some degree of estimation error [30]. In addition, since the t‐statistics are required for addressing PB in the existing method [30], the continuity correction is still required in constructing the selection function; thus, the estimations might be influenced by different choices of continuity correction. On the other hand, in rare‐event studies, the estimated lnORs and their SEs are usually related [4], which may distort the derivation of t‐statistics and the corresponding p values. In the widely used funnel plots, square root of sample sizes is used as one of the factors to detect PB [31, 32]. In addition, Copas [15] used a Gaussian latent variable on the square root of study sample sizes to model the selective publication; however, this method is proposed partially based on the NN model. In this paper, we consider adopting the Copas‐Heckman‐type selection function based on the study sample sizes, as alternative to the method of Hu et al. [30], to develop the sensitivity analysis framework for addressing PB in rare‐event meta‐analyses. The Copas‐Heckman type selection function is advantageous for the NN model in a technical point of view; the conditional likelihood given published can be derived in a straightforward matter with normal properties of the NN model. For the GLMM, it is not straightforward to realize a Copas‐Heckman‐type sensitivity analysis. By introducing an alternative formulation that models the dependence of the outcome on a Gaussian latent variable responsible for the selective publication process, we introduce a simple method to implement sensitivity analysis with the Copas‐Heckman‐type selection function for the GLMM. The proposed method completely avoids the need for continuity correction during estimation and is substantially simpler in terms of computational implementation. While PB is often attributed to statistical significance, it remains important to consider other potential sources, such as the sizes of studies, as part of sensitivity analysis to derive robust conclusions in meta‐analysis. Our proposal offers a practical alternative for addressing PB in the context of rare‐event meta‐analyses.

The rest of this paper is organized as follows. In Section 2, we review the standard NN random‐effects meta‐analysis model for summarizing the binary outcomes and present two Copas‐Heckman‐type sensitivity analysis methods. In Section 3, we introduce the commonly used GLMMs for rare‐event meta‐analyses. In Section 4, we propose the framework of novel sensitivity analysis methods for PB in different GLMMs. The performances of the proposed methods in adjusting PB are studied using stimulation experiments in Section 5. In Section 6 we demonstrate the practical applicability of the proposed methods through several real‐world meta‐analyses. Finally, we conclude with a discussion in Section 7. Some results are presented in the Supporting Information.

## 2 COPAS‐Heckman‐Type Sensitivity Analysis for Publication Bias

### 2.1 NN Random‐Effects Model

In this section, we focus on meta‐analysis under the assumption that PB is absent. Suppose that we are interested in a meta‐analysis containing N studies with binary outcomes, where the studies are randomly published from the population of all relevant studies. Each study contains treatment and control groups, and the numbers of events and subjects are accessible from clinical literature. Let $y_{i0}$ and $n_{i0}$ be the number of events and subjects in the control group, respectively, and $y_{i1}$ and $n_{i1}$ be those in the treatment group. These data in study i can be formulated by the contingency matrix in Table 1.

**TABLE 1:** Example of the contingency table for data of study i.

	Event	Non‐event	Total
Treatment	$y_{i1}$	$n_{i1}-y_{i1}$	$n_{i1}$
Control	$y_{i0}$	$n_{i0}-y_{i0}$	$n_{i0}$
Total	$y_{i}$	$n_{i}-y_{i}$	$n_{i}$

With two groups of data, the overall OR or its log‐transformation (i.e., lnOR) is widely used as the effect measure of interest in clinical and epidemiological research. The common practice of meta‐analysis is based on the NN model [1]. For the *i*th study in Table 1, one can estimate the lnOR, denoted by ${\hat{\theta }}_{i}$, and its SE, denoted by $s_{i}$, with

$${\hat{\theta }}_{i}=\log\frac{y_{i1}/\left(n_{i1}-y_{i1}\right)}{y_{i0}/\left(n_{i0}-y_{i0}\right)}\;\text{and}\;s_{i}=\sqrt{\frac{1}{y_{i1}}+\frac{1}{n_{i1}-y_{i1}}+\frac{1}{y_{i0}}+\frac{1}{n_{i0}-y_{i0}}},$$

where the lnOR measures how the odds of the event of interest differ between the treatment and control groups. When zero entries appear in Table 1, the estimates of ${\hat{\theta }}_{i}$ and $s_{i}$ become undefined. In such cases, the continuity correction is usually conducted. There are various approaches to implementing continuity corrections, which involve adding different constants to some or all studies; however, these choices have some influence on the estimate of the overall lnOR. Zabriskie et al. [3] extensively compared various approaches of continuity correction by a simulation study and found that no one method could be recommended; Sweeting et al. [2] suggested a sensitivity analysis across a number of correction constants. A common approach is to add 0.5 to both the numbers of events and non‐events prior to estimating the lnOR and SE [2].

With the estimates of ${\hat{\theta }}_{i}$ and $s_{i}$, the NN model can be applied [1]. At the within‐study level, it is assumed that the estimated lnOR approximately follows a normal distribution:

(1) $${\hat{\theta }}_{i}|\theta_{i}∼N\left(\theta_{i},s_{i}^{2}\right),$$

where $\theta_{i}$ is the true lnOR for each study; following the convention in meta‐analysis field, $s_{i}$ is regarded as known and estimated. The normal approximation in the within‐study model (1) holds when the sizes of studies are sufficiently large and the event probabilities are not extremely close to 0 or 1. At the between‐study level, the true lnORs across the studies are assumed to have the following normal distribution:

(2) $$\theta_{i}∼N\left(\theta ,\tau^{2}\right),$$

where θ is the overall lnOR of interest, and $\tau^{2}$ is the between‐study variance. Marginally, by combining models (1) and (2), ${\hat{\theta }}_{i}$ has the following normal distribution:

(3) $${\hat{\theta }}_{i}∼N\left(\theta ,s_{i}^{2}+\tau^{2}\right).$$

Allowing for the existence of between‐study heterogeneity, the parameters (θ,τ) can be estimated by the maximum likelihood (ML) method based on the following likelihood:

(4) $$\begin{array}{cc} L\left(\theta ,\tau \right) & =\prod_{i=1}^{N}\int_{-\infty }^{\infty }L_{i}\left(\theta_{i}\right)\frac{1}{\tau }\phi \left(\frac{\theta_{i}-\theta }{\tau }\right)\;d\theta_{i}\;\text{with}\;L_{i}\left(\theta_{i}\right)\\ & =\frac{1}{\sqrt{2\pi }s_{i}}\exp\left(-\frac{{\left({\hat{\theta }}_{i}-\theta_{i}\right)}^{2}}{2s_{i}^{2}}\right), \end{array}$$

where ϕ(.) denotes the probability density function of the standard normal distribution.

Sometimes, the occurrence of an event in only one group is of primary interest. In such case, the parameter of interest is the event proportion, denoted by π. To take an example, π can be the overall probability of an adverse event occurring in the treatment group. The standard approach is to model the log odds of the event, or equivalently, the logit‐transformed proportion, denoted by $\theta_{i}=\text{logit}\left(\pi_{i}\right)$, where $\pi_{i}$ represents the study‐specific true occurrence probability of the event, and logit(x)=log(x/(1−x)) is the logit function. The log odds can be estimated as ${\hat{\theta }}_{i}$ based on data of the treatment group only in Table 1. With the observed number of events ($y_{i1}$) and total number of subjects in the treatment group ($n_{i1}$), the log odds and their SE are estimated as

$${\hat{\theta }}_{i}=\log\frac{y_{i1}}{n_{i1}-y_{i1}}\;\text{and}\;s_{i}=\sqrt{\frac{1}{y_{i1}}+\frac{1}{n_{i1}-y_{i1}}}.$$

Similarly, the continuity correction is also required when there are zero events, although it may cause some variants in the results. Under the large sample conditions, the estimated log odds can also be assumed to follow the normal distribution of model (3), allowing the overall effect parameter θ to be estimated using the ML method. Finally, the parameter of interest, π, is obtained by transforming the estimated θ.

### 2.2 Copas‐Heckman‐Type Sensitivity Analyses

PB has always been one of the major issues in meta‐analysis. In the presence of PB, the published N studies are not likely to be random sample from the population. Copas and Shi [16] proposed one sensitivity analysis method for addressing PB in the NN model (3) by developing the Heckman‐type selection function [18, 19].

In this method, outcomes ${\hat{\theta }}_{i}$ are re‐expressed as follows:

$${\hat{\theta }}_{i}=\theta +{\left(\sigma_{i}^{2}+\tau^{2}\right)}^{1/2}\epsilon_{i},$$

where $\epsilon_{i}$ is the standard normal residual, and $\sigma_{i}^{2}=\mathrm{Var}\left(y_{i}|\theta_{i}\right)$ is the within‐study sampling variance. Copas and Shi [16] conjectured that selective publication process is related to the SEs of studies; thus, a latent Gaussian variable $Z_{i}$ with the selection equation concerning the SE of each study was proposed, that is,

$$Z_{i}=\beta_{0}+\frac{\beta_{1}}{s_{i}}+\delta_{i},$$

where $\beta_{0}$ and $\beta_{1}$ are constants, and $\delta_{i}$ is the residual following the standard normal distribution, N(0,1). Study i is published if and only if $Z_{i}>0$. The correlation between $\epsilon_{i}$ and $\delta_{i}$, denoted by ρ, was introduced to link selective publication process with the outcomes. Thus, the joint distribution of $\epsilon_{i}$ and $\delta_{i}$ can be written as follows:

$$\left(\begin{array}{c} \epsilon_{i}\\ \delta_{i} \end{array}\right)∼N\left(\left(\begin{array}{c} 0\\ 0 \end{array}\right),\left[\begin{array}{cc} 1 & \rho \\ \rho & 1 \end{array}\right]\right).$$

When ρ≠0, the publication of a study is related to its outcomes; in contrast, ρ=0 indicates that the study outcomes do not influence its publication. The probability of $Z_{i}>0$ can be re‐expressed by the probit model:

$$P\left(Z_{i}>0|s_{i}\right)=\Phi \left(\beta_{0}+\frac{\beta_{1}}{s_{i}}\right),$$

where Φ(.) is the standard normal cumulative distribution function. The probability of $Z_{i}>0$ represents selective publication of one study, indicating that studies with smaller SEs, which are usually large studies, are more likely to be published.

Under selective publication of studies, the log‐likelihood conditional on the published studies was derived as follows:

(5) $$\begin{array}{cc} & \ell \left(\theta ,\tau ,\rho ,\beta_{0},\beta_{1}\right)=\sum_{i=1}^{N}\log p\left({\hat{\theta }}_{i}|z_{i}>0,s_{i}\right)\\ & \;=\sum_{i=1}^{N}\left\{\log p\left({\hat{\theta }}_{i}\right)+\log p(z_{i}>0|{\hat{\theta }}_{i},s_{i})-\log p(z_{i}>0|s_{i})\right\}\\ & \;=\sum_{i=1}^{N}\left\{-\frac{1}{2}\log\left(\tau^{2}+\sigma_{i}^{2}\right)-\frac{{\hat{\theta }}_{i}-\theta }{2\left(\tau^{2}+\sigma_{i}^{2}\right)}-\log\Phi \left(u_{i}\right)+\log\Phi \left(v_{i}\right)\right\} \end{array}$$

with

$$u_{i}=\beta_{0}+\frac{\beta_{1}}{s_{i}},\;v_{i}=\frac{u_{i}+{\tilde{\rho }}_{i}\left({\hat{\theta }}_{i}-\theta \right)/\sqrt{\tau^{2}+\sigma_{i}^{2}}}{\sqrt{1-\rho_{i}^{2}}},\;\text{and}\;{\tilde{\rho }}_{i}=\frac{\sigma_{i}}{\sqrt{\tau^{2}+\sigma_{i}^{2}}}\rho .$$

During estimation, $\sigma_{i}$ is replaced by its estimate ${\hat{\sigma }}_{i}=s_{i}/\sqrt{1-c_{i}^{2}\rho^{2}}$ and $c_{i}^{2}=\phi \left(u_{i}\right)/\Phi \left(u_{i}\right)\left\{u_{i}+\phi \left(u_{i}\right)/\Phi \left(u_{i}\right)\right\}$. When the number of subjects in each studies are large, $\sigma_{i}$ can be replaced by $s_{i}$, the estimated SEs of the observed studies [16].

This log‐likelihood remains consistent with the NN model in the absence of PB (when ρ=0). Since the likelihood for estimating parameters $\beta_{0}$ and $\beta_{1}$ tends to plateau, Copas and Shi [16] proposed a sensitivity analysis approach by fixing various pairs of values for $\left(\beta_{0},\beta_{1}\right)$. Fixing these values sets the selection probabilities $P\left(Z_{i}>0|s_{i}\right)$, which then allows the remaining model parameters (θ,τ,ρ) in the log‐likelihood (5) to be estimated via the ML method. In addition, the number of unpublished studies can be approximated as

$$M=\sum_{i=1}^{N}\frac{1-P\left(Z_{i}>0|s_{i}\right)}{P\left(Z_{i}>0|s_{i}\right)}.$$

Since the true values of $\left(\beta_{0},\beta_{1}\right)$ are unknown, sensitivity analysis helps evaluate how the estimates of parameters of interest change over a plausible range of values for $\left(\beta_{0},\beta_{1}\right)$. Direct interpretation of $\left(\beta_{0},\beta_{1}\right)$ may be challenging, while one can instead assess how the estimates change with different numbers of unpublished studies implied by M. Hereinafter, we refer to this sensitivity analysis method as the Copas‐Shi method.

In addition [16], Copas proposed a selection equation related to the sizes of studies for dealing with PB based on the NN model [15]. In the context of data summarized in contingency tables (such as Table 1), $\chi^{2}$ test is usually conducted to explore the effect of treatment on the outcomes, and the association between treatment and outcomes is measured by the phi coefficient, $\phi =\sqrt{\chi^{2}/n}$, which has an asymptotic variance of Var(ϕ)=1/n. Thus, an alternative Gaussian variable was defined to model selective publication process, that is,

(6) $$Z_{i}=\gamma_{0}+\gamma_{1}n_{i}^{1/2}+\delta_{i},$$

where $n_{i}=n_{i0}+n_{i1}$ is the total samples size of study i, and $\delta_{i}$ is the residual term following N(0,1).

With selective publication of studies, the log‐likelihood conditional on the published studies was derived (see Equation 7 in Copas [15]); this method is referred to as the Copas‐N method, hereinafter. We presented a brief review of the Copas‐N method in Section A of Supporting Information. However, the conditional log‐likelihood of the Copas‐N method is still dependent on the normal approximation, which requires continuity correction. Moreover, in the absence of PB, the conditional log‐likelihood does not exactly reduce to that of the NN model (3).

## 3 Generalized Linear Mixed Models for Rare‐Event Meta‐Analysis

### 3.1 Rare‐Event Meta‐Analysis of Odds Ratios

When the number of events in the contingency table is close to or contains zero, the normal approximation using model (1) will be invalid for modeling the observed lnORs, leading to bias in the estimation of parameters of interest. Therefore, modeling the number of events using exact distributions is recommended [4]. For the estimation of the overall lnOR, Van Houwelingen et al. [33] and Stijhnen et al. [4] proposed the HN model. Conditional on the total number of events ($y_{i}=y_{i0}+y_{i1}$) and the subjects of each group ($n_{i0}$ and $n_{i1}$), the number of events in the treatment group is modeled by the Fisher's noncentral hypergeometric distribution: [34, 35]

(7) $$Y_{i1}|\theta_{i}∼\text{fnchypg}\left(n_{i0},n_{i1},y_{i},\exp\left(\theta_{i}\right)\right),$$

where $\exp\left(\theta_{i}\right)$ indicates the true study‐specific ORs. Then, at the between‐study level, the distribution of $\theta_{i}$ is assumed to follow the normal distribution (2). By combing model (7) and (2), the likelihood of the HN model is derived as follows:

(8) $$\begin{array}{cc} L\left(\theta ,\tau \right) & =\prod_{i=1}^{N}\int_{-\infty }^{\infty }L_{i}\left(\theta_{i}\right)\frac{1}{\tau }\phi \left(\frac{\theta_{i}-\theta }{\tau }\right)d\theta_{i}\;\text{with}\;L_{i}\left(\theta_{i}\right)\\ & =\frac{\left(\begin{array}{l} n_{i1}\\ y_{i1} \end{array}\right)\left(\begin{array}{l} n_{i0}\\ y_{i0} \end{array}\right)\exp\left(\theta_{i}y_{i1}\right)}{\sum_{k\in K}\left(\begin{array}{c} n_{i1}\\ k \end{array}\right)\left(\begin{array}{c} n_{i1}\\ y_{i}-k \end{array}\right)\exp\left(\theta_{i}k\right)}. \end{array}$$

where K is the set of all possible values of $y_{i1}$ constrained by the marginal totals $n_{i0}$ and $n_{i1}$ in Table 1, and θ is the overall lnOR of interest which can be estimated by the ML method with numerical integration [4].

When the number of subjects is large in the studies, estimation under the HN model, particularly the numerical integration, can be computationally demanding [5]. When the total occurrences of events are much smaller than the total subjects in the treatment and control groups, an approximation version of the HN model is possible to implement. In this case, the probability (7) can be approximated by the following binomial distribution, which simplifies computation and model fitting: [4, 9]

(9) $$Y_{i1}|\theta_{i}∼\mathrm{Bin}\left(y_{i},\frac{\exp\left\{\log\left(n_{i1}/n_{i0}\right)+\theta_{i}\right\}}{1+\exp\left\{\log\left(n_{i1}/n_{i0}\right)+\theta_{i}\right\}}\right).$$

The likelihood to estimate θ is constructed in the same way by replacing the within‐study likelihood $L_{i}\left(\theta_{i}\right)$ with that of the binomial distribution (9), leading to the following conditional BN (CBN) model: [5]

(10) $$\begin{array}{cc} L\left(\theta ,\tau \right) & =\prod_{i=1}^{N}\int_{-\infty }^{\infty }L_{i}\left(\theta_{i}\right)\frac{1}{\tau }\phi \left(\frac{\theta_{i}-\theta }{\tau }\right)d\theta_{i}\;\text{with}\;\\ L_{i}\left(\theta_{i}\right) & =\left(\begin{array}{l} y_{i}\\ y_{i1} \end{array}\right)\frac{\exp{\left\{\log\left(n_{i1}/n_{i0}\right)+\theta_{i}\right\}}^{y_{i1}}}{{\left[1+\exp\left\{\log\left(n_{i1}/n_{i0}\right)+\theta_{i}\right\}\right]}^{y_{i}}}. \end{array}$$

The estimation performances of the HN and CBN models have been extensively studied, and the CBN model (10) is generally considered more computationally feasible than the HN model (8) [4, 5, 9].

### 3.2 Rare‐Event Meta‐Analysis of Proportions

For meta‐analyses involving single‐group studies that estimate the overall log odds (equivalently, the logit‐transformed proportion) of the event, the binomial distribution is employed to model the number of events in the single group (e.g., the treatment group in Table 1), that is,

(11) $$Y_{i1}|\theta_{i}∼\mathrm{Bin}\left(n_{i1},\pi_{i}\right)\;\text{with}\;\pi_{i}=\frac{\exp\left(\theta_{i}\right)}{1+\exp\left(\theta_{i}\right)}$$

where $\theta_{i}=\text{logit}\left(\pi_{i}\right)$, and $\pi_{i}$ represents the true occurrence probability of event in each study, Under the same framework, the likelihood is derived by combining the within‐study likelihood of the binomial model (11) with the NN model (2):

(12) $$\begin{array}{cc} L\left(\theta ,\tau \right) & =\prod_{i=1}^{N}\int_{-\infty }^{\infty }L_{i}\left(\theta_{i}\right)\frac{1}{\tau }\phi \left(\frac{\theta_{i}-\theta }{\tau }\right)d\theta_{i}\;\text{with}\;L_{i}\left(\theta_{i}\right)\\ & =\left(\begin{array}{l} n_{i1}\\ y_{i1} \end{array}\right)\frac{\exp{\left(\theta_{i}\right)}^{y_{i1}}}{{\left\{1+\exp\left(\theta_{i}\right)\right\}}^{n_{i1}}}. \end{array}$$

To distinguish it from the CBN model (10) used for estimating the overall lnORs, we refer to this as the one‐sample binomial‐normal random‐effects (1SBN) model.

## 4 Sensitivity Analysis for Publication Bias in Generalized Linear Mixed Models

In this section, we introduce the proposed sensitivity analysis methods that deal with PB in rare‐events meta‐analyses based on the aforementioned HN and two BN models. Corresponding to Section 3, we consider the situation of PB, where the N published studies are regarded as biased sample from the population of related studies. To completely avoid continuity corrections for the zero entries and the estimation of SEs, it is natural to employ the latent Gaussian variable of the Copas‐N method [15], as shown in Equation (6), to model selective publication process. Given the observed sample sizes $n_{i}$ in Table 1, we define the Gaussian variable $Z_{i}$ for study i as follows:

(13) $$Z_{i}=\alpha_{0}+\alpha_{1}{n_{i}}^{1/2}+\delta_{i}$$

where $\alpha_{0}$ and $\alpha_{1}$ are constants and $\delta_{i}$ is residual with distribution N(0,1). Study i is published if and only if $Z_{i}>0$, aligning with the interpretation of latent variable (6). Based on the framework of the GLMM, it is difficult to re‐express the observed outcomes using the standard normal residuals. Thus, we directly model the joint distribution of study‐specific true effect sizes $\theta_{i}$ with residuals $\delta_{i}$ in (13), expressed as follows:

(14) $$\left(\begin{array}{c} \theta_{i}\\ \delta_{i} \end{array}\right)∼N\left(\left(\begin{array}{c} \theta \\ 0 \end{array}\right),\left[\begin{array}{cc} \tau^{2} & \tau \rho \\ \tau \rho & 1 \end{array}\right]\right),$$

where ρ indicates the correlation between $\theta_{i}$ and $\delta_{i}$, and ρ is estimated to be either positive or negative to indicate the overall direction of PB. When ρ>0, larger $\theta_{i}$ is associated with larger $\delta_{i}$, which consequently results in larger value of $Z_{i}$. When ρ=0, $\theta_{i}$ and $\delta_{i}$ become independent, indicating the absence of PB. Then, the conditional distribution of $\delta_{i}$ given $\theta_{i}$ is derived as follows:

$$\delta_{i}|\theta_{i}∼N\left(\rho \frac{1}{\tau }\left(\theta_{i}-\theta \right),1-\rho^{2}\right).$$

On the other hand, the probability of study i being published can be written by the probit model:

(15) $$P\left(Z_{i}>0|n_{i}\right)=\Phi \left(\alpha_{0}+\alpha_{1}{n_{i}}^{1/2}\right),$$

indicating that larger studies are more likely to be published. In the absence of PB, the likelihood conditional on the published studies with $Z_{i}>0$ is derived as follows:

(16) $$L\left(\theta ,\tau ,\rho ,\alpha_{0},\alpha_{1}\right)=\prod_{i=1}^{N}\int L_{i}\left(\theta_{i}|Z_{i}>0,n_{i}\right)\frac{1}{\tau }\phi \left(\frac{\theta_{i}-\theta }{\tau }\right)d\theta_{i}$$

with the conditional within‐study likelihood being

(17) $$\begin{array}{ll} L_{i}\left(\theta_{i}|Z_{i}>0,n_{i}\right) & =\frac{P\left(Z_{i}>0|\theta_{i},n_{i}\right)L_{i}\left(\theta_{i}\right)}{P\left(Z_{i}>0|n_{i}\right)}\\ & \;=\frac{P\left(\alpha_{0}+\alpha_{1}\sqrt{n_{i}}+\delta_{i}>0|\theta_{i},n_{i}\right)L_{i}\left(\theta_{i}\right)}{P\left(Z_{i}>0|n_{i}\right)}\\ & \;=\frac{\Phi \left\{\frac{\alpha_{0}+\alpha_{1}\sqrt{n_{i}}+\rho \left(\theta_{i}-\theta \right)/\tau }{\sqrt{1-\rho^{2}}}\right\}L_{i}\left(\theta_{i}\right)}{\Phi \left(\alpha_{0}+\alpha_{1}\sqrt{n_{i}}\right)}, \end{array}$$

where $L_{i}\left(\theta_{i}\right)$ indicates the exact within‐study likelihoods introduced in Section 3. When ρ≠0, whether a study gets published is related with $\theta_{i}$, inducing PB when estimating the overall θ. When ρ=0, the conditional within‐likelihood (17) reduces to $L_{i}\left(\theta_{i}\right)$, implying that study outcomes are not influenced by any unpublished studies. In the Copas‐N and Copas‐Shi methods [15, 16], the constant parameters are fixed at several values for sensitivity analysis. Similarly, we also adopt the sensitivity analysis approach by fixing sensitivity parameters $\left(\alpha_{0},\alpha_{1}\right)$ while estimating the other parameters (θ,τ,ρ). Since the sensitivity parameters $\left(\alpha_{0},\alpha_{1}\right)$ are challenging in their interpretations, we considered their transformations in sensitivity analysis. Let $n_{\min}=\min\left\{n_{i}\right\}$ and $n_{\max}=\max\left\{n_{i}\right\}$ be the minimum and maximum numbers of subjects among N published studies; $P_{\min}$ and $P_{\max}$ denote the probabilities of publishing a study with $n_{\min}$ and $n_{\max}$ subjects, respectively, (practically, $P_{\min}\leq P_{\max}$). With model (15), $P_{\min}=\Phi \left(\alpha_{0}+\alpha_{1}\sqrt{n_{\min}}\right)$ and $P_{\max}=\Phi \left(\alpha_{0}+\alpha_{1}\sqrt{n_{\max}}\right)$ hold. Given prespecified values of $\left(P_{\min},P_{\max}\right)$, the constants $\left(\alpha_{0},\alpha_{1}\right)$ are derived by

(18) $$\alpha_{1}=\frac{\Phi^{-1}\left(P_{\max}\right)-\Phi^{-1}\left(P_{\min}\right)}{\sqrt{n_{\max}}-\sqrt{n_{\min}}}\;\text{and}\;\alpha_{0}=\Phi^{-1}\left(P_{\max}\right)-\alpha_{1}\sqrt{n_{\max}},$$

and then the other parameters (θ,τ,ρ) in (16) are estimated by the ML method. The number of unpublished studies is approximated in a similar way by [26]

$$M=\sum_{i=1}^{N}\frac{1-P\left(Z_{i}>0|n_{i}\right)}{P\left(Z_{i}>0|n_{i}\right)}.$$

## 5 Simulation Studies

### 5.1 Simulation Designs

Simulation studies were conducted to assess the performance of the proposed sensitivity analysis methods in adjusting PB in the HN and two BN (CBN and 1SBN) models under specified sensitivity parameters. We conducted separate simulations for meta‐analysis of ORs and proportions.

We considered meta‐analyses with small and moderate numbers of population studies (S=15,50); the population studies contained the published and unpublished studies. The overall true effect size, either the lnOR or the log odds, was set as θ=−2. The between‐studies variances were varied to reflect small to large heterogeneity, with values set as $\tau^{2}=0.1$, 0.3, or 0.7. According to Equation (14), we generated the study‐specific true effect size ($\theta_{i}$) and residuals $\delta_{i}$. For each study i, we considered different scenarios for the total subjects ($n_{i}$), which were sampled from uniform distributions: U[30,60], U[50,200], and U[500,700]. The scenarios with U(500,700) might be impractical in real‐world settings, but it was included to assess the approximation performance of the HN and CBN models under idealized conditions. The total sample size $n_{i}$ was then divided into treatment and control groups using different allocation ratios: $n_{i1}:n_{i0}=1:1$ or 2:1.

In each meta‐analysis, we simulated the data of each population study i(i=1,2,…,S) in the form of a contingency table (Table 1) with small numbers of events. For meta‐analysis of ORs, we considered two data‐generating processes to create the number of events in each group. The first generating process was based on the contrast‐based model and followed the HN model (8) as the true data‐generating mechanism. Given a fixed total number of event ($y_{i}$) generated from U[5,15] and the number of subjects in each group ($n_{i1}$ and $n_{i0}$), the number of events in treatment groups ($y_{i0}$) were sampled from the hypergeometric distribution (7); the remaining cells in the contingency table were derived accordingly. We referred to this as the HN model‐based data‐generating process.

The second generating process reflected the arm‐based design of randomized controlled trials by separately generating the number of events in either group from binomial distributions without fixing the total number of events.; we referred to this process as the two‐sample binomial normal (2SBN) model‐based data‐generating process. The bivariate BN model was adopted to generated studies. Specifically, the probabilities of event for each study i in the control group, denoted by $p_{i0}$, were generated from a normal distribution $N\left(p_{0},\tau^{2}/4\right)$ (following model 4 in Jackson et al. [9]), where three scenarios for $p_{0}=\left\{0.2,0.1,0.002\right\}$ were considered corresponding to different total sample size settings. By setting the true lnOR as θ=−2, the event probability of each study in the treatment group ($p_{i1}$) was derived accordingly. Then, given the number of subjects in each group ($n_{i1}$ or $n_{i0}$), the number of events in each group was independently generated from a binomial distribution: $y_{\mathrm{ij}}∼\mathrm{Bin}\left(n_{\mathrm{ij}},p_{\mathrm{ij}}\right)$, where j={0,1}, and $p_{\mathrm{ij}}$ indicated the true probability of event in each group for study i.

For meta‐analysis of proportions in a single group, it was difficult to generate rare events based on a large number of subjects. Thus, we considered the scenarios of $n_{i}$ sampled from U[15,30] and U[25,100] as the total number of subjects, and the number of events was generated based on the 1SBN mode (12) given the true log odds being θ=−2. This is referred to as the 1SBN model‐based data‐generating process. The summary of simulation scenarios for meta‐analysis of ORs and proportions was presented in Table 2 and Table 3, respectively.

**TABLE 2:** Scenarios for simulating meta‐analysis of odds ratios.

True lnOR (θ)	ρ	Total event ($y_{i}$)	$\left(P_{\max},P_{\min}\right)$	Total subjects ($n_{i}$)	$p_{0}$	*T*:*C*	$\tau^{2}$
−2	0.8	[5, 15]	(0.99, 0.20)	[30, 60]	0.2	1:1	0.1
							0.3
							0.7
						2:1	0.1
							0.3
							0.7
				[50, 200]	0.1	1:1	0.1
							0.3
							0.7
						2:1	0.1
							0.3
							0.7
				[500, 700]	0.002	1:1	0.1
							0.3
							0.7
						2:1	0.1
							0.3
							0.7

*Note:* T:C indicates Treatment:Control.

**TABLE 3:** Scenarios for simulating meta‐analysis of proportions.

True log odds (θ)	ρ	$\left(P_{\max},P_{\min}\right)$	Total subjects ($n_{i}$)	$\tau^{2}$
−2	0.8	(0.99, 0.20)	[15, 30]	0.1
				0.3
				0.7
			[25, 100]	0.1
				0.3
				0.7

To simulate selective publication process, we selected N studies as the published ones from the S population studies using selection function (13). The detailed selection process is summarized as follows:

1. in each meta‐analysis, given presepecified values on sensitivity parameters $\left(P_{\min},P_{\max}\right)=\left(0.2,0.99\right)$, parameters $\left(\alpha_{0},\alpha_{1}\right)$ were calculated according to Equation (18);
2. the random variable $Z_{i}$ was generated using Equation (13) for each studies given sample sizes ($n_{i}$) and the generated $\delta_{i}$;
3. studies were selected as published ones if $Z_{i}>0$; otherwise, they were treated as unpublished.

All simulation scenarios were repeated 1000 times, which means the performances of various methods were summarized based on 1000 meta‐analyses. We evaluated the performance of the NN, HN, and BN (CBN and 1SBN) models using both the full population studies (published and unpublished studies) and the subset of published studies only. In the NN model, the continuity correction was conducted by adding 0.5 to each cell of the contingency table for the studies with zero entries. The estimates based on the full population and the published studies were denoted by subscripts P and O, respectively (e.g., HN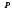, HN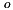), and the differences between the two types of estimates reflected the magnitude of PB under each model. To assess the ability of adjusting PB, we compared the performance of the proposed methods with the Copas‐N^15^ and Copas‐Shi^16^ methods, which adopted the Copas‐Heckman‐type selection functions and rely on the NN model, and the method of Hu et al. [30], which adopted the t‐statistic‐based selection model to adjust PB in the HN and BN models. In sensitivity analyses, sensitivity parameters were set to their true values for estimating the other parameters of interest. While this strategy is not feasible in real‐world applications, it was used exclusively in simulation studies. To differentiate results derived from the various models, we denoted the proposed method with the superscript Prop (e.g., HN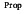). The methods of Copas‐N [15] and Copas‐Shi [16] were denoted by CN and CS, respectively; the methods of Hu et al. [30], were denoted with the superscript Hu (e.g., HN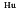).

In addition to the true selective process (13) in the simulation scenarios, we considered the misspecified selection process generated by the Hedge‐type selection model [36] where the publication of studies followed a step function based on the p values of studies. The scenarios of step functions were summarized in Section B.1 of Supporting Information. The simulation studies were implemented by R with further details provided in Section B.2 of Supporting Information. All the reproducible R codes are available on GitHub.

### 5.2 Result 1: Meta‐Analysis of OR Under the HN Model‐Based Data‐Generating Process

Under all scenarios, a large proportion of studies included rare events (defined as fewer than three events in either group). The occurrences of rare events in the population and published studies were summarized and presented, respectively in the HN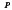 and HN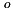 columns of Table B1 of Supporting Information. The estimates of the overall lnOR (i.e., θ) were summarized in Table 4. When population data were generated by the HN model, the estimates of the HN model using population studies (HN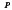) yielded minimal bias, as expected. In contrast, the estimates of NN model (NN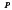) showed substantial bias across all rare‐event meta‐analysis scenarios. When the number of studies and subjects in studies was sufficiently large, estimates by the CBN model (CBN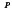) tended to have reduced bias and closely approximated the HN model results. When estimations were based only on the published studies, substantial PB was observed across all three models, reflected in the biased estimates NN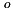, HN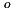, and CBN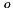. The magnitude of PB was especially increased when the between‐study heterogeneity parameter τ was moderately large. When the number of population studies increased, the impact of PB also increased especially when τ was large and the number of subjects were small.

**TABLE 4:** Averages of estimation bias of the lnOR (θ=−2) among different models under the HN model‐based data‐generating process.

S	Patients	*T*:*C*	$\tau^{2}$	N	NN 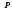	NN 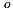	CN (CP)	CS (CP)	HN 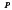	HN 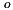	HN 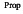 (CP)	HN 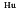 (CP)	CBN 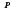	CBN 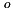	CBN 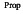 (CP)	CBN 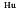 (CP)
15	*U*[30, 60]	1:1	0.1	10.6	18.1	24.3	−7.5 (75.6)	−40.6 (66.5)	−3.2	0.3	−10.0 (93.7)	4.3 (93.7)	39.6	37.9	24.4 (81.8)	41.2 (60.7)
			0.3	10.6	21.4	33.5	−4.8 (74.4)	−32.0 (71.7)	−3.2	11.2	−1.7 (91.5)	12.3 (89.1)	39.0	46.9	31.4 (79.2)	47.9 (57.4)
			0.7	10.6	28.1	44.3	−0.2 (79.4)	−23.5 (78.2)	−2.0	17.2	2.2 (91.4)	17.9 (87.3)	39.6	51.3	34.9 (77.3)	51.8 (55.1)
		2:1	0.1	10.6	5.2	12.6	−10.1 (78.3)	−48.9 (53.8)	−1.7	6.0	−2.9 (91.8)	7.1 (92.7)	53.8	52.8	41.7 (65.4)	54.2 (31.7)
			0.3	10.6	8.3	19.9	−10.9 (78.0)	−43.6 (51.3)	−0.0	12.1	1.5 (89.8)	13.1 (87.8)	53.9	57.8	44.6 (63.5)	58.4 (30.0)
			0.7	10.5	10.5	28.8	−6.8 (77.0)	−40.1 (54.1)	−1.5	19.4	3.3 (90.9)	19.9 (86.4)	53.2	63.4	45.4 (66.4)	62.8 (34.3)
	*U*[50, 200]	1:1	0.1	10.8	25.2	30.3	−6.5 (76.4)	−24.8 (87.7)	−5.9	−1.7	−12.1 (92.2)	2.1 (95.2)	9.0	12.4	−0.9 (92.0)	15.2 (89.1)
			0.3	10.8	32.7	42.4	1.1 (78.3)	−13.7 (89.0)	−3.3	8.1	−5.6 (92.6)	10.8 (88.9)	11.7	20.8	5.7 (89.5)	23.0 (81.0)
			0.7	10.9	43.0	56.7	8.3 (75.9)	−0.9 (85.1)	1.2	20.0	5.1 (88.8)	20.9 (84.2)	15.6	31.3	14.1 (86.2)	32.9 (74.4)
		2:1	0.1	10.9	13.4	19.7	−10.2 (75.4)	−38.7 (57.0)	−1.3	5.3	−4.5 (91.4)	6.5 (93.7)	18.6	20.5	9.7 (89.3)	22.9 (79.9)
			0.3	10.8	16.1	26.3	−11.4 (75.2)	−34.1 (58.8)	−1.1	9.6	−3.1 (91.2)	10.3 (89.6)	18.0	24.7	9.9 (90.6)	26.9 (73.5)
			0.7	10.8	22.1	37.9	−3.6 (77.5)	−25.0 (62.1)	0.1	19.4	3.2 (90.8)	19.5 (85.5)	19.6	34.6	16.3 (86.9)	35.2 (72.5)
	*U*[500, 700]	1:1	0.1	10.4	29.9	35.0	−3.9 (76.1)	−17.0 (92.7)	−8.2	−1.5	−13.4 (93.4)	1.8 (93.7)	−5.8	−0.1	−11.1 (93.6)	5.1 (93.0)
			0.3	10.4	37.5	47.0	4.7 (78.4)	−7.4 (91.8)	−2.9	11.5	−2.0 (91.2)	13.2 (88.2)	−0.2	13.4	0.4 (91.0)	14.6 (87.7)
			0.7	10.4	47.0	61.6	9.9 (80.0)	7.4 (86.5)	−1.2	20.4	1.9 (91.1)	21.6 (84.1)	1.3	22.8	5.0 (90.0)	24.9 (81.2)
		2:1	0.1	10.4	15.7	21.5	−14.1 (72.2)	−34.4 (66.8)	−5.1	2.1	−10.8 (92.5)	3.3 (93.9)	−2.0	4.7	−7.5 (92.8)	6.5 (92.4)
			0.3	10.5	22.1	32.7	−9.7 (77.3)	−24.0 (71.6)	−2.0	11.6	−1.0 (92.1)	12.9 (88.6)	1.3	14.3	2.0 (91.6)	15.8 (86.1)
			0.7	10.4	28.3	44.2	−1.6 (77.8)	−13.1 (77.5)	−0.4	21.5	4.1 (90.4)	21.5 (83.3)	2.7	24.2	7.5 (89.9)	24.6 (81.0)
50	*U*[30, 60]	1:1	0.1	35.9	19.0	26.5	−10.4 (87.9)	−30.8 (43.0)	−2.0	6.0	−2.8 (93.5)	6.9 (91.8)	40.7	43.8	32.1 (62.1)	44.5 (18.1)
			0.3	36.0	24.4	36.4	−6.7 (87.8)	−22.4 (58.4)	−0.3	13.8	1.8 (92.8)	14.3 (83.2)	42.1	49.6	35.9 (54.6)	51.0 (12.1)
			0.7	36.1	30.2	48.1	−2.4 (90.3)	−12.2 (75.5)	−0.2	21.6	3.2 (94.2)	21.7 (77.0)	42.0	55.9	38.4 (58.8)	56.6 (15.7)
		2:1	0.1	35.8	6.3	13.6	−12.1 (88.2)	−61.2 (11.3)	−0.5	7.6	−0.8 (94.4)	7.6 (92.8)	54.3	55.6	45.2 (13.9)	55.9 (0.5)
			0.3	36.0	8.6	20.8	−11.5 (86.0)	−58.5 (9.6)	−0.4	13.1	1.7 (92.7)	13.3 (84.6)	54.5	59.4	46.8 (17.3)	59.9 (1.3)
			0.7	35.9	10.7	29.3	−10.4 (86.3)	−53.1 (14.9)	−0.9	20.2	1.9 (92.2)	20.1 (77.8)	54.4	64.1	46.9 (26.1)	64.2 (3.1)
	*U*[50, 200]	1:1	0.1	36.9	27.8	34.1	−6.4 (90.1)	−8.9 (89.0)	−2.4	5.3	−3.4 (92.9)	6.3 (92.2)	12.9	17.7	7.7 (91.3)	18.9 (73.5)
			0.3	36.9	34.6	44.6	−3.4 (90.1)	1.0 (89.7)	−0.5	12.4	0.2 (93.8)	13.1 (86.0)	15.0	25.0	11.1 (90.3)	25.7 (65.4)
			0.7	36.9	42.1	56.5	3.1 (90.3)	13.5 (75.5)	−0.4	19.5	3.1 (92.3)	19.5 (79.8)	15.1	31.3	13.7 (86.9)	31.2 (61.3)
		2:1	0.1	37.0	13.9	20.6	−11.9 (84.3)	−41.5 (9.4)	−0.9	6.3	−1.4 (94.8)	6.5 (90.6)	19.6	22.8	13.1 (86.0)	23.3 (55.6)
			0.3	37.0	18.9	30.1	−9.0 (87.4)	−34.7 (15.4)	0.7	13.4	2.9 (93.0)	13.7 (82.2)	20.5	29.2	16.3 (84.2)	29.7 (45.0)
			0.7	37.0	22.8	39.3	−6.9 (88.1)	−27.3 (31.4)	−0.1	19.9	2.4 (93.7)	19.9 (78.1)	19.8	34.7	15.7 (86.6)	34.9 (43.8)
	*U*[500, 700]	1:1	0.1	35.3	31.7	37.5	−5.1 (90.1)	−1.8 (93.9)	−3.5	4.2	−5.6 (94.2)	5.8 (92.0)	−0.1	7.0	−2.7 (94.7)	8.1 (89.6)
			0.3	35.3	38.8	48.7	−0.9 (90.2)	9.2 (87.9)	−1.7	13.4	0.9 (91.9)	13.9 (84.2)	1.0	15.6	3.3 (92.8)	16.4 (81.2)
			0.7	35.3	48.0	62.4	5.6 (90.3)	24.0 (59.4)	0.3	22.2	4.6 (93.5)	22.5 (77.1)	2.9	24.6	7.4 (92.8)	25.0 (72.4)
		2:1	0.1	35.4	18.6	25.2	−12.0 (85.6)	−27.6 (35.0)	−1.3	7.3	−1.9 (93.3)	7.6 (90.8)	2.1	10.3	1.5 (94.4)	11.0 (86.2)
			0.3	35.4	24.1	34.8	−8.9 (87.5)	−21.1 (54.1)	−0.5	14.2	2.7 (92.6)	14.4 (80.6)	3.0	17.3	6.0 (90.4)	17.8 (75.0)
			0.7	35.3	29.4	45.6	−5.4 (88.3)	−12.2 (73.1)	−0.4	22.1	3.1 (92.9)	22.2 (73.7)	3.0	25.1	6.2 (91.7)	25.2 (67.6)

*Note:* All estimates were multiplied by 100. Results of NN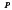, HN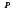, and CBN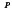 are based on the population studies; results of NN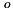, HN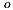, and CBN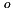 are based on the published studies; CN and CS indicate the Copas‐N and Copas‐Shi methods; HN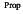 and CBN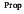 indicate the proposed HN or CBN model‐based sensitivity analysis methods; HN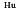 and CBN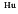 indicate the t‐statistic‐based sensitivity analysis methods [30]; CP indicates the coverage probability.

In the sensitivity analyses, the Copas‐Shi method generally resulted in the largest bias. In contrast, the proposed HN model‐based method (HN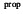) achieved small bias, especially when τ is neither very large nor small, indicating that the proposed method successfully adjusted PB. The proposed CBN model‐based method (CBN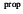) derived larger bias than HN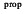 mainly due to the model misspecification in the data generating process. However, when the number of subjects was large and τ was small, the bias in the CBN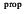 decreased, indicating improvements in approximations. Notably, the Copas‐N method also derived small bias; however, the coverage probabilities impaired, and when τ was small or subjects in the two groups were unbalanced, the bias was non‐negligible. The proposed HN model‐based method appeared robust against group imbalance, whereas the other methods showed increased bias when imbalance was present. Due to the misspecification of selection process, the t‐statistic‐based sensitivity analysis [30] (HN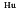 and CBN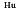) were biased. Even so, the HN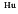 method still achieved less bias than the Copas‐Shi method. In particular, the HN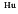 method had less bias when τ was small. The proposed HN model‐based method achieved coverage probabilities closer to the nominal level than the competing methods, although some coverage probabilities did not reach nominal levels, possibly due to imperfect specification of $\left(P_{\min},P_{\max}\right)$ in the sensitivity analyses.

The CBN model was introduced to reduce computational complexity and mitigate non‐convergence issues. Therefore, we investigated the proportions of estimations that successfully converged (out of 1000 runs), summarized as convergence rates in Table B3 of the Supporting Information. Results showed that the NN model had near‐perfect convergence rates, whereas the HN and CBN models, especially in the proposed methods, showed comparatively lower convergence rates; however, when the number of studies was not too small, satisfactory convergence rates could still be obtained. On the other hand, convergence rates were comparable between the HN and CBN models across the simulation settings. The estimation of τ was additionally investigated and summarized in Table B4 of Supporting Information. The proposed methods demonstrated less bias in estimating τ, while the Copas‐N method tended to overestimate and the Copas‐Shi method tended to underestimate τ. When selection function was misspecified under the Hedge‐type selection model, greater impact of PB were found in the HN model. Due to the misspecification of selection functions and sensitivity parameters, all the methods failed to adjust PB (Table B5 in Supporting Information).

### 5.3 Result 2: Meta‐Analysis of Odds Ratios Under the 2SBN Model‐Based Data‐Generating Process

Under the generating process of the 2SBN model, large proportions of rare events were derived; the proportions decreased when the number of total subjects was large. However, the rare events still accounted for relatively large proportions in the population and published samples, shown as the 2SBN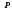 and 2SBN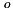 in Table B1 of Supporting Information. This data‐generating process followed neither the HN nor the CBN models; thus, estimations based on the population studies (HN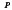 and CBN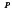) gained more bias due to the misspecification of model. The average estimates of the overall lnOR, θ, in Table 5 showed that the HN model (both HN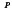 and HN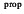) generally produced less bias than other methods; with bias tending to decrease as τ became smaller. Estimates from the CBN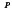 and CBN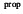 models were larger than those from the HN models but tended to decrease as the number of subjects increased. When τ was small, the bias in CBN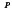 and CBN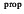 could be decreased. On the other hand, both the Copas‐N and Copas‐Shi methods failed to adequately adjust for PB, with the Copas‐N method exhibiting greater bias. The method of Hu et al. [30] showed less biased when τ was small, while this method were still biased than the proposed methods in most scenarios. Compared to the other methods, the proposed methods, especially the HN model‐based method, achieved coverage probabilities closer to the nominal level for estimating the lnOR.

**TABLE 5:** Averages of estimation bias of the lnOR (θ=−2) among different models under the 2SBN model‐based data‐generating process.

S	Patients	*T*:*C*	$\tau^{2}$	N	NN 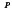	NN 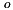	CN (CP)	CS (CP)	HN 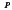	HN 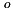	HN 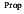 (CP)	HN 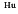 (CP)	CBN 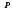	CBN 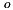	CBN 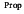 (CP)	CBN 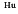 (CP)
15	*U*[30, 60]	1:1	0.1	10.6	66.2	65.8	66.4 (55.4)	58.5 (94.7)	−19.9	−13.6	−29.9 (92.1)	−9.1 (96.0)	1.6	2.9	−13.2 (91.0)	7.5 (92.5)
			0.3	10.6	71.3	73.6	72.0 (49.4)	63.0 (91.1)	−10.4	1.0	−9.8 (89.7)	3.5 (92.8)	6.9	13.3	5.3 (83.5)	19.9 (85.8)
			0.7	10.6	77.5	82.4	72.1 (49.6)	66.0 (86.4)	−3.0	14.6	−3.4 (87.8)	16.1 (87.3)	15.1	30.8	13.7 (83.3)	32.5 (78.5)
		2:1	0.1	10.6	43.0	44.3	43.3 (67.3)	39.3 (94.0)	−11.7	−6.5	−17.0 (92.2)	−2.3 (94.6)	3.2	12.0	−2.1 (88.9)	13.9 (89.8)
			0.3	10.6	45.9	49.5	44.3 (69.2)	39.5 (95.2)	−5.2	4.4	−11.6 (93.0)	6.7 (93.1)	11.3	18.7	5.5 (88.4)	24.0 (86.8)
			0.7	10.5	56.6	63.3	48.8 (65.8)	47.1 (92.1)	5.7	22.4	3.8 (90.6)	23.4 (86.6)	23.3	38.6	20.5 (84.1)	40.5 (76.7)
	*U*[50, 200]	1:1	0.1	10.8	33.4	33.7	32.1 (76.5)	5.4 (93.1)	−6.4	−0.4	−8.8 (92.7)	1.6 (95.0)	9.4	14.3	6.4 (87.7)	17.6 (86.5)
			0.3	10.8	40.2	43.3	35.3 (73.4)	12.6 (88.2)	−3.2	7.6	−2.2 (89.8)	9.2 (89.4)	11.6	23.5	13.0 (85.6)	24.9 (79.4)
			0.7	10.9	52.2	57.9	38.8 (71.2)	23.5 (81.4)	3.9	19.4	7.4 (87.5)	20.1 (84.6)	21.3	35.6	23.6 (81.5)	35.6 (72.4)
		2:1	0.1	10.9	22.5	24.7	20.0 (74.9)	2.2 (84.8)	−2.1	3.6	−3.4 (90.5)	5.5 (93.1)	13.2	19.0	12.6 (82.7)	20.7 (84.0)
			0.3	10.8	29.3	33.4	22.6 (75.2)	6.3 (83.8)	1.2	11.0	3.2 (90.9)	12.2 (88.4)	17.5	27.0	18.0 (85.2)	26.6 (77.3)
			0.7	10.8	39.9	47.7	27.5 (74.8)	14.7 (81.4)	6.8	23.6	10.9 (87.2)	23.5 (83.1)	23.2	39.3	26.5 (79.9)	39.2 (70.5)
	*U*[500, 700]	1:1	0.1	10.4	9.8	15.3	20.9 (74.6)	−14.6 (58.5)	−0.7	5.9	−1.6 (90.4)	6.5 (88.0)	14.6	21.7	13.9 (81.1)	21.9 (65.2)
			0.3	10.4	16.7	27.7	23.4 (74.2)	−4.9 (72.0)	2.0	16.3	6.1 (87.2)	16.4 (81.7)	17.0	30.9	21.0 (77.5)	31.3 (58.9)
			0.7	10.4	22.5	39.3	26.5 (76.3)	10.6 (78.4)	2.7	24.8	8.6 (86.3)	24.6 (77.3)	17.5	38.7	23.6 (79.4)	39.0 (60.7)
		2:1	0.1	10.4	7.1	13.6	15.4 (76.3)	−12.9 (58.5)	0.6	8.0	1.0 (90.4)	8.5 (88.4)	15.8	23.1	16.3 (80.8)	23.6 (60.4)
			0.3	10.5	11.5	22.7	17.0 (75.7)	−8.0 (72.0)	2.1	15.8	6.1 (87.0)	15.6 (83.0)	17.1	30.0	20.5 (78.4)	30.4 (61.1)
			0.7	10.4	16.9	35.0	19.3 (71.8)	7.3 (81.9)	3.4	25.8	10.4 (85.0)	25.9 (77.0)	18.0	39.9	24.6 (77.2)	38.9 (62.4)
50	*U*[30, 60]	1:1	0.1	35.9	73.1	73.1	76.6 (9.8)	66.8 (48.7)	−7.2	−2.7	−13.6 (93.9)	1.0 (94.4)	10.4	15.1	3.4 (90.0)	18.1 (86.1)
			0.3	36.0	77.7	79.5	79.6 (8.8)	67.8 (42.6)	−1.6	6.6	−4.4 (92.6)	10.4 (91.3)	15.8	23.8	12.7 (88.6)	28.3 (77.2)
			0.7	36.1	86.7	91.7	84.9 (6.8)	71.8 (27.3)	6.5	24.3	9.8 (88.8)	25.7 (80.1)	25.5	42.1	28.5 (79.8)	43.8 (61.7)
		2:1	0.1	35.8	47.2	48.7	50.5 (40.4)	43.7 (84.7)	−4.1	2.6	−7.5 (93.9)	3.8 (92.3)	11.2	19.0	8.7 (88.8)	20.1 (84.3)
			0.3	36.0	52.3	56.0	53.9 (33.7)	46.8 (78.1)	1.5	12.0	1.8 (90.1)	14.6 (88.5)	19.5	29.7	18.1 (86.7)	31.8 (72.8)
			0.7	35.9	62.3	69.6	58.9 (28.5)	50.2 (67.2)	9.2	28.1	13.7 (87.7)	28.9 (79.3)	27.4	45.1	31.2 (78.5)	45.9 (57.8)
	*U*[50, 200]	1:1	0.1	36.9	39.3	39.9	43.8 (56.1)	13.7 (93.8)	−1.2	4.5	−1.8 (91.6)	5.2 (91.7)	14.3	19.5	13.8 (83.0)	21.4 (71.9)
			0.3	36.9	48.0	51.1	47.4 (51.4)	20.8 (82.1)	2.6	13.2	6.9 (91.2)	13.6 (84.2)	19.2	29.3	22.6 (78.1)	29.6 (61.0)
			0.7	36.9	57.5	63.0	52.9 (47.6)	31.5 (61.0)	3.7	20.1	8.9 (88.1)	20.3 (79.9)	20.8	36.4	25.8 (74.9)	36.5 (59.2)
		2:1	0.1	37.0	26.1	27.7	28.9 (70.6)	4.6 (90.6)	0.5	5.7	0.1 (92.5)	6.6 (91.8)	16.3	21.3	16.0 (81.3)	22.6 (66.8)
			0.3	37.0	33.8	38.0	31.6 (66.6)	10.1 (91.3)	4.5	14.5	6.9 (89.3)	14.8 (82.2)	20.5	29.9	23.1 (73.0)	30.6 (55.4)
			0.7	37.0	43.5	50.8	33.2 (65.4)	18.2 (82.7)	7.5	23.3	9.4 (90.8)	23.3 (76.8)	23.9	39.0	25.8 (77.2)	39.1 (50.7)
	*U*[500, 700]	1:1	0.1	35.3	13.1	19.2	34.5 (62.7)	−13.6 (56.4)	1.0	8.9	2.7 (91.4)	9.0 (80.7)	16.3	23.9	17.8 (67.8)	24.2 (30.1)
			0.3	35.3	17.8	28.5	31.7 (69.8)	−1.6 (82.9)	1.0	15.3	3.1 (91.5)	15.3 (74.0)	16.0	29.8	18.2 (74.8)	29.9 (34.6)
			0.7	35.3	25.6	41.6	34.7 (67.1)	17.5 (68.3)	2.4	24.2	5.0 (88.7)	24.2 (66.6)	17.2	38.2	20.1 (77.1)	38.2 (36.2)
		2:1	0.1	35.4	8.5	14.9	23.6 (69.5)	−15.4 (55.1)	1.3	9.1	3.0 (89.9)	9.0 (80.9)	16.6	24.1	18.1 (64.6)	24.0 (24.6)
			0.3	35.4	13.1	24.9	22.3 (74.4)	−5.6 (81.4)	2.4	16.9	3.7 (91.0)	16.9 (69.4)	17.1	31.3	19.0 (69.9)	31.2 (27.7)
			0.7	35.3	18.8	36.2	23.9 (74.4)	10.4 (82.6)	3.2	25.4	5.4 (89.0)	25.4 (63.8)	17.7	39.1	20.3 (77.2)	39.3 (33.7)

*Note:* All estimates were multiplied by 100. Results of NN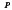, HN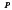, and CBN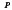 are based on the population studies; results of NN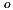, HN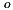, and CBN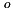 are based on the published studies; CN and CS indicate the Copas‐N and Copas‐Shi methods; HN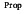 and CBN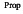 indicate the proposed HN or CBN model based sensitivity analysis methods; HN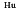 and CBN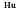 indicate the t‐statistic‐based sensitivity analysis methods [30]; CP indicates the coverage probability.

The convergence rates showed that the HN and BN models had comparable performance in estimation convergence (Table B6 in Supporting Information). The estimation results of $\tau^{2}$ indicated that the HN and BN models and the proposed methods gained less bias when the number of subjects and studies was large (Table B7 in Supporting Information). In contrast, overestimation and underestimation of $\tau^{2}$ were found in the Copas‐N and Copas‐Shi methods, respectively. Under the misspecified Hedge‐type selection model, all the methods failed to adjust PB (Table B8 in Supporting Information). When the number of subjects were large, the impact of PB became insignificant in the HN model under this data generating process.

### 5.4 Result 3: Meta‐Analysis of Proportions Under the 1SBN Model‐Based Data‐Generating Process

The scenarios of generating single‐group data were presented in Table 3. When the number of subjects was large, it was difficult to generate large proportions of rare events in the simulated studies; however, the amount of rare events still accounted for approximately 20% or more, as shown in Table B2 of Supporting Information. The average estimates of the log odds (i.e., θ) were summarized in Table 6. The results indicated that the 1SBN model yielded less bias than the NN model‐based on population studies; however, when the number of subjects was sufficiently large and rare events were not large, both models showed comparable performance with minimal estimation bias. Estimates based on published studies showed that PB increased with the increasing magnitude of τ. In the sensitivity analysis, the proposed 1SBN model (1SBN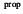)‐based method achieved less bias and coverage probability closer to the nominal level overall, indicating successful adjustment for PB. On the other hand, the Copas‐Shi method exhibited large bias and failed to adequately adjust for PB, while the Copas‐N method tended to perform better with less bias when the sizes of studies were large. Due to the misspecification of the selection function, the method of Hu et al. [30] also showed greater bias, especially when $\tau^{2}$ was large.

**TABLE 6:** Averages of estimation bias of the log odds (θ=−2) among different models under the 1SBN model‐based data‐generating process.

S	Patients	$\tau^{2}$	N	NN 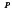	NN 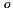	CN (CP)	CS (CP)	1SBN 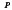	1SBN 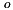	1SBN 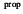 (CP)	1SBN 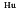 (CP)
15	U [15, 30]	0.1	10.6	18.6	22.3	−13.2 (78.8)	−19.0 (74.4)	−1.2	4.6	−1.6 (92.3)	5.4 (92.6)
		0.3	10.6	24.7	32.2	−11.0 (78.8)	−5.8 (79.7)	0.2	12.0	1.6 (91.5)	12.0 (87.5)
		0.7	10.6	29.7	42.4	−3.9 (80.3)	9.6 (73.6)	0.6	19.7	7.5 (90.9)	20.1 (81.5)
	*U*[25, 100]	0.1	10.8	7.6	11.8	−4.7 (80.0)	−13.1 (63.9)	−0.9	5.5	0.6 (93.1)	5.6 (90.8)
		0.3	10.8	10.3	18.1	−3.4 (79.4)	−5.0 (69.1)	−0.3	11.1	2.8 (89.8)	10.8 (82.6)
		0.7	10.8	12.8	26.3	−2.3 (79.8)	2.6 (73.7)	−0.3	17.8	4.4 (86.9)	18.1 (83.9)
50	*U*[15, 30]	0.1	36.0	20.6	25.0	−13.4 (86.1)	−3.8 (82.3)	−0.3	6.8	0.3 (95.0)	6.9 (88.4)
		0.3	36.1	25.4	34.0	−11.5 (86.6)	9.4 (75.6)	0.1	13.0	2.7 (92.2)	12.7 (79.3)
		0.7	36.2	30.1	43.6	−5.3 (90.7)	22.9 (35.9)	0.6	20.4	4.0 (92.5)	20.0 (72.8)
	*U*[25, 100]	0.1	37.0	8.7	13.0	−2.5 (93.4)	−11.3 (43.0)	0.3	6.6	2.0 (91.8)	6.7 (82.1)
		0.3	37.1	10.2	18.6	−3.0 (92.5)	−3.3 (75.1)	−0.3	11.4	1.5 (93.2)	11.3 (78.5)
		0.7	37.0	13.2	26.9	−2.5 (92.6)	6.0 (82.5)	0.2	18.7	1.4 (93.0)	18.6 (70.2)

*Note:* All estimates were multiplied by 100. NN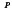 and BN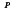 are the estimates based on the population studies; NN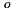 and BN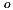 are the estimates based on the published studies; CN and CS are the Copas‐N and Copas‐Shi methods; 1SBN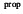 are the proposed 1SBN model‐based sensitivity analysis methods; 1SBN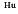 indicate the t‐statistic‐based sensitivity analysis methods [30]; CP indicates the coverage probability.

All methods generally achieved good convergence performance (Table B9 in Supporting Information); when the number of studies and the number of total subjects were small, the 1SBN model and the proposed method had some deficiencies in convergence rates. The summary of estimation of $\tau^{2}$ (Table B10 in Supporting Information) showed similar patterns: the proposed methods had less bias, while the Copas‐N method tended to overestimate and the Copas‐Shi method tended to underestimate $\tau^{2}$. Similarly, when studies were selected by the Hedge‐type selection model, all the methods were biased (Table B11 in Supporting Information).

## 6 Application

### 6.1 Example 1: Rare‐Event Meta‐Analysis of OR

We revisit the meta‐analysis of lnORs in Stijhnen et al. [4] This is the meta‐analysis of lnORs of catheter‐related bloodstream infection (CRBSI) between anti‐infective‐treated (AIT) central venous catheters and the standard catheter [37]. The data of this meta‐analysis were presented in Table C12 of Supporting Information, where five studies had zero events in the AIT catheter and one study had zero entries in both standard and AIT catheters. Stijnen et al. [4], compared meta‐analytical results by the NN model with those by the HN and CBN models without considering selective publication of studies. We reproduced the estimations of parameters in various models by maximizing likelihoods (4), (8), and (10). The SEs of the parameters were estimated by the inverse of the empirical Fisher information matrix following the ML theory. The HN and CBN models estimated the overall lnOR with 95% CI to be −1.353(−2.041,−0.665) and −1.303(−1.966,−0.639), respectively, which were consistent with the estimations reported by Stijnen et al. [4] The approximation in the results was attributed to the nature of the data, where the total number of CRBSI was relatively small compared to the total number of subjects. The NN model overestimated the overall lnOR to be −0.955(−1.415,−0.495), which turned to be biased towards zero [4]. The results showed that there were certain discrepancies between the NN model and the HN or CBN model, indicating that normal approximation in the NN model was questionable. These estimates derived by the ML method were identical with those generated by the R package metafor. The estimates of the NN model could be reproduced by the R function rma with method = “ML”, while the estimates of the HN and CBN models were identical with rma.glmm by setting model = “CM.EL” and model = “CM.AL”, respectively.

**FIGURE 1:**
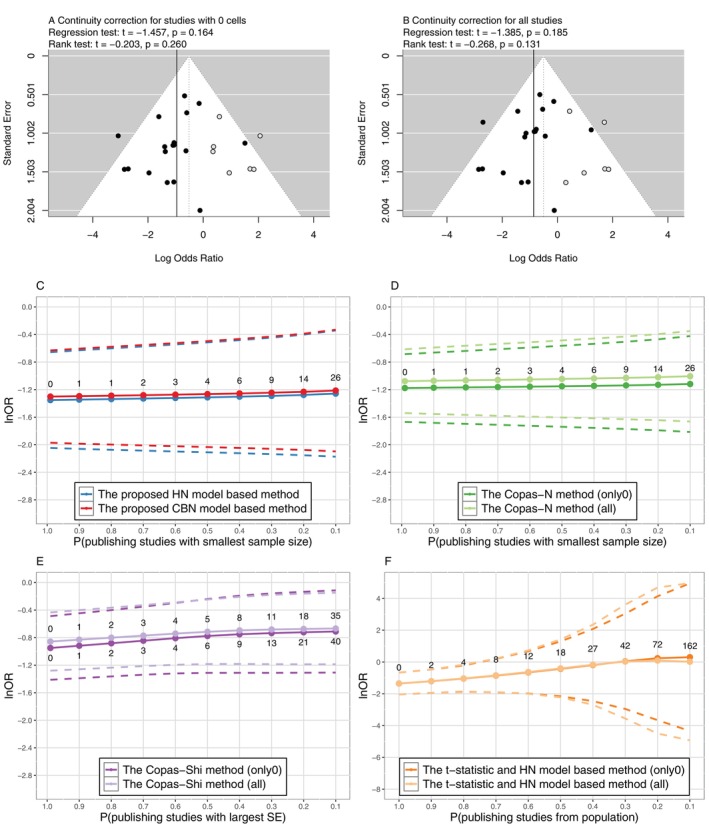
Comparison of sensitivity analysis methods for PB in Example 1. The values indicate the numbers of missing studies. Methods in panels (A), (B), (D), (E), and (F) need continuity correction, and panel (C) does not; only0 indicates continuity correction for studies with 0 cells only, while all indicates continuity correction for all studies. The dashed lines indicate 95% CI.

To evaluate PB in this meta‐analysis, we firstly employed the trim‐and‐fill method to adjust for the potential impact of PB. At the same time, Egger's regression and Begg's rank tests [38] were performed to assess funnel plot asymmetry, which suggested the presence of PB. These methods were developed based on the NN model. When there were zero entries, we conducted two approaches of continuity correction for the data: (a) adding 0.5 to all the cells of contingency tables for studies with zero entries only; (b) adding 0.5 for all the cells of all the studies. The results of trim‐and‐fill methods and the regression and rank tests corresponding to the two continuity correction methods were shown in Figure 1A,B, respectively. In Figure 1A, seven studies were filled, while in Figure 1B, six studies were filled. After accounting for the imputed studies, the estimates and 95% CIs of the lnOR were adjusted from −0.955(−1.415,−0.495) to −0.513(−1.010,−0.017) and from −0.861(−1.283,−0.440) to −0.506(−0.980,−0.031), respectively, with the continuity correction approaches (a) and (b). On the other hand, neither Egger's regression nor Begg's rank tests indicated significant asymmetry of the funnel plots.

The adjustments of PB were based on the symmetry of the funnel plot and required continuity correction, which might be subjective and biased for rare‐event meta‐analyses. Thus, we evaluated the potential PB on the lnOR by the proposed methods, which was free from continuity correction, and compared the results with other existing methods, including the Copas‐Heckman‐type sensitivity analysis methods [15, 16] and the t‐statistic selection function‐based sensitivity analysis methods [30] (hereinafter, the t‐statistic method) under both approaches of continuity correction. The t‐statistic methods [30] were also developed based on the HN and BN models; here, we only considered the exact HN model based one. The parameters (θ,τ,ρ) were then estimated by the ML method given pre‐specified values of sensitivity parameters. For the proposed method and the Copas‐N method [15], we set $\left(P_{\min},P_{\max}\right)=\left\{\left(0.99,0.999\right),\left(0.9,0.999\right),\left(0.8,0.999\right),\ldots ,\left(0.1,0.999\right)\right\}$, where $P_{\min}$ and $P_{\max}$ were probabilities of publishing studies with the largest and smallest numbers of subjects, respectively. For the Copas‐Shi method [16], the sensitivity parameters $P_{\min}$ and $P_{\max}$ indicated the probability of publishing studies with minimum and maximum SEs, respectively; then $\left(P_{\min},P_{\max}\right)$ were set as {(0.999,0.99),(0.999,0.9),(0.999,0.8),…,(0.999,0.1)}. For the t‐statistic method [30], the sensitivity parameter p was defined as the marginal probability of publishing studies from the population; thus, p=1,0.9,0.8,…,0.1 were set. When the probabilities in the sensitivity parameters tended to 1, the approximated numbers of unpublished studies were close to 0, indicating the situation of no PB.

The impact of PB was assessed via the changes of the estimated lnOR under various sensitivity analyses, and the results were presented in Figure 1C,D with the detailed estimations summarized in Table C13–C16 of Supporting Information. Given the fixed value of $P_{\max}=0.999$, $P_{\min}$ decreased from 0.99 to 0.1 (shown by x‐axis in Figure 1C–F), which indicated that the number of unpublished studies increased, as illustrated on the plots. By the proposed HN and CBN model‐based methods, the estimated lnOR increased subtly with increasing number of unpublished studies, indicating that PB had little influence on the estimation of the overall lnOR (Figure 1C). This conclusion was consistent with the results of funnel plots. On the other hand, the Copas‐N method overestimated the overall lnOR at each $P_{\min}$ (Figure 1D), and the Copas‐Shi method based on the NN model moreover induced an overestimation on the magnitude of PB (Figure 1E). Both methods were influenced by the different approaches of continuity correction. The t‐statistic method assessed potential PB using a selection function related to the t‐statistic (equivalently, p value) of the lnORs; it also gave a reasonable evaluation of PB and indicated that the estimated overall lnOR tended to be insignificant when the number of missing studies were greater than four; however, the computational time was about 6 times of the propose methods. We summarized the computational time of the proposed methods and the t‐statistic method in Table C31 of Supporting Information.

According to the results of the proposed methods, in summary, the estimations of the lnOR remained significant in the presence of unpublished studies, indicating that the meta‐analytical lnOR estimated by the original HN and CBN models were robust against potential PB in this example.

### 6.2 Example 2: Rare‐Event Meta‐Analysis of Odds Ratios

The second example included 16 trials examining the effectiveness of intravenous magnesium in the prevention of death following acute myocardial infarction, where eight studies had fewer than three events in the magnesium group and four studies in the control group. The data of this meta‐analysis were presented in Table C17 of Supporting Information.

**FIGURE 2:**
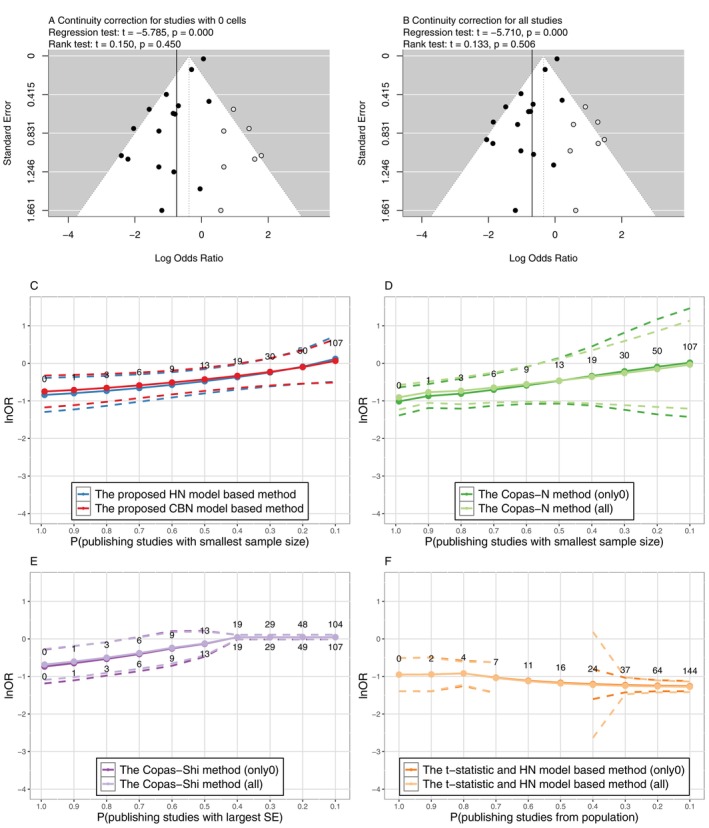
Comparison of sensitivity analysis methods for PB in Example 2. The values indicate numbers of missing studies. Methods in panels (A), (B), (D), (E), and (F) need continuity correction, and panel (C) does not; only0 indicates continuity correction for studies with 0 cells only, while all indicates continuity correction for all studies. The dashed lines indicate 95% CI.

In the absence of PB, the lnOR was estimated to be −0.844(−1.298,−0.390) by the HN model, −0.752(−1.177,−0.327) by the CBN model, and −0.746(−1.194,−0.299) by the NN model, respectively. To evaluate the impact of PB, various methods were compared with the results presented in Figure 2. Under two approaches of continuity correction, the funnel plots with Egger's test of asymmetry indicated the existence of PB; the trim‐and‐fill methods estimated seven unpublished studies and adjusted the overall lnOR from −0.746 to −0.375(−0.791,0.042) and from −0.691 to −0.348(−0.727,0.032), respectively (Figure 2A,B). However, the NN model and the trim‐and‐fill method had risks of overestimating the lnOR.

On the other hand, the proposed methods implied that the overall lnOR might become insignificant when there were more than 19 unpublished studies (Figure 2C). The estimates by the Copas‐Heckman‐type methods and the t‐statistic method were slightly influenced by the different approaches of continuity correction. Among these results, the Copas‐N method (Figure 2D) underestimated of the overall lnOR in the absence of PB (equivalently, $P_{\min}\approx 1$), while the Copas‐Shi method (Figure 2E) tended to overestimate the lnOR and yield unstable estimations when the number of unpublished studies were large. In this example, the t‐statistic method derived unstable estimations and a very long computation duration; the analysis of this method implied a decreasing estimation of the lnOR when the number of missing studies increased, as shown in Figure 2F. The detailed estimates by these methods were presented in Table C18–C21 of Supporting Information.

### 6.3 Example 3: Rare‐Event Meta‐Analysis of Proportions

In this case, we only adopted the data in the AIT catheter group, following the analysis by Stijnen et al. [4]; the parameter of interest is the overall log odds of CRBSI; a lower log odds indicated a lower probability of infection, which might be the preferable reports if one supported the AIT catheter. The number of events ranged from 0 to 6, while the number of subjects ranged from 44 to 345. Without considering PB, the log odds of CRBSI and its 95% CI were estimated to be −4.812(−5.508,−4.116) and −4.238(−4.793,−3.682) by maximizing the likelihoods of the 1SBN model (10) and the NN model (4), respectively.

**FIGURE 3:**
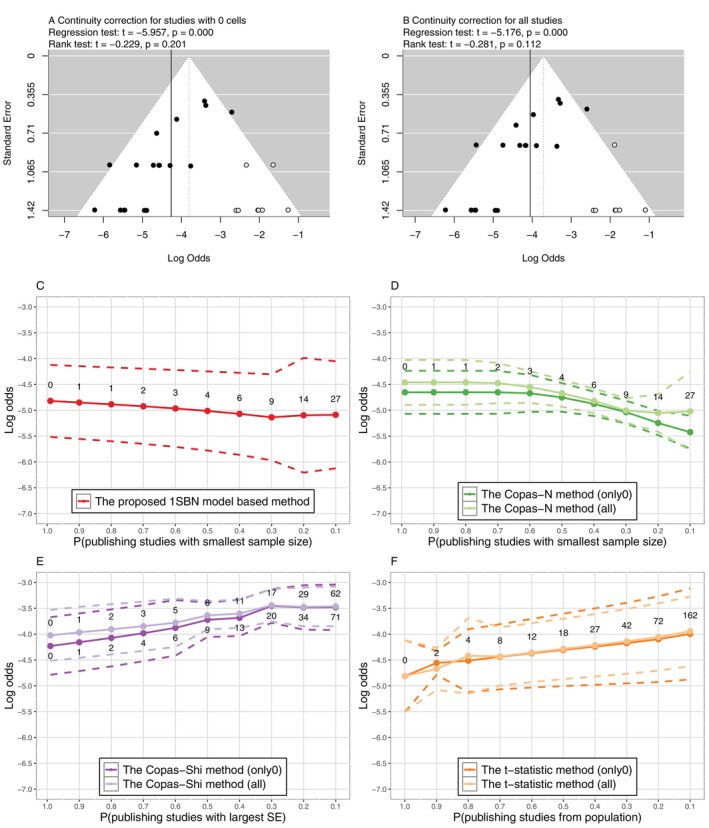
Comparison of sensitivity analysis methods for PB in Example 3. The values indicate numbers of missing studies. Methods in panels (A), (B), (D), (E), and (F) need continuity correction, and panel (C) does not; only0 indicates continuity correction for studies with 0 cells only, while all indicates continuity correction for all studies. The dashed lines indicate 95% CI.

To evaluate the impact of PB on the log odds of CRBSI, we employed the proposed method and compared the results with those of the Copas‐Heckman‐type methods and the t‐statistic method. We adopted the same scenarios on the sensitivity parameters, $\left(P_{\min},P_{\max}\right)$ and p, as described in Section 6.1, to re‐estimate the parameters of interest. The sensitivity analysis results by various methods were presented in Figure 3.

The funnel plot with Egger's test revealed significant asymmetry of the funnel plot, implying the existence of PB. Furthermore, the trim‐and‐fill method indicated that eight and seven studies were potentially unpublished under two approaches of continuity correction, respectively. The log odds were estimated to be increased when accounting for the unpublished studies. By the proposed BN method, as the number of unpublished studies increased, the estimated overall log odds decreased (Figure 3C), indicating that ignoring selective publication of studies might induce overestimation of the meta‐analysis results. Although the impact of PB was not significant, the estimated log odds of infections would be smaller when taking PB into consideration. The Copas‐N method also estimated a decreasing log odds with increasing number of unpublished studies, where the estimates decreased abruptly when $P_{\min}$ was less than 0.6 (Figure 3D). In contrast, the Copas‐Shi method and the t‐statistic method yielded increasing estimations of the log odds (Figure 3E,F) when assuming that PB was related to the SEs and the p value of the log odds. Among the two methods, the Copas‐Shi method had apparent overestimations (Figure 3E).

Although the signs of PB were estimated differently, the impact of PB analyzed by all the sensitivity analyses were not significant, the detailed estimations were summarized in Table C22–C25 of Supporting Information. The results indicated that the overall log odds of CRBSI was subtlety influenced by PB. Thus, a robust conclusion from the meta‐analysis could be drawn.

### 6.4 Example 4: Rare‐Event Meta‐Analysis of Proportions

This meta‐analysis originally aimed to estimate the overall success rate of hyperdynamic therapy for treating cerebral vasospasm and included 14 studies on the effectiveness of hyperdynamic therapy [39]. To provide a more intuitive explanation, we define the event of interest to be the not improved, and then two studies had 0 events, and six studies had fewer than three events. In this example, lower log odds was considered preferable, indicating the lower probability of non‐improvement. The data of this example were presented in Table C26 of Supporting Information. In this case, the parameter of interest was the log odds of the overall failure rate of hyperdynamic therapy. Without considering PB, the log odds with 95% CI was estimated to be −1.377(−1.942,−0.811) by the 1SBN model and −1.118(−1.598,−0.637) by the NN model.

**FIGURE 4:**
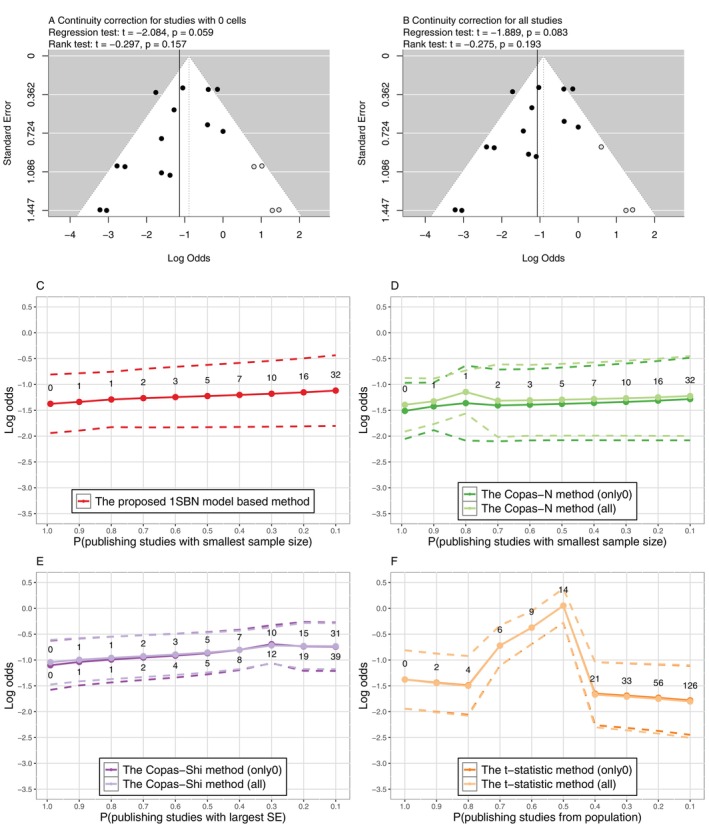
Comparison of sensitivity analysis methods for PB in Example 4. The values indicate numbers of missing studies. Methods in panels (A), (B), (D), (E), and (F) need continuity correction, and panel (C) does not; only0 indicates continuity correction for studies with 0 cells only, while all indicates continuity correction for all studies. The dashed lines indicate 95% CI.

The same methods were implemented to assess the impact of PB on the results, as shown in Figure 4. Under two approaches of continuity correction, Egger's regression test did not detect a significant existence of PB in the funnel plots, and the trim‐and‐fill methods indicated four and three studies, respectively. With the filled methods, the larger estimations on the log odds were adjusted (Figure 4A,B). The sensitivity analysis methods presented the adjusted estimates of the log odds given various numbers of unpublished studies. The proposed method and the Copas‐Shi and Copas‐N methods derived increasing estimates of the log odds while the t‐statistic method showed the decreasing estimate, when the number of unpublished studies increased (Figure 4C–F). Under the proposed method, accounting for PB, the estimated the log odds of the non‐improvement would be greater. This suggested that the meta‐analysis without accounting for PB might overestimate the treatment effect. However, the Copas‐N method seemed to underestimate the overall log odds while the Copas‐Shi method overestimated it (Figure 4D,E). The t‐statistic method required large amount of time and derived some unstable estimations (Figure 4F). Except for the proposed method, the other three methods were affected to some extent by different approaches to continuity correction. The detailed estimations were summarized in Table C27–C30 of Supporting Information. The proposed sensitivity analysis method provided increased confidence that the overall log odds of the failure rate estimated by the 1SBN model is robust to PB in this example.

## 7 Discussion

Increasing the estimation accuracy for rare‐event meta‐analysis has been extensively studied over the years. Various models employing the exact likelihoods of events have been proposed to increase the estimation accuracy, including beta‐binomial models [8, 10, 40] and Poisson random‐effects model [7]. Among these, the contrast‐based HN and BN models appear to have relatively broad applications, since they have been implemented in widely used R packages for meta‐analyses [9], such as metafor [41] and meta [42]. In systematic reviews and meta‐analyses, PB has been recognized as an inevitable issue. According to the Preferred Reporting Items for Systematic reviews and Meta‐Analyses (PRISMA) guideline [43], it is recommended to conduct sensitivity analyses to assess the robustness of the synthesized results (item 13f), and to present assessments of bias due to missing results (item 21). To deal with PB, many advanced selection‐model‐based methods have been proposed [15, 16, 17, 22, 23, 24, 25]. However, implementing these methods for rare‐event meta‐analyses can be challenging and problematic since they are developed based on the NN model.

The proposed methods serve as useful tools for addressing PB and assessing the robustness of results synthesized by the HN and BN models, overcoming the lack of sensitivity analysis methods for addressing PB in rare‐event meta‐analyses. Methods dealing with PB in the NN model have been extensively studies; in contrast, to the best of our knowledge, only one method [30] is available for addressing PB in rare‐event meta‐analysis, and it employed selection function on the t‐statistic of the lnOR. However, the continuity correction was still required for calculating the t‐statistics, and the Heckman‐type selection function utilized in this paper cannot be handled. Our proposal employed a different selection function based on the study sample sizes and is much simpler in the estimation. The computation times summarized for the examples further demonstrated that out proposal was considerably more time‐consuming (Table C28 of Supporting Information), especially with a larger number of subjects and studies (e.g., Example 2). The proposed method is not intended to replace the t‐statistic selection function‐based sensitivity analysis method [30]. Instead, we regard the proposed methods as part of sensitivity analysis for rare‐event meta‐analysis of ORs and proportions. In practice, since the explicit selection mechanism behind PB is unknown, it is recommended to conduct sensitivity analyses with different selection functions to evaluate PB and draw robust conclusions.

The proposed methods employed the selection function on the sample sizes of studies, which was adopted by Copas to address PB in the standard meta‐analysis [15]. Recently, this selection function was also used to deal with PB in meta‐analysis of diagnostic studies [26]. In the Copas‐Heckman‐type sensitivity analyses, the normal residuals in the NN model and the Gaussian latent variable were linked to address PB at the level of observed outcomes. Instead, in the proposed methods, the normally distributed study‐specific true effect size was directly associated with normal residual in the Gaussian latent variable to address PB at the level of the study‐specific random effects. Simulation studies have shown that the proposed methods, especially the HN model‐based sensitivity analysis method, obtained satisfactory performance in adjusting PB in the meta‐analytical estimates.

The proposed methods are advantageous in their applicability, as they can be applied to any rare‐event meta‐analyses that can be synthesized using the HN or BN models, and can be easily implemented by free statistical software, such as R. The proposed methods handle PB in rare‐event meta‐analyses of different outcomes, and can be extended into similar models, such as meta‐analysis of incidence rate ratio using the BN model [4]. We regard the proposed methods as supplemental analyses aiming at evaluating PB in rare‐event meta‐analysis. For rare‐event meta‐analysis of ORs, we suggest using the HN model for meta‐analysis and the proposed HN model‐based sensitivity analysis for PB evaluation. Although the HN‐based sensitivity analysis method involves some computational complexity, the computation time is typically within a few minutes or less when the number of studies is moderate. The proposed CBN‐based method is recommended only when the number of studies and subjects is sufficiently large, as it can help reduce computation time in such cases. The HN‐based or CBN‐based models are also regarded as the contrast‐based GLMMs, which have been more studied and applied in the network meta‐analysis [12]. We expect the proposed framework of sensitivity analysis to be extended into the network meta‐analysis setting in future work.

In rare‐event meta‐analysis of ORs, the arm‐based GLMMs are also useful (e.g., the bivariate BN model 2–6 in Jackson et al. [9]), especially when the baseline odds (i.e., random study intercepts) are of interest. Due to the feature of the arm‐based design, additional missing data issue could arise. In specific, the probability of a particular design being chosen may depend on the results for that treatment, which consequently leads to the risk of systematically missing arms in the studies [4, 12]. Thus, the sensitivity analysis on the bivariate BN models would be more complicated and need to consider additional sources of potential bias. From a modeling perspective, the proposed methods could be extended to the unconditional bivariate BN models to deal with PB only. Hattori and Zhou [26] employed the similar selection function (13) to address PB in meta‐analysis of diagnostic studies. Using a similar bivariate BN model, they considered the feature of diagnostic studies and introduced the latent biomarker model to link the selection function for adjusting PB. Their work suggests a direction for methodological extensions for the arm‐based GLMMs. However, an appropriate latent model should be introduce to define a selective publication, and stable estimation procedures to handle the complexity of the likelihood under bivariate BN models (e.g., involving double integrals and potential overparameterization, such as Equation 12 in Hattori and Zhou [26]). The development of such methods will be pursued in future research.

One limitation of this proposal is that the adopted Copas‐Heckman‐type selection function is unable to describe selective publication process in a bi‐directional nature. Specifically, studies reporting either relatively high or low event outcomes may be equally likely to be published. The Copas‐Heckman‐type selection function cannot model this kind of publication process well since the relationship between the outcome and the latent variable for publication is defined by a single parameter ρ (see explanations in Equation 14). Such case may happen particularly in rare‐event meta‐analyses of proportions, as illustrated in Example 3 (Section 6.3), where both high and low infection odds may be of interest. Further methodological development may be required in future work.

Recently, several studies have also indicated that the beta‐binomial model exhibits better performance in rare‐event meta‐analyses [8, 10, 40]. However, extending the proposed framework into this model is not straightforward and will be considered in future work. In some cases, rare events coincide with meta‐analyses that comprise only a few studies. Our simulation studies demonstrated that the proposed methods worked in meta‐analysis with about 10 published studies, with less bias in the HN model‐based method. If the number of studies is too small, the proposed methods may suffer from convergence issues and increased estimation bias. Despite these limitations, our proposal is still valuable and provides a straightforward and practical approach to assessing PB in rare‐event meta‐analyses. Several simulation studies have reported that the HN model could yield satisfactory performance and was recommended for practical use [5, 9]. At the present stage, we consider the proposed methods to be useful contributions to addressing PB within the GLMM framework.

## Funding

This work was supported by Grant‐in‐Aid for Scientific Research (25K03086, 26K21181) from the Ministry of Education, Science, Sports and Technology in Japan.

## Conflicts of Interest

The authors declare no conflicts of interest.

## Supporting information


**Appendix S1:** sim70595‐sup‐0001‐AppendixS1.pdf.

## Data Availability

R codes used in this paper, together with a sample application data set, are available at https://github.com/meta2020/remetasa‐r.
