# Supplementary material for: Copas‐Heckman‐Type Sensitivity Analysis for Publication Bias in Rare‐Event Meta‐Analysis Under Generalized Linear Mixed Models
Source: Stat Med. 2026 May 12;45:e70595. doi: 10.1002/sim.70595 (PMC13162210; doi:10.1002/sim.70595)
Supplement: Supplementary file 1 — Appendix S1: sim70595‐sup‐0001‐AppendixS1.pdf. [file SIM-45-0-s001.pdf]

Received: Added at production | Revised: Added at production | Accepted: Added at production
DOI: xxx/xxxx

SUPPLEMENTARY MATERIAL

# Appendix for "Copas-Heckman-type sensitivity analysis for publication bias in rare-event meta-analysis under generalized linear mixed models"

**Yi Zhou[1,2,3] | Taojun Hu[3,4] | Ao Huang[5] | Yuji Sakamoto[1] | Xiao-Hua Zhou*[2,4] | Satoshi Hattori*[3,6]**

[1]Division of Mathematics and Informatics, Graduate School of Human Development and Environment, Kobe University, Kobe, Japan

[2]Beijing International Center for Mathematical Research, Peking University, Beijing, China

[3]Department of Biomedical Statistics, Graduate School of Medicine, The University of Osaka, Osaka, Japan

[4]Department of Biostatistics, Peking University, Beijing, China

[5]Department of Medical Statistics, University Medical Center Göttingen, Göttingen, Germany

[6]Integrated Frontier Research for Medical Science Division, Institute for Open and Transdisciplinary Research Initiatives, The University of Osaka, Osaka, Japan

## APPENDIX

 © Copyright Holder Name 

# A REVIEW OF THE COPAS-N METHOD FOR PUBLICATION BIAS IN THE NORMAL-NORMAL RANDOM EFFECTS MODEL

As mentioned in Section 2.2 of the main text, we review the Copas-N sensitivity analysis method[1].

Copas[1] extended the Heckman-type selection function and proposed a selection-model-based method to address PB on associations between treatment and the continuous outcomes. For binary data, the outcomes can be the log-transformed odds ratios (ORs) or the log-transformed odds. Then, the outcomes were re-expressed by the following random-effects model:

$$\hat{\theta}_i = \theta + (n_i^{-1} + \tau^2)^{1/2} \epsilon_i, \tag{A1}$$

where $\epsilon_i$ is the random residual. However, the likelihood (A1) is not exactly same with the NN model. To address PB, Copas[1] introduced a latent Gaussian variable $Z_i$ with the selection equation defined as:

$$Z_i = \gamma_0 + \gamma_1 n_i^{1/2} + \delta_i.$$

In the above equations, $\epsilon_i$ and $\delta_i$ were regarded as random residuals with the following distribution:

$$\begin{pmatrix} \epsilon_i \\ \delta_i \end{pmatrix} \sim N\left( \begin{pmatrix} 0 \\ 0 \end{pmatrix}, \begin{bmatrix} 1 & \rho \\ \rho & 1 \end{bmatrix} \right),$$

where $\gamma_0$ and $\gamma_1$ are constants and $\rho$ indicates the correlation between $\epsilon_i$ and $\delta_i$. Under selective publication of studies, the log-likelihood conditional on the published studies ($Z_i > 0$) was derived in equation (7) of Copas,[1] shown as follows:

$$\ell(\theta, \tau, \rho, \gamma_0, \gamma_1) = \sum_{i=1}^{N} \left\{ -\frac{1}{2} \log\left(\tau^2 + \frac{1}{n_i}\right) - \frac{(y-\theta)^2}{2(\tau^2 + n_i^{-1})} - \log \Phi(\gamma_0 + \gamma_1 n_i^{1/2}) + \log \Phi(v_i) \right\} \tag{A2}$$

with

$$v_i = \frac{\gamma_0 + \gamma_1 n_i^{1/2} + \rho(\hat{\theta}_i - \theta)/\sqrt{\tau^2 + n_i^{-1}}}{\sqrt{1-\rho^2}}$$

Given fixed pairs of values for $(\gamma_0, \gamma_1)$, the rest parameters can be estimated by the maximum likelihood estimation.

# B ADDITIONAL INFORMATION IN SIMULATION STUDIES

To examine the performance of various model under the misspecified selection processes, we considered selecting studies based on their $P$-values using the Hedges-type selection models, where studies with large $P$-values had more probability to be selected.

## B.1 Scenarios of the Hedges-type selection model

### B.1.1 Meta-analysis of odds ratios

For studies generated by the HN model, we considered the following Hedges-type selection model to select the published studies.

$$w_1(p_i) = \begin{cases} 0.9, & \text{if } \quad p_i < 0.01 \\ 0.8, & \text{if } 0.01 \leq p_i < 0.03 \\ 0.7, & \text{if } 0.03 \leq p_i < 0.05 \\ 0.4, & \text{if } \quad p_i \geq 0.05 \end{cases}, \tag{B3}$$

For studies generated by the 2SBN model, we observed more occurrences of 0 events in both arms, leading to the more frequencies of large $P$-values. Thus, we considered the following Hedges-type selection model to select studies.

$$w_2(p_i) = \begin{cases} 0.9, & \text{if } \quad p_i < 0.05 \\ 0.8, & \text{if } 0.05 \leq p_i < 0.1 \\ 0.7, & \text{if } 0.1 \leq p_i < 0.2 \\ 0.4, & \text{if } \quad p_i \geq 0.2 \end{cases}, \tag{B4}$$

where $w_j(p_i)$, $(j = 1, 2)$ denotes the publication probabilities of the $i$th study, and $p_i$ is the estimated $p$-value of the log-transformed odds ratios (lnOR). Then, the random variable $Z_i$ was generated by Bernoulli distribution, $Ber(w(p_i))$. Studies were selected as published ones if $Z_i = 1$; otherwise, they were treated as unpublished.

### B.1.2 Meta-analysis of proportions

For meta-analysis of proportions, since most of the manipulated studies low $p$-values, we considered the following Hedges-type selection model.

$$w_3(p_i) = \begin{cases} 0.9, & \text{if } \quad p_i < 0.001 \\ 0.8, & \text{if } 0.001 \leq p_i < 0.005 \\ 0.7, & \text{if } 0.005 \leq p_i < 0.01 \\ 0.6, & \text{if } 0.01 \leq p_i < 0.05 \\ 0.3, & \text{if } \quad p_i \geq 0.05 \end{cases}, \tag{B5}$$

where $w_3(p_i)$ denotes the publication probabilities of the $i$th study, and $p_i$ is the estimated $p$-value of the log-transformed odds ratios (lnOR). Then, studies were selected using the similar process as above.

## B.2 Implementations

As mentioned in Section 5.2 of the main text, we describe the detailed implementation of simulation studies. Simulation studies were conducted by R (version 4.0.3) on system of Intel Xeon Platinum 8368. The random numbers from hypergeometric distribution (equation 7 in the main text) were generated by the R function `rFNCHypergeo()` in the R package `BiasedUrn`.[3,4] The random numbers from binomial distribution were generated by `rbinom()` in the R package `stats`. The maximum likelihood estimations were optimized by the numerical Newton-Raphson method and implemented by `nlminb()` in the R package `stats` with initial values set as true parameters plus the random values from uniform distribution $U[-0.1, 0.1]$ or $U[-0.2, 0.2]$. The settings of initial value might be arbitrary. The also implemented different initial values by adopting the estimated $\hat{\theta}$ from the

normal-normal (NN) models as initial values for $\theta$ and 0.5 for $\tau$ for estimation; however, the estimates had only small changes and the conclusions did not change.

When implementing the method of Hu et al.,[2] the sensitivity parameter, the overall publication probability $p$, was set as the proportion of the published studies among population studies. For simulation studies under the Hedges-type selection model, the true value of $(P_{\min}, P_{\max})$ are unknown; thus, we set the minimum and maximum values of $w(p_i)$ in the selected (published) studies as $(P_{\min}, P_{\max})$ in the proposed method.

The integrations in the hypergeometric-normal (HN) and binomial-normal (BN) models were implemented numerically by `hcubature()` in the R package `cubature`.[5]

## B.3 Summary of the amount of rare events

As mentioned in Section 5.2 of the main text, we summarized the proportion of studies with fewer than three events in simulation studies. Corresponding to different data-generating processes and scenarios of parameters, for meta-analysis of ORs, the proportions of studies with rare events in treatment and control group were summarized and presented in Table B1. For meta-analysis of proportions, the proportions of studies with rare events were summarized and presented in Table B2. The subscripts $P$ and $O$ indicates the proportions of rare events in the population studies (published and unpublished studies) and the published (observed) studies, respectively.

## B.4 Additional result 1: meta-analysis of odds ratios under the HN model based data-generating process

As mentioned in Sections 5.2, we presented convergence rates (Table B3) and average estimates of $\tau^2$ (Table B4) when population data were simulated under the HN model based data-generating process (B3). The results under the Hedges-type selection model were presented in Table B5.

## B.5 Additional result 2: meta-analysis of odds ratios under the 2SBN model based data-generating process

As mentioned in Sections 5.3, we presented convergence rates (Table B6) and average estimates of $\tau^2$ (Table B7) when population data were simulated under the real-world data-generating process (B4). The results under the Hedges-type selection model were presented in Table B8.

## B.6 Additional result 3: meta-analysis of proportions under the 1SBN model data-generating process

As mentioned in Sections 5.4, we presented convergence rates (Table B9) and average estimates of $\tau^2$ (Table B10) when population data were simulated under the SGBN model based data-generating process (B5). The results under the Hedges-type selection model were presented in Table B11.

**TABLE B1** Summary of the proportions of studies with rare events under the HN and 2SBN model based data-generating processes.

| S | Patients | T:C | $\tau^2$ | $HN_P$ | $HN_O$ | $2SBN_P$ | $2SBN_O$ |
|---|---|---|---|---|---|---|---|
| 15 | U[30, 60] | 1:1 | 0.1 | 89.1 | 88.7 | 99.6 | 99.4 |
| | | | 0.3 | 87.3 | 85.8 | 99.2 | 98.9 |
| | | | 0.7 | 85.4 | 82.7 | 97.9 | 97.1 |
| | | 2:1 | 0.1 | 61.9 | 62.0 | 99.3 | 99.1 |
| | | | 0.3 | 61.3 | 60.2 | 98.9 | 98.5 |
| | | | 0.7 | 63.0 | 61.2 | 97.7 | 96.9 |
| | U[50, 200] | 1:1 | 0.1 | 94.4 | 94.0 | 90.9 | 87.9 |
| | | | 0.3 | 91.8 | 90.5 | 88.5 | 84.6 |
| | | | 0.7 | 88.2 | 85.5 | 84.3 | 79.2 |
| | | 2:1 | 0.1 | 75.5 | 74.7 | 85.0 | 80.4 |
| | | | 0.3 | 74.7 | 72.5 | 83.3 | 78.2 |
| | | | 0.7 | 74.6 | 71.6 | 80.4 | 74.2 |
| | U[500, 700] | 1:1 | 0.1 | 95.6 | 94.6 | 36.9 | 32.9 |
| | | | 0.3 | 93.6 | 91.7 | 39.3 | 34.0 |
| | | | 0.7 | 90.4 | 87.5 | 42.9 | 36.2 |
| | | 2:1 | 0.1 | 82.4 | 80.2 | 28.9 | 26.2 |
| | | | 0.3 | 79.6 | 76.0 | 32.5 | 28.3 |
| | | | 0.7 | 78.3 | 73.7 | 36.8 | 30.8 |
| 50 | U[30, 60] | 1:1 | 0.1 | 89.4 | 88.9 | 99.7 | 99.6 |
| | | | 0.3 | 87.8 | 86.1 | 99.2 | 98.9 |
| | | | 0.7 | 85.2 | 82.3 | 97.9 | 97.1 |
| | | 2:1 | 0.1 | 61.4 | 61.2 | 99.5 | 99.3 |
| | | | 0.3 | 62.3 | 61.2 | 98.9 | 98.5 |
| | | | 0.7 | 63.7 | 61.6 | 97.6 | 96.7 |
| | U[50, 200] | 1:1 | 0.1 | 94.2 | 93.5 | 91.7 | 89.2 |
| | | | 0.3 | 92.0 | 90.5 | 89.3 | 85.9 |
| | | | 0.7 | 89.2 | 87.0 | 86.3 | 82.1 |
| | | 2:1 | 0.1 | 75.9 | 74.9 | 86.6 | 82.9 |
| | | | 0.3 | 74.8 | 72.4 | 84.8 | 80.3 |
| | | | 0.7 | 74.6 | 71.3 | 82.6 | 77.5 |
| | U[500, 700] | 1:1 | 0.1 | 95.7 | 94.9 | 42.8 | 39.1 |
| | | | 0.3 | 93.7 | 92.2 | 46.0 | 40.9 |
| | | | 0.7 | 90.6 | 88.0 | 48.8 | 42.7 |
| | | 2:1 | 0.1 | 81.8 | 79.5 | 36.0 | 33.2 |
| | | | 0.3 | 80.1 | 76.6 | 39.3 | 35.1 |
| | | | 0.7 | 78.7 | 74.3 | 43.1 | 37.9 |

Results of $HN_P$ and $2SBN_P$ are based on the population studies; results of $HN_O$ and $2SBN_O$ are based on the published studies.

**TABLE B2** Summary of the proportions of studies with rare events under the 1SBN model based data-generating processes.

| S | Patients | $\tau^2$ | $1SBN_P$ | $1SBN_O$ |
|---|---|---|---|---|
| 15 | U[15, 30] | 0.1 | 91.9 | 89.0 |
| | | 0.3 | 87.2 | 82.4 |
| | | 0.7 | 81.6 | 75.1 |
| | U[25, 100] | 0.1 | 34.9 | 20.1 |
| | | 0.3 | 37.7 | 22.1 |
| | | 0.7 | 40.4 | 25.0 |
| 50 | U[15, 30] | 0.1 | 91.9 | 89.3 |
| | | 0.3 | 87.6 | 83.2 |
| | | 0.7 | 81.6 | 75.3 |
| | U[25, 100] | 0.1 | 34.7 | 20.9 |
| | | 0.3 | 37.5 | 22.8 |
| | | 0.7 | 40.1 | 25.6 |

Results of $1SBN_P$ are based on the population studies; results of $1SBN_O$ are based on the published studies.

**TABLE B3** Convergence proportion (%) of estimations among different models under the HN model based data-generating process.

| $S$ | Patients | T:C | $\tau^2$ | $N$ | $NN_P$ | $NN_O$ | CN | CS | $HN_P$ | $HN_O$ | $HN^{Prop}$ | $HN^{Hu}$ | $CBN_P$ | $CBN_O$ | $CBN^{Prop}$ | $CBN^{Hu}$ |
|---|---|---|---|---|---|---|---|---|---|---|---|---|---|---|---|---|
| 15 | U[30, 60] | 1:1 | 0.1 | 10.6 | 100.0 | 100.0 | 100 | 100.0 | 71.8 | 67.8 | 62.1 | 100.0 | 63.4 | 61.2 | 67.1 | 79.5 |
| | | | 0.3 | 10.6 | 99.9 | 99.8 | 100 | 100.0 | 82.1 | 75.6 | 76.2 | 99.9 | 71.8 | 71.8 | 78.2 | 75.3 |
| | | | 0.7 | 10.6 | 99.6 | 99.8 | 100 | 100.0 | 92.4 | 88.7 | 89.6 | 99.9 | 85.5 | 80.3 | 87.0 | 75.6 |
| | | 2:1 | 0.1 | 10.6 | 100.0 | 100.0 | 100 | 99.9 | 76.5 | 70.7 | 63.2 | 99.9 | 55.3 | 59.0 | 64.2 | 74.3 |
| | | | 0.3 | 10.6 | 100.0 | 99.8 | 100 | 100.0 | 84.2 | 81.1 | 81.2 | 100.0 | 68.6 | 68.2 | 78.9 | 75.1 |
| | | | 0.7 | 10.5 | 99.4 | 99.5 | 100 | 100.0 | 95.0 | 90.7 | 89.7 | 100.0 | 84.3 | 83.1 | 87.1 | 75.8 |
| | U[50, 200] | 1:1 | 0.1 | 10.8 | 99.9 | 100.0 | 100 | 100.0 | 72.1 | 67.8 | 65.3 | 100.0 | 66.1 | 66.4 | 71.0 | 79.2 |
| | | | 0.3 | 10.8 | 99.7 | 99.6 | 100 | 100.0 | 81.3 | 77.0 | 74.0 | 100.0 | 79.0 | 77.1 | 79.7 | 75.6 |
| | | | 0.7 | 10.9 | 99.0 | 99.3 | 100 | 100.0 | 92.1 | 88.2 | 88.1 | 99.8 | 90.2 | 86.8 | 91.1 | 74.1 |
| | | 2:1 | 0.1 | 10.9 | 100.0 | 100.0 | 100 | 100.0 | 74.9 | 70.9 | 68.0 | 100.0 | 70.8 | 68.1 | 75.1 | 77.5 |
| | | | 0.3 | 10.8 | 99.7 | 99.9 | 100 | 100.0 | 88.4 | 82.2 | 81.8 | 99.9 | 82.2 | 78.9 | 82.5 | 74.4 |
| | | | 0.7 | 10.8 | 99.5 | 99.4 | 100 | 100.0 | 96.0 | 92.6 | 91.0 | 99.9 | 94.6 | 90.6 | 93.2 | 75.6 |
| | U[500, 700] | 1:1 | 0.1 | 10.4 | 99.9 | 99.8 | 100 | 100.0 | 72.7 | 71.8 | 66.2 | 100.0 | 71.7 | 69.2 | 67.9 | 72.7 |
| | | | 0.3 | 10.4 | 100.0 | 99.7 | 100 | 100.0 | 79.1 | 74.2 | 75.8 | 99.9 | 80.2 | 75.2 | 77.4 | 71.8 |
| | | | 0.7 | 10.4 | 99.2 | 99.8 | 100 | 100.0 | 92.7 | 86.9 | 86.5 | 100.0 | 92.0 | 88.0 | 90.5 | 71.2 |
| | | 2:1 | 0.1 | 10.4 | 99.9 | 99.9 | 100 | 100.0 | 73.5 | 72.5 | 67.3 | 99.9 | 73.0 | 72.9 | 72.7 | 71.5 |
| | | | 0.3 | 10.5 | 99.8 | 99.8 | 100 | 100.0 | 85.7 | 81.6 | 81.0 | 100.0 | 87.0 | 81.3 | 83.6 | 72.9 |
| | | | 0.7 | 10.4 | 99.7 | 99.6 | 100 | 100.0 | 95.1 | 92.0 | 91.6 | 100.0 | 95.9 | 91.2 | 94.3 | 70.6 |
| 50 | U[30, 60] | 1:1 | 0.1 | 35.9 | 99.9 | 99.5 | 100 | 100.0 | 80.4 | 77.1 | 75.1 | 99.8 | 62.2 | 65.6 | 74.7 | 95.7 |
| | | | 0.3 | 36.0 | 99.7 | 100.0 | 100 | 100.0 | 93.6 | 88.4 | 90.8 | 99.9 | 81.4 | 78.2 | 86.1 | 95.0 |
| | | | 0.7 | 36.1 | 99.8 | 99.7 | 100 | 100.0 | 99.2 | 97.7 | 97.8 | 100.0 | 96.6 | 94.4 | 97.5 | 93.5 |
| | | 2:1 | 0.1 | 35.8 | 99.7 | 99.8 | 100 | 100.0 | 83.2 | 78.4 | 79.4 | 99.9 | 55.9 | 59.0 | 72.1 | 93.2 |
| | | | 0.3 | 36.0 | 99.7 | 99.8 | 100 | 100.0 | 95.8 | 92.7 | 94.0 | 99.8 | 76.1 | 77.7 | 86.2 | 94.5 |
| | | | 0.7 | 35.9 | 99.5 | 100.0 | 100 | 99.9 | 99.8 | 99.3 | 99.2 | 100.0 | 97.8 | 95.5 | 98.1 | 93.3 |
| | U[50, 200] | 1:1 | 0.1 | 36.9 | 99.9 | 99.4 | 100 | 100.0 | 80.0 | 75.9 | 78.9 | 99.5 | 73.7 | 73.6 | 83.1 | 90.5 |
| | | | 0.3 | 36.9 | 99.8 | 99.6 | 100 | 100.0 | 91.8 | 89.3 | 90.7 | 99.7 | 91.7 | 89.5 | 92.9 | 90.7 |
| | | | 0.7 | 36.9 | 99.9 | 99.6 | 100 | 100.0 | 99.5 | 98.8 | 98.0 | 100.0 | 99.2 | 98.5 | 99.2 | 92.0 |
| | | 2:1 | 0.1 | 37.0 | 99.9 | 100.0 | 100 | 100.0 | 84.6 | 81.4 | 79.9 | 99.9 | 76.4 | 77.6 | 85.7 | 92.3 |
| | | | 0.3 | 37.0 | 99.9 | 99.8 | 100 | 100.0 | 97.8 | 94.9 | 94.8 | 100.0 | 94.4 | 92.8 | 96.3 | 92.2 |
| | | | 0.7 | 37.0 | 99.9 | 100.0 | 100 | 100.0 | 100.0 | 99.8 | 99.1 | 100.0 | 99.9 | 99.6 | 99.5 | 91.2 |
| | U[500, 700] | 1:1 | 0.1 | 35.3 | 99.0 | 99.3 | 100 | 100.0 | 78.4 | 77.0 | 75.4 | 99.8 | 78.4 | 75.3 | 77.5 | 92.5 |
| | | | 0.3 | 35.3 | 99.3 | 99.7 | 100 | 100.0 | 92.6 | 89.6 | 90.2 | 99.9 | 91.4 | 87.8 | 90.7 | 92.7 |
| | | | 0.7 | 35.3 | 99.9 | 98.8 | 100 | 100.0 | 99.5 | 97.7 | 97.9 | 100.0 | 99.1 | 97.2 | 98.4 | 93.5 |
| | | 2:1 | 0.1 | 35.4 | 99.7 | 100.0 | 100 | 100.0 | 82.4 | 79.5 | 80.0 | 99.9 | 82.3 | 79.2 | 80.6 | 93.9 |
| | | | 0.3 | 35.4 | 99.6 | 99.8 | 100 | 100.0 | 97.0 | 93.7 | 94.4 | 99.9 | 97.8 | 93.4 | 95.8 | 92.5 |
| | | | 0.7 | 35.3 | 100.0 | 100.0 | 100 | 100.0 | 99.8 | 99.4 | 99.2 | 100.0 | 99.8 | 99.6 | 99.6 | 92.0 |

Results of $NN_P$, $HN_P$, and $CBN_P$ are based on the population studies; results of $NN_O$, $HN_O$, and $CBN_O$ are based on the published studies; CN and CS indicate the Copas-N and Copas-Shi methods; $HN^{Prop}$ and $CBN^{Prop}$ indicate the proposed HN or CBN model based sensitivity analysis methods. $HN^{Hu}$ and $CBN^{Hu}$ indicate the $t$-statistic based sensitivity analysis methods.

**TABLE B4** Averages of estimates of $\tau^2$ among different models under the HN model based data-generating process.

| $S$ | Patients | T:C | $\tau^2$ | $N$ | $NN_P$ | $NN_O$ | CN | CS | $HN_P$ | $HN_O$ | $HN^{Prop}$ | $HN^{Hu}$ | $CBN_P$ | $CBN_O$ | $CBN^{Prop}$ | $CBN^{Hu}$ |
|---|---|---|---|---|---|---|---|---|---|---|---|---|---|---|---|---|
| 15 | U[30, 60] | 1:1 | 0.1 | 10.6 | 0.04 | 0.05 | 0.81 | 0.00 | 0.23 | 0.27 | 0.40 | 0.18 | 0.12 | 0.17 | 0.26 | 0.10 |
| | | | 0.3 | 10.6 | 0.09 | 0.09 | 0.93 | 0.00 | 0.39 | 0.37 | 0.51 | 0.28 | 0.22 | 0.22 | 0.33 | 0.16 |
| | | | 0.7 | 10.6 | 0.22 | 0.20 | 1.20 | 0.01 | 0.67 | 0.64 | 0.81 | 0.57 | 0.39 | 0.43 | 0.55 | 0.34 |
| | | 2:1 | 0.1 | 10.6 | 0.06 | 0.06 | 0.85 | 0.01 | 0.18 | 0.19 | 0.27 | 0.13 | 0.06 | 0.08 | 0.15 | 0.05 |
| | | | 0.3 | 10.6 | 0.13 | 0.12 | 1.04 | 0.03 | 0.33 | 0.31 | 0.42 | 0.25 | 0.13 | 0.16 | 0.22 | 0.10 |
| | | | 0.7 | 10.5 | 0.34 | 0.30 | 1.40 | 0.09 | 0.68 | 0.61 | 0.77 | 0.56 | 0.29 | 0.32 | 0.42 | 0.27 |
| | U[50, 200] | 1:1 | 0.1 | 10.8 | 0.03 | 0.03 | 0.70 | 0.00 | 0.25 | 0.27 | 0.38 | 0.18 | 0.21 | 0.23 | 0.33 | 0.15 |
| | | | 0.3 | 10.8 | 0.07 | 0.07 | 0.83 | 0.00 | 0.43 | 0.42 | 0.60 | 0.32 | 0.35 | 0.36 | 0.51 | 0.27 |
| | | | 0.7 | 10.9 | 0.17 | 0.15 | 1.05 | 0.01 | 0.72 | 0.64 | 0.82 | 0.57 | 0.61 | 0.57 | 0.72 | 0.50 |
| | | 2:1 | 0.1 | 10.9 | 0.03 | 0.03 | 0.76 | 0.00 | 0.17 | 0.18 | 0.27 | 0.13 | 0.12 | 0.15 | 0.21 | 0.10 |
| | | | 0.3 | 10.8 | 0.08 | 0.08 | 0.93 | 0.01 | 0.31 | 0.32 | 0.44 | 0.26 | 0.24 | 0.27 | 0.37 | 0.21 |
| | | | 0.7 | 10.8 | 0.26 | 0.21 | 1.30 | 0.05 | 0.70 | 0.64 | 0.79 | 0.59 | 0.55 | 0.54 | 0.66 | 0.47 |
| | U[500, 700] | 1:1 | 0.1 | 10.4 | 0.02 | 0.03 | 0.66 | 0.00 | 0.25 | 0.28 | 0.41 | 0.20 | 0.24 | 0.28 | 0.39 | 0.18 |
| | | | 0.3 | 10.4 | 0.06 | 0.06 | 0.77 | 0.00 | 0.40 | 0.39 | 0.53 | 0.29 | 0.38 | 0.38 | 0.51 | 0.27 |
| | | | 0.7 | 10.4 | 0.16 | 0.16 | 1.03 | 0.01 | 0.77 | 0.73 | 0.92 | 0.63 | 0.75 | 0.70 | 0.87 | 0.60 |
| | | 2:1 | 0.1 | 10.4 | 0.03 | 0.03 | 0.76 | 0.00 | 0.19 | 0.21 | 0.31 | 0.15 | 0.18 | 0.20 | 0.29 | 0.15 |
| | | | 0.3 | 10.5 | 0.07 | 0.07 | 0.91 | 0.00 | 0.34 | 0.34 | 0.44 | 0.27 | 0.32 | 0.32 | 0.42 | 0.26 |
| | | | 0.7 | 10.4 | 0.21 | 0.18 | 1.22 | 0.02 | 0.71 | 0.64 | 0.79 | 0.59 | 0.67 | 0.62 | 0.74 | 0.55 |
| 50 | U[30, 60] | 1:1 | 0.1 | 35.9 | 0.02 | 0.02 | 0.82 | 0.00 | 0.16 | 0.16 | 0.21 | 0.12 | 0.07 | 0.08 | 0.14 | 0.05 |
| | | | 0.3 | 36.0 | 0.06 | 0.06 | 0.97 | 0.00 | 0.31 | 0.29 | 0.36 | 0.25 | 0.15 | 0.16 | 0.23 | 0.12 |
| | | | 0.7 | 36.1 | 0.25 | 0.20 | 1.28 | 0.00 | 0.69 | 0.59 | 0.71 | 0.58 | 0.37 | 0.34 | 0.43 | 0.33 |
| | | 2:1 | 0.1 | 35.8 | 0.03 | 0.03 | 0.88 | 0.00 | 0.14 | 0.14 | 0.18 | 0.11 | 0.03 | 0.04 | 0.09 | 0.02 |
| | | | 0.3 | 36.0 | 0.12 | 0.11 | 1.09 | 0.01 | 0.31 | 0.27 | 0.34 | 0.25 | 0.09 | 0.10 | 0.16 | 0.08 |
| | | | 0.7 | 35.9 | 0.37 | 0.31 | 1.50 | 0.06 | 0.68 | 0.59 | 0.70 | 0.59 | 0.25 | 0.26 | 0.34 | 0.25 |
| | U[50, 200] | 1:1 | 0.1 | 36.9 | 0.01 | 0.01 | 0.71 | 0.00 | 0.16 | 0.17 | 0.21 | 0.13 | 0.13 | 0.14 | 0.18 | 0.10 |
| | | | 0.3 | 36.9 | 0.04 | 0.04 | 0.84 | 0.00 | 0.33 | 0.30 | 0.37 | 0.27 | 0.26 | 0.25 | 0.32 | 0.22 |
| | | | 0.7 | 36.9 | 0.17 | 0.15 | 1.09 | 0.00 | 0.68 | 0.60 | 0.71 | 0.59 | 0.56 | 0.51 | 0.62 | 0.50 |
| | | 2:1 | 0.1 | 37.0 | 0.01 | 0.01 | 0.80 | 0.00 | 0.13 | 0.13 | 0.18 | 0.11 | 0.09 | 0.10 | 0.14 | 0.07 |
| | | | 0.3 | 37.0 | 0.07 | 0.06 | 0.99 | 0.00 | 0.30 | 0.27 | 0.33 | 0.26 | 0.22 | 0.21 | 0.27 | 0.20 |
| | | | 0.7 | 37.0 | 0.26 | 0.20 | 1.35 | 0.01 | 0.69 | 0.60 | 0.70 | 0.59 | 0.53 | 0.49 | 0.59 | 0.48 |
| | U[500, 700] | 1:1 | 0.1 | 35.3 | 0.01 | 0.01 | 0.68 | 0.00 | 0.16 | 0.17 | 0.23 | 0.13 | 0.16 | 0.16 | 0.21 | 0.12 |
| | | | 0.3 | 35.3 | 0.04 | 0.04 | 0.80 | 0.00 | 0.33 | 0.29 | 0.37 | 0.26 | 0.32 | 0.28 | 0.36 | 0.25 |
| | | | 0.7 | 35.3 | 0.15 | 0.14 | 1.04 | 0.00 | 0.68 | 0.59 | 0.70 | 0.58 | 0.66 | 0.58 | 0.68 | 0.56 |
| | | 2:1 | 0.1 | 35.4 | 0.01 | 0.01 | 0.77 | 0.00 | 0.14 | 0.13 | 0.18 | 0.11 | 0.13 | 0.12 | 0.17 | 0.10 |
| | | | 0.3 | 35.4 | 0.06 | 0.05 | 0.95 | 0.00 | 0.31 | 0.28 | 0.34 | 0.26 | 0.29 | 0.26 | 0.32 | 0.24 |
| | | | 0.7 | 35.3 | 0.21 | 0.18 | 1.30 | 0.00 | 0.70 | 0.60 | 0.70 | 0.60 | 0.67 | 0.58 | 0.68 | 0.58 |

Results of $NN_P$, $HN_P$, and $CBN_P$ are based on the population studies; results of $NN_O$, $HN_O$, and $CBN_O$ are based on the published studies; CN and CS indicate the Copas-N and Copas-Shi methods; $HN^{Prop}$ and $CBN^{Prop}$ indicate the proposed HN or CBN model based sensitivity analysis methods; $HN^{Hu}$ and $CBN^{Hu}$ indicate the $t$-statistic based sensitivity analysis methods.

**TABLE B5** Averages of estimation bias of the lnOR ($\theta = -2$) among different models under the HN model based data-generating process and the Hedges-type selection model (B3). All estimates were multiplied by 100.

| $S$ | Patients | T:C | $\tau^2$ | $N$ | $NN_P$ | $NN_O$ | CN (CP) | CS (CP) | $HN_P$ | $HN_O$ | $HN^{Prop}$ (CP) | $HN^{Hu}$ (CP) | $CBN_P$ | $CBN_O$ | $CBN^{Prop}$ (CP) | $CBN^{Hu}$ (CP) |
|---|---|---|---|---|---|---|---|---|---|---|---|---|---|---|---|---|
| 15 | U[30, 60] | 1:1 | 0.1 | 9.5 | 18.2 | -4.9 | -29.5 (67.5) | -49.2 (57.2) | -3.3 | -27.3 | -33.0 (90.0) | -24.3 (94.3) | 39.3 | 20.4 | 6.1 (84.4) | 23.1 (83.4) |
| | | | 0.3 | 9.5 | 21.4 | -4.0 | -27.8 (63.4) | -50.3 (58.4) | -3.6 | -29.5 | -32.3 (89.0) | -26.8 (92.9) | 38.6 | 18.5 | 7.3 (83.4) | 21.6 (82.9) |
| | | | 0.7 | 9.3 | 28.1 | -0.3 | -29.4 (67.8) | -47.5 (60.5) | -2.0 | -31.0 | -33.1 (89.8) | -30.4 (93.7) | 39.7 | 15.4 | 4.2 (87.1) | 17.6 (86.4) |
| | | 2:1 | 0.1 | 10.7 | 5.3 | -16.1 | -30.7 (66.8) | -71.0 (16.6) | -1.6 | -22.3 | -24.8 (88.1) | -21.2 (90.4) | 52.9 | 40.1 | 25.8 (71.1) | 39.4 (52.5) |
| | | | 0.3 | 10.5 | 8.4 | -16.5 | -35.7 (68.4) | -73.5 (15.2) | -0.3 | -24.4 | -28.2 (84.3) | -23.9 (87.7) | 53.4 | 36.6 | 22.8 (73.0) | 38.1 (58.1) |
| | | | 0.7 | 10.4 | 10.8 | -20.0 | -38.7 (65.8) | -81.9 (14.1) | -0.6 | -32.3 | -33.0 (82.9) | -31.9 (84.2) | 53.8 | 31.2 | 15.4 (81.5) | 32.8 (69.3) |
| | U[50, 200] | 1:1 | 0.1 | 9.0 | 25.3 | 4.7 | -25.0 (64.1) | -32.8 (84.7) | -6.3 | -25.9 | -33.6 (90.8) | -25.1 (96.2) | 8.9 | -10.4 | -20.9 (90.9) | -6.1 (96.9) |
| | | | 0.3 | 9.0 | 33.0 | 10.3 | -19.0 (65.7) | -28.7 (89.4) | -2.6 | -25.6 | -26.9 (90.7) | -24.8 (95.0) | 12.3 | -9.4 | -16.9 (90.3) | -7.1 (95.7) |
| | | | 0.7 | 8.7 | 43.2 | 18.4 | -15.3 (64.8) | -22.5 (90.3) | 0.9 | -27.0 | -24.9 (89.1) | -26.1 (94.6) | 15.4 | -9.0 | -14.7 (90.6) | -11.0 (94.3) |
| | | 2:1 | 0.1 | 11.0 | 13.6 | -3.8 | -27.0 (68.0) | -47.2 (36.4) | -0.4 | -19.0 | -26.5 (87.8) | -16.9 (94.2) | 18.7 | 3.2 | -7.8 (87.7) | 4.5 (95.1) |
| | | | 0.3 | 10.9 | 16.5 | -3.8 | -31.2 (64.1) | -50.3 (30.9) | -1.4 | -21.1 | -25.2 (88.4) | -20.8 (89.9) | 17.5 | 1.1 | -11.5 (91.7) | 1.0 (93.9) |
| | | | 0.7 | 10.6 | 22.2 | -3.7 | -32.2 (69.0) | -51.8 (33.6) | 0.3 | -26.5 | -28.2 (86.9) | -26.5 (89.2) | 19.8 | -5.0 | -15.5 (90.2) | -3.1 (93.1) |
| | U[500, 700] | 1:1 | 0.1 | 8.9 | 29.9 | 11.4 | -21.7 (57.6) | -28.7 (90.6) | -8.1 | -28.8 | -36.4 (89.8) | -25.8 (96.1) | -5.4 | -27.5 | -34.6 (91.0) | -22.7 (96.0) |
| | | | 0.3 | 8.7 | 37.5 | 17.8 | -17.2 (59.4) | -24.5 (92.7) | -2.4 | -23.2 | -28.8 (87.9) | -22.6 (95.7) | -0.2 | -21.0 | -26.6 (88.9) | -18.6 (95.8) |
| | | | 0.7 | 8.6 | 47.1 | 22.7 | -16.9 (58.9) | -20.2 (94.6) | -1.1 | -28.6 | -27.7 (88.7) | -27.4 (95.6) | 2.0 | -25.8 | -25.8 (90.3) | -23.4 (95.0) |
| | | 2:1 | 0.1 | 11.1 | 16.0 | 1.6 | -30.4 (67.2) | -34.1 (70.4) | -4.8 | -19.2 | -27.6 (90.0) | -17.2 (93.9) | -1.5 | -14.2 | -22.9 (91.2) | -13.9 (96.4) |
| | | | 0.3 | 10.9 | 22.5 | 3.7 | -31.0 (65.0) | -33.2 (72.2) | -1.9 | -21.3 | -27.7 (87.8) | -19.2 (93.4) | 1.5 | -17.5 | -24.0 (89.9) | -15.3 (93.6) |
| | | | 0.7 | 10.6 | 28.4 | 3.7 | -30.4 (65.5) | -34.7 (69.8) | -0.5 | -26.2 | -29.1 (88.9) | -25.7 (92.2) | 2.7 | -22.8 | -26.1 (90.0) | -21.8 (93.7) |
| 50 | U[30, 60] | 1:1 | 0.1 | 31.6 | 19.0 | -3.4 | -29.3 (75.7) | -44.2 (14.9) | -1.6 | -23.7 | -30.1 (81.4) | -22.2 (80.4) | 41.0 | 24.6 | 10.4 (84.5) | 25.7 (62.2) |
| | | | 0.3 | 31.3 | 23.7 | -0.8 | -31.5 (75.6) | -43.3 (17.5) | -0.8 | -25.1 | -29.1 (82.7) | -24.7 (81.9) | 41.9 | 22.9 | 8.1 (87.2) | 24.2 (67.4) |
| | | | 0.7 | 31.1 | 29.9 | 1.4 | -32.3 (75.8) | -42.3 (18.6) | -0.6 | -30.3 | -32.4 (77.9) | -30.4 (81.3) | 42.1 | 18.8 | 2.3 (89.4) | 19.2 (79.4) |
| | | 2:1 | 0.1 | 35.8 | 6.9 | -13.9 | -26.6 (78.2) | -74.2 (0.0) | 0.1 | -20.3 | -24.2 (76.5) | -19.7 (73.7) | 54.7 | 41.5 | 27.3 (60.5) | 41.2 (6.0) |
| | | | 0.3 | 35.5 | 8.8 | -15.1 | -32.6 (76.2) | -79.0 (0.1) | -0.0 | -23.4 | -25.3 (78.1) | -23.3 (71.4) | 55.1 | 38.8 | 23.4 (75.2) | 39.1 (13.5) |
| | | | 0.7 | 34.9 | 11.5 | -17.8 | -37.0 (77.0) | -84.9 (0.1) | 0.0 | -30.1 | -28.4 (79.2) | -30.2 (66.6) | 55.0 | 34.0 | 15.0 (87.0) | 34.0 (32.9) |
| | U[50, 200] | 1:1 | 0.1 | 30.1 | 28.1 | 9.5 | -21.6 (74.5) | -15.9 (93.4) | -2.4 | -20.6 | -27.5 (84.3) | -19.8 (87.7) | 12.9 | -3.3 | -14.9 (90.9) | -1.4 (95.1) |
| | | | 0.3 | 30.0 | 34.4 | 13.5 | -21.6 (77.2) | -12.5 (95.3) | -0.3 | -21.0 | -24.0 (85.4) | -20.4 (90.2) | 15.3 | -3.7 | -13.9 (87.9) | -2.6 (95.3) |
| | | | 0.7 | 29.3 | 41.9 | 17.8 | -22.9 (74.8) | -7.3 (97.0) | -1.0 | -26.6 | -27.2 (82.8) | -26.0 (89.5) | 14.6 | -8.7 | -18.7 (85.3) | -7.2 (95.1) |
| | | 2:1 | 0.1 | 37.2 | 14.0 | -1.8 | -25.7 (73.7) | -47.8 (0.9) | -0.8 | -15.7 | -20.9 (82.7) | -15.5 (82.6) | 19.5 | 6.7 | -5.1 (91.9) | 6.8 (91.9) |
| | | | 0.3 | 36.5 | 18.5 | -0.4 | -26.4 (78.3) | -48.6 (0.2) | 0.0 | -18.9 | -19.0 (83.6) | -18.4 (79.7) | 20.2 | 3.9 | -7.4 (89.8) | 4.2 (92.1) |
| | | | 0.7 | 35.6 | 22.8 | -0.7 | -32.1 (77.3) | -50.6 (1.0) | 0.1 | -25.1 | -24.4 (79.5) | -25.0 (76.1) | 20.0 | -2.3 | -15.6 (82.8) | -2.1 (95.2) |
| | U[500, 700] | 1:1 | 0.1 | 29.5 | 30.9 | 13.6 | -27.7 (67.2) | -14.2 (93.8) | -3.6 | -22.1 | -31.3 (83.4) | -20.1 (91.8) | -0.8 | -18.9 | -28.3 (86.8) | -16.2 (94.1) |
| | | | 0.3 | 29.1 | 39.2 | 20.2 | -23.9 (71.9) | -9.2 (96.1) | -1.1 | -21.6 | -24.5 (86.6) | -20.5 (92.1) | 1.6 | -18.7 | -22.3 (87.6) | -17.4 (93.6) |
| | | | 0.7 | 28.6 | 47.4 | 25.5 | -26.7 (66.2) | -5.0 (96.9) | -1.4 | -26.0 | -25.5 (79.8) | -25.4 (92.7) | 1.3 | -22.9 | -23.2 (80.6) | -21.3 (92.6) |
| | | 2:1 | 0.1 | 37.0 | 19.1 | 4.4 | -25.1 (75.0) | -29.4 (17.7) | -0.8 | -15.2 | -19.8 (85.8) | -14.4 (87.6) | 2.7 | -11.7 | -15.9 (88.4) | -10.9 (92.0) |
| | | | 0.3 | 36.5 | 24.7 | 7.6 | -27.1 (76.2) | -28.2 (24.0) | -0.1 | -16.8 | -19.5 (83.5) | -16.4 (84.9) | 3.4 | -13.1 | -16.1 (85.5) | -12.6 (90.1) |
| | | | 0.7 | 35.6 | 29.4 | 7.6 | -33.6 (72.7) | -27.8 (26.7) | -0.5 | -23.8 | -25.3 (79.8) | -23.8 (81.6) | 2.9 | -20.0 | -21.8 (81.2) | -19.8 (86.4) |

Results of $NN_P$, $HN_P$, and $CBN_P$ are based on the population studies; results of $NN_O$, $HN_O$, and $CBN_O$ are based on the published studies; CN and CS indicate the Copas-N and Copas-Shi methods; $HN^{Prop}$ and $CBN^{Prop}$ indicate the proposed HN or CBN model based sensitivity analysis methods; $HN^{Hu}$ and $CBN^{Hu}$ indicate the $t$-statistic based sensitivity analysis methods; CP indicates the coverage probability.

**TABLE B6** Convergence proportion (%) of estimations among different models under the 2SBN model based data-generating process.

| $S$ | Patients | T:C | $\tau^2$ | $N$ | $NN_P$ | $NN_O$ | CN | CS | $HN_P$ | $HN_O$ | $HN^{Prop}$ | $HN^{Hu}$ | $CBN_P$ | $CBN_O$ | $CBN^{Prop}$ | $CBN^{Hu}$ |
|---|---|---|---|---|---|---|---|---|---|---|---|---|---|---|---|---|
| 15 | U[30, 60] | 1:1 | 0.1 | 10.6 | 100.0 | 100.0 | 100 | 100.0 | 66.9 | 67.1 | 58.4 | 99.6 | 61.4 | 63.3 | 62.8 | 84.1 |
| | | | 0.3 | 10.6 | 100.0 | 100.0 | 100 | 100.0 | 70.7 | 68.4 | 64.7 | 99.8 | 68.2 | 66.2 | 69.6 | 85.8 |
| | | | 0.7 | 10.6 | 99.7 | 99.7 | 100 | 100.0 | 77.9 | 77.1 | 76.6 | 99.9 | 77.0 | 72.8 | 77.9 | 83.9 |
| | | 2:1 | 0.1 | 10.6 | 100.0 | 99.7 | 100 | 100.0 | 67.7 | 64.6 | 57.1 | 99.6 | 64.3 | 64.7 | 62.1 | 83.3 |
| | | | 0.3 | 10.6 | 100.0 | 99.8 | 100 | 100.0 | 70.4 | 71.2 | 66.7 | 99.8 | 66.6 | 66.3 | 71.1 | 81.6 |
| | | | 0.7 | 10.5 | 100.0 | 99.9 | 100 | 100.0 | 79.0 | 76.0 | 77.9 | 99.9 | 76.6 | 75.6 | 80.7 | 83.5 |
| | U[50, 200] | 1:1 | 0.1 | 10.8 | 100.0 | 100.0 | 100 | 100.0 | 68.6 | 70.4 | 62.8 | 99.8 | 65.9 | 67.6 | 65.7 | 84.4 |
| | | | 0.3 | 10.8 | 99.7 | 99.9 | 100 | 100.0 | 80.2 | 77.1 | 74.3 | 99.9 | 76.2 | 74.3 | 77.1 | 83.5 |
| | | | 0.7 | 10.9 | 99.1 | 99.8 | 100 | 100.0 | 90.5 | 89.3 | 88.4 | 99.9 | 89.9 | 85.6 | 89.1 | 86.2 |
| | | 2:1 | 0.1 | 10.9 | 100.0 | 99.9 | 100 | 100.0 | 70.5 | 69.5 | 65.2 | 99.8 | 66.6 | 66.7 | 66.3 | 82.0 |
| | | | 0.3 | 10.8 | 99.8 | 99.7 | 100 | 100.0 | 82.3 | 80.7 | 75.4 | 99.9 | 79.6 | 77.4 | 78.2 | 84.2 |
| | | | 0.7 | 10.8 | 99.2 | 99.7 | 100 | 100.0 | 91.9 | 89.2 | 87.8 | 100.0 | 91.2 | 88.8 | 89.6 | 83.6 |
| | U[500, 700] | 1:1 | 0.1 | 10.4 | 100.0 | 100.0 | 100 | 100.0 | 87.3 | 82.1 | 76.4 | 100.0 | 86.5 | 80.7 | 79.0 | 74.8 |
| | | | 0.3 | 10.4 | 99.9 | 99.9 | 100 | 100.0 | 97.3 | 94.1 | 89.5 | 100.0 | 97.0 | 93.9 | 90.5 | 73.7 |
| | | | 0.7 | 10.4 | 100.0 | 99.9 | 100 | 100.0 | 99.6 | 97.6 | 96.2 | 100.0 | 99.6 | 97.2 | 97.0 | 75.4 |
| | | 2:1 | 0.1 | 10.4 | 99.9 | 100.0 | 100 | 100.0 | 89.8 | 83.0 | 77.2 | 100.0 | 88.4 | 80.1 | 79.3 | 73.3 |
| | | | 0.3 | 10.5 | 99.9 | 100.0 | 100 | 100.0 | 98.3 | 94.0 | 91.5 | 100.0 | 98.4 | 95.5 | 92.6 | 74.3 |
| | | | 0.7 | 10.4 | 100.0 | 100.0 | 100 | 100.0 | 99.9 | 98.7 | 97.5 | 100.0 | 99.9 | 99.5 | 98.4 | 70.6 |
| 50 | U[30, 60] | 1:1 | 0.1 | 35.9 | 100.0 | 100.0 | 100 | 100.0 | 72.3 | 70.2 | 67.0 | 99.6 | 67.6 | 65.0 | 67.8 | 95.4 |
| | | | 0.3 | 36.0 | 100.0 | 100.0 | 100 | 100.0 | 79.4 | 74.7 | 77.7 | 99.6 | 75.1 | 71.7 | 76.8 | 96.3 |
| | | | 0.7 | 36.1 | 98.7 | 98.5 | 100 | 100.0 | 89.3 | 89.3 | 90.9 | 100.0 | 89.1 | 86.1 | 90.1 | 96.1 |
| | | 2:1 | 0.1 | 35.8 | 100.0 | 100.0 | 100 | 100.0 | 71.4 | 69.9 | 67.3 | 99.8 | 65.7 | 67.8 | 66.9 | 95.7 |
| | | | 0.3 | 36.0 | 100.0 | 99.9 | 100 | 100.0 | 79.7 | 77.4 | 79.8 | 99.9 | 78.0 | 75.2 | 76.3 | 96.8 |
| | | | 0.7 | 35.9 | 99.6 | 99.7 | 100 | 99.9 | 93.2 | 90.5 | 91.6 | 100.0 | 90.4 | 88.6 | 90.7 | 96.1 |
| | U[50, 200] | 1:1 | 0.1 | 36.9 | 99.6 | 99.5 | 100 | 100.0 | 79.8 | 75.9 | 72.0 | 99.9 | 75.5 | 70.7 | 70.2 | 96.1 |
| | | | 0.3 | 36.9 | 99.6 | 99.5 | 100 | 100.0 | 92.9 | 89.2 | 91.8 | 100.0 | 92.3 | 89.2 | 90.4 | 96.8 |
| | | | 0.7 | 36.9 | 99.7 | 99.6 | 100 | 100.0 | 99.0 | 98.2 | 98.0 | 100.0 | 98.6 | 97.9 | 98.0 | 96.5 |
| | | 2:1 | 0.1 | 37.0 | 99.3 | 99.6 | 100 | 100.0 | 79.4 | 79.0 | 73.9 | 100.0 | 78.5 | 74.5 | 71.0 | 96.1 |
| | | | 0.3 | 37.0 | 99.9 | 99.7 | 100 | 100.0 | 94.4 | 92.3 | 91.3 | 100.0 | 92.5 | 89.6 | 92.0 | 96.9 |
| | | | 0.7 | 37.0 | 99.5 | 99.4 | 100 | 100.0 | 99.6 | 98.5 | 98.3 | 100.0 | 99.5 | 98.0 | 99.0 | 97.0 |
| | U[500, 700] | 1:1 | 0.1 | 35.3 | 100.0 | 99.8 | 100 | 100.0 | 96.8 | 93.4 | 92.0 | 100.0 | 96.2 | 91.5 | 91.3 | 95.4 |
| | | | 0.3 | 35.3 | 100.0 | 100.0 | 100 | 100.0 | 100.0 | 99.7 | 98.9 | 100.0 | 100.0 | 99.5 | 99.5 | 94.4 |
| | | | 0.7 | 35.3 | 100.0 | 100.0 | 100 | 100.0 | 100.0 | 100.0 | 99.8 | 100.0 | 100.0 | 100.0 | 100.0 | 95.5 |
| | | 2:1 | 0.1 | 35.4 | 99.9 | 99.9 | 100 | 100.0 | 98.0 | 94.4 | 93.1 | 100.0 | 98.0 | 93.1 | 91.3 | 95.0 |
| | | | 0.3 | 35.4 | 100.0 | 100.0 | 100 | 100.0 | 100.0 | 99.9 | 99.5 | 100.0 | 100.0 | 100.0 | 99.9 | 96.5 |
| | | | 0.7 | 35.3 | 100.0 | 100.0 | 100 | 100.0 | 100.0 | 100.0 | 99.7 | 100.0 | 100.0 | 100.0 | 100.0 | 94.9 |

Results of $NN_P$, $HN_P$, and $CBN_P$ are based on the population studies; results of $NN_O$, $HN_O$, and $CBN_O$ are based on the published studies; CN and CS indicate the Copas-N and Copas-Shi methods; $HN^{Prop}$ and $CBN^{Prop}$ indicate the proposed HN or CBN model based sensitivity analysis methods; $HN^{Hu}$ and $CBN^{Hu}$ indicate the $t$-statistic based sensitivity analysis methods.

**TABLE B7** Averages of estimates of $\tau^2$ among different models under the 2SBN model based data-generating process.

| $S$ | Patients | T:C | $\tau^2$ | $N$ | $NN_P$ | $NN_O$ | CN | CS | $HN_P$ | $HN_O$ | $HN^{Prop}$ | $HN^{Hu}$ | $CBN_P$ | $CBN_O$ | $CBN^{Prop}$ | $CBN^{Hu}$ |
|---|---|---|---|---|---|---|---|---|---|---|---|---|---|---|---|---|
| 15 | U[30, 60] | 1:1 | 0.1 | 10.6 | 0.00 | 0.01 | 1.13 | 0.00 | 0.64 | 0.68 | 0.98 | 0.46 | 0.55 | 0.58 | 0.83 | 0.36 |
| | | | 0.3 | 10.6 | 0.01 | 0.01 | 1.12 | 0.00 | 0.67 | 0.68 | 1.03 | 0.48 | 0.54 | 0.56 | 0.84 | 0.37 |
| | | | 0.7 | 10.6 | 0.03 | 0.04 | 1.25 | 0.01 | 1.02 | 0.93 | 1.31 | 0.72 | 0.83 | 0.80 | 1.10 | 0.58 |
| | | 2:1 | 0.1 | 10.6 | 0.00 | 0.01 | 1.03 | 0.00 | 0.60 | 0.64 | 0.90 | 0.41 | 0.48 | 0.51 | 0.74 | 0.33 |
| | | | 0.3 | 10.6 | 0.01 | 0.01 | 1.09 | 0.00 | 0.73 | 0.72 | 1.07 | 0.51 | 0.61 | 0.62 | 0.86 | 0.44 |
| | | | 0.7 | 10.5 | 0.03 | 0.04 | 1.19 | 0.01 | 0.99 | 0.94 | 1.26 | 0.72 | 0.84 | 0.78 | 1.06 | 0.59 |
| | U[50, 200] | 1:1 | 0.1 | 10.8 | 0.02 | 0.03 | 1.18 | 0.01 | 0.25 | 0.24 | 0.37 | 0.17 | 0.21 | 0.20 | 0.31 | 0.14 |
| | | | 0.3 | 10.8 | 0.05 | 0.06 | 1.26 | 0.02 | 0.40 | 0.38 | 0.53 | 0.29 | 0.35 | 0.33 | 0.45 | 0.24 |
| | | | 0.7 | 10.9 | 0.17 | 0.18 | 1.39 | 0.07 | 0.71 | 0.64 | 0.80 | 0.58 | 0.60 | 0.56 | 0.69 | 0.47 |
| | | 2:1 | 0.1 | 10.9 | 0.02 | 0.03 | 0.92 | 0.01 | 0.21 | 0.20 | 0.31 | 0.14 | 0.17 | 0.17 | 0.26 | 0.11 |
| | | | 0.3 | 10.8 | 0.07 | 0.08 | 1.06 | 0.03 | 0.41 | 0.39 | 0.54 | 0.32 | 0.35 | 0.34 | 0.46 | 0.27 |
| | | | 0.7 | 10.8 | 0.20 | 0.21 | 1.20 | 0.10 | 0.74 | 0.67 | 0.84 | 0.60 | 0.64 | 0.58 | 0.72 | 0.52 |
| | U[500, 700] | 1:1 | 0.1 | 10.4 | 0.06 | 0.05 | 0.77 | 0.02 | 0.10 | 0.10 | 0.14 | 0.08 | 0.09 | 0.09 | 0.13 | 0.07 |
| | | | 0.3 | 10.4 | 0.18 | 0.16 | 0.89 | 0.08 | 0.28 | 0.24 | 0.30 | 0.23 | 0.26 | 0.22 | 0.28 | 0.21 |
| | | | 0.7 | 10.4 | 0.44 | 0.39 | 1.14 | 0.25 | 0.65 | 0.55 | 0.65 | 0.54 | 0.59 | 0.49 | 0.59 | 0.49 |
| | | 2:1 | 0.1 | 10.4 | 0.06 | 0.06 | 0.50 | 0.03 | 0.10 | 0.09 | 0.13 | 0.08 | 0.09 | 0.08 | 0.11 | 0.07 |
| | | | 0.3 | 10.5 | 0.20 | 0.17 | 0.67 | 0.11 | 0.27 | 0.24 | 0.30 | 0.23 | 0.25 | 0.22 | 0.27 | 0.21 |
| | | | 0.7 | 10.4 | 0.48 | 0.42 | 0.96 | 0.30 | 0.65 | 0.55 | 0.65 | 0.54 | 0.59 | 0.49 | 0.58 | 0.49 |
| 50 | U[30, 60] | 1:1 | 0.1 | 35.9 | 0.00 | 0.00 | 1.16 | 0.00 | 0.35 | 0.37 | 0.51 | 0.26 | 0.26 | 0.27 | 0.39 | 0.18 |
| | | | 0.3 | 36.0 | 0.00 | 0.00 | 1.18 | 0.00 | 0.50 | 0.54 | 0.68 | 0.40 | 0.39 | 0.41 | 0.54 | 0.29 |
| | | | 0.7 | 36.1 | 0.01 | 0.02 | 1.25 | 0.00 | 0.81 | 0.74 | 0.93 | 0.66 | 0.61 | 0.57 | 0.72 | 0.49 |
| | | 2:1 | 0.1 | 35.8 | 0.00 | 0.00 | 1.02 | 0.00 | 0.32 | 0.34 | 0.48 | 0.24 | 0.23 | 0.23 | 0.36 | 0.16 |
| | | | 0.3 | 36.0 | 0.00 | 0.00 | 1.04 | 0.00 | 0.46 | 0.48 | 0.63 | 0.37 | 0.33 | 0.35 | 0.50 | 0.27 |
| | | | 0.7 | 35.9 | 0.01 | 0.02 | 1.14 | 0.01 | 0.85 | 0.80 | 0.98 | 0.73 | 0.67 | 0.62 | 0.78 | 0.55 |
| | U[50, 200] | 1:1 | 0.1 | 36.9 | 0.01 | 0.01 | 1.29 | 0.00 | 0.16 | 0.16 | 0.22 | 0.12 | 0.13 | 0.13 | 0.19 | 0.09 |
| | | | 0.3 | 36.9 | 0.04 | 0.06 | 1.35 | 0.01 | 0.33 | 0.31 | 0.37 | 0.28 | 0.27 | 0.26 | 0.32 | 0.23 |
| | | | 0.7 | 36.9 | 0.18 | 0.20 | 1.45 | 0.08 | 0.70 | 0.63 | 0.72 | 0.62 | 0.59 | 0.52 | 0.61 | 0.51 |
| | | 2:1 | 0.1 | 37.0 | 0.01 | 0.01 | 0.96 | 0.00 | 0.15 | 0.15 | 0.21 | 0.12 | 0.11 | 0.12 | 0.17 | 0.08 |
| | | | 0.3 | 37.0 | 0.04 | 0.05 | 1.02 | 0.02 | 0.31 | 0.29 | 0.35 | 0.26 | 0.26 | 0.24 | 0.29 | 0.21 |
| | | | 0.7 | 37.0 | 0.20 | 0.22 | 1.21 | 0.11 | 0.71 | 0.64 | 0.74 | 0.63 | 0.60 | 0.54 | 0.63 | 0.53 |
| | U[500, 700] | 1:1 | 0.1 | 35.3 | 0.05 | 0.05 | 0.87 | 0.02 | 0.10 | 0.09 | 0.11 | 0.08 | 0.09 | 0.08 | 0.10 | 0.07 |
| | | | 0.3 | 35.3 | 0.20 | 0.18 | 1.02 | 0.10 | 0.29 | 0.25 | 0.30 | 0.25 | 0.27 | 0.23 | 0.27 | 0.23 |
| | | | 0.7 | 35.3 | 0.47 | 0.43 | 1.28 | 0.32 | 0.68 | 0.59 | 0.68 | 0.59 | 0.61 | 0.52 | 0.60 | 0.52 |
| | | 2:1 | 0.1 | 35.4 | 0.06 | 0.05 | 0.57 | 0.03 | 0.09 | 0.08 | 0.10 | 0.08 | 0.08 | 0.07 | 0.09 | 0.07 |
| | | | 0.3 | 35.4 | 0.22 | 0.20 | 0.72 | 0.14 | 0.30 | 0.26 | 0.30 | 0.26 | 0.27 | 0.23 | 0.27 | 0.23 |
| | | | 0.7 | 35.3 | 0.50 | 0.46 | 1.03 | 0.36 | 0.69 | 0.60 | 0.69 | 0.60 | 0.62 | 0.53 | 0.62 | 0.53 |

Results of $NN_P$, $HN_P$, and $CBN_P$ are based on the population studies; results of $NN_O$, $HN_O$, and $CBN_O$ are based on the published studies; CN and CS indicate the Copas-N and Copas-Shi methods; $HN^{Prop}$ and $CBN^{Prop}$ indicate the proposed HN or CBN model based sensitivity analysis methods; $HN^{Hu}$ and $CBN^{Hu}$ indicate the $t$-statistic based sensitivity analysis methods.

**TABLE B8** Averages of estimation bias of the lnOR ($\theta = -2$) among different models under the 2SBN model based data-generating process and the Hedges-type selection model (B4). All estimates were multiplied by 100.

| $S$ | Patients | T:C | $\tau^2$ | $N$ | $NN_P$ | $NN_O$ | CN (CP) | CS (CP) | $HN_P$ | $HN_O$ | $HN^{Prop}$ (CP) | $HN^{HT}$ (CP) | $CBN_P$ | $CBN_O$ | $CBN^{Prop}$ (CP) | $CBN^{HT}$ (CP) |
|---|---|---|---|---|---|---|---|---|---|---|---|---|---|---|---|---|
| 15 | U[30, 60] | 1:1 | 0.1 | 8.2 | 66.2 | 40.4 | 22.1 (71.8) | 41.7 (74.0) | -17.9 | -49.9 | -56.1 (93.3) | -52.7 (98.9) | -1.2 | -33.5 | -41.3 (89.8) | -42.5 (96.9) |
| | | | 0.3 | 8.1 | 71.3 | 44.9 | 26.8 (72.7) | 40.8 (75.4) | -11.8 | -47.0 | -54.7 (90.3) | -47.5 (98.3) | 6.0 | -31.0 | -32.6 (90.2) | -34.0 (95.5) |
| | | | 0.7 | 8.1 | 77.5 | 49.2 | 31.8 (71.7) | 39.2 (81.2) | -3.0 | -41.6 | -41.5 (87.2) | -41.4 (97.1) | 13.8 | -18.0 | -21.2 (88.0) | -25.9 (95.5) |
| | | 2:1 | 0.1 | 8.2 | 43.0 | 16.4 | 9.4 (67.2) | 14.2 (82.2) | -11.4 | -47.3 | -48.8 (91.4) | -44.6 (98.3) | 2.9 | -31.2 | -34.7 (88.2) | -28.9 (98.8) |
| | | | 0.3 | 8.3 | 46.0 | 16.6 | 8.0 (69.6) | 10.0 (83.0) | -4.7 | -45.2 | -53.1 (91.7) | -42.2 (97.2) | 10.6 | -25.8 | -33.7 (91.0) | -24.8 (96.4) |
| | | | 0.7 | 8.2 | 56.7 | 26.2 | 14.4 (68.7) | 13.8 (86.7) | 6.0 | -40.6 | -42.5 (90.0) | -37.6 (96.2) | 21.9 | -22.4 | -24.2 (87.5) | -21.5 (94.8) |
| | U[50, 200] | 1:1 | 0.1 | 10.4 | 33.5 | 18.3 | -5.3 (67.9) | -19.1 (77.2) | -5.2 | -18.5 | -18.7 (88.6) | -14.1 (96.5) | 9.2 | -0.8 | -3.8 (86.5) | 3.4 (92.7) |
| | | | 0.3 | 10.3 | 40.5 | 23.2 | 0.1 (67.2) | -13.1 (77.4) | -3.1 | -17.0 | -19.3 (90.2) | -14.3 (95.9) | 12.2 | -1.6 | -3.0 (86.1) | 1.9 (90.8) |
| | | | 0.7 | 10.1 | 52.3 | 30.6 | 2.8 (69.8) | -8.2 (77.8) | 4.3 | -12.1 | -12.5 (91.1) | -11.5 (94.1) | 21.2 | 4.9 | 4.7 (85.1) | 6.2 (88.0) |
| | | 2:1 | 0.1 | 10.5 | 22.6 | 9.9 | -5.1 (69.8) | -27.7 (62.2) | -2.6 | -13.9 | -14.8 (88.5) | -10.4 (96.7) | 13.8 | 3.9 | 1.7 (83.9) | 6.0 (93.1) |
| | | | 0.3 | 10.4 | 29.3 | 12.9 | -5.6 (67.1) | -26.7 (66.3) | 1.8 | -14.6 | -16.1 (90.9) | -12.0 (94.1) | 17.7 | 3.9 | 1.7 (86.7) | 5.4 (90.6) |
| | | | 0.7 | 10.1 | 40.0 | 20.0 | -2.0 (70.9) | -20.9 (71.1) | 6.7 | -12.2 | -11.3 (89.2) | -11.5 (91.8) | 23.4 | 5.0 | 5.2 (85.2) | 5.4 (87.2) |
| | U[500, 700] | 1:1 | 0.1 | 12.0 | 9.6 | 7.8 | -1.8 (67.0) | -24.4 (50.7) | -1.0 | -1.3 | -3.6 (83.4) | -1.0 (93.1) | 14.5 | 13.9 | 10.7 (77.3) | 14.6 (77.8) |
| | | | 0.3 | 11.9 | 16.7 | 13.9 | 4.5 (67.0) | -17.4 (69.6) | 2.2 | 1.9 | 1.0 (81.9) | 1.7 (90.6) | 17.1 | 16.8 | 15.7 (74.8) | 17.0 (79.0) |
| | | | 0.7 | 11.7 | 22.7 | 15.9 | 4.3 (69.9) | -12.0 (76.6) | 2.8 | -1.0 | -1.3 (79.6) | -1.0 (89.6) | 17.7 | 14.4 | 13.2 (78.2) | 13.6 (82.9) |
| | | 2:1 | 0.1 | 12.0 | 7.3 | 6.2 | -0.4 (67.2) | -22.2 (50.8) | 1.0 | 0.8 | -2.9 (85.9) | 0.9 (92.9) | 15.9 | 16.0 | 13.0 (77.7) | 16.3 (74.3) |
| | | | 0.3 | 11.9 | 11.5 | 9.5 | 2.6 (72.8) | -19.1 (66.3) | 2.3 | 1.6 | 0.9 (80.1) | 1.5 (91.6) | 17.1 | 16.6 | 15.7 (76.4) | 17.4 (79.4) |
| | | | 0.7 | 11.8 | 17.0 | 10.6 | 3.8 (69.0) | -15.9 (76.1) | 3.6 | -1.0 | -1.2 (74.9) | -1.0 (90.6) | 18.2 | 14.1 | 13.1 (76.2) | 13.8 (86.3) |
| 50 | U[30, 60] | 1:1 | 0.1 | 26.3 | 73.4 | 46.7 | 26.8 (77.1) | 31.7 (79.4) | -7.0 | -42.0 | -51.7 (91.9) | -37.2 (94.4) | 10.8 | -23.2 | -32.9 (92.8) | -16.6 (97.1) |
| | | | 0.3 | 26.3 | 76.8 | 50.5 | 32.5 (79.3) | 31.8 (79.6) | -4.0 | -37.5 | -47.5 (93.1) | -33.9 (95.5) | 13.2 | -18.1 | -26.7 (90.5) | -13.1 (96.5) |
| | | | 0.7 | 26.3 | 85.1 | 56.8 | 37.2 (77.0) | 31.8 (80.1) | 2.0 | -33.1 | -37.4 (92.8) | -31.0 (96.4) | 21.6 | -9.6 | -14.9 (90.8) | -8.5 (95.4) |
| | | 2:1 | 0.1 | 26.9 | 47.5 | 19.8 | 11.9 (79.1) | 6.3 (82.9) | -4.2 | -39.8 | -46.1 (89.4) | -35.8 (92.5) | 12.0 | -19.6 | -27.3 (90.0) | -15.5 (96.5) |
| | | | 0.3 | 27.0 | 51.6 | 24.4 | 16.5 (80.4) | 2.8 (84.5) | 0.1 | -33.3 | -34.4 (92.1) | -31.1 (95.6) | 17.3 | -13.8 | -15.6 (89.5) | -10.5 (96.9) |
| | | | 0.7 | 26.7 | 61.3 | 32.1 | 23.9 (75.8) | 3.0 (86.6) | 9.7 | -27.1 | -24.5 (89.7) | -26.0 (95.5) | 27.9 | -6.1 | -4.9 (87.4) | -3.7 (93.6) |
| | U[50, 200] | 1:1 | 0.1 | 33.3 | 39.7 | 23.1 | -0.5 (73.1) | -18.5 (67.1) | -1.2 | -11.1 | -11.3 (88.7) | -9.7 (95.1) | 15.0 | 5.8 | 4.8 (83.2) | 7.2 (91.4) |
| | | | 0.3 | 32.9 | 46.6 | 28.6 | -0.6 (69.9) | -14.6 (74.0) | 1.7 | -10.0 | -7.2 (89.7) | -9.2 (93.9) | 18.0 | 7.7 | 9.3 (81.6) | 8.2 (89.9) |
| | | | 0.7 | 32.2 | 57.7 | 35.9 | 3.6 (71.7) | -7.1 (82.5) | 4.7 | -11.4 | -8.6 (87.0) | -11.1 (94.5) | 21.8 | 7.0 | 9.2 (82.3) | 7.5 (88.7) |
| | | 2:1 | 0.1 | 33.4 | 26.0 | 12.5 | 1.3 (84.2) | -32.0 (37.1) | 1.0 | -10.2 | -9.3 (88.3) | -8.7 (95.3) | 16.9 | 7.1 | 6.8 (79.5) | 8.4 (88.8) |
| | | | 0.3 | 33.0 | 32.8 | 16.9 | -0.4 (79.9) | -28.3 (48.3) | 3.2 | -9.9 | -7.7 (86.3) | -8.8 (92.9) | 19.7 | 8.2 | 9.7 (78.1) | 9.0 (86.8) |
| | | | 0.7 | 32.4 | 44.0 | 24.1 | 0.4 (78.2) | -21.5 (65.3) | 8.4 | -9.2 | -7.4 (84.3) | -9.0 (94.4) | 24.8 | 9.1 | 10.1 (79.1) | 8.7 (89.6) |
| | U[500, 700] | 1:1 | 0.1 | 38.0 | 12.6 | 10.3 | 6.7 (78.7) | -22.0 (33.4) | 0.6 | 0.5 | -0.3 (80.4) | 0.6 (93.4) | 16.0 | 16.0 | 15.1 (72.0) | 16.1 (54.0) |
| | | | 0.3 | 37.8 | 17.7 | 13.8 | 11.2 (81.3) | -15.4 (56.9) | 1.1 | 0.5 | -0.5 (75.6) | 0.5 (94.2) | 16.1 | 15.7 | 14.7 (76.8) | 15.7 (69.1) |
| | | | 0.7 | 37.3 | 25.7 | 17.0 | 12.8 (82.9) | -6.0 (75.9) | 2.3 | -1.5 | -2.6 (76.3) | -1.5 (93.2) | 17.0 | 13.7 | 13.1 (76.6) | 13.6 (81.3) |
| | | 2:1 | 0.1 | 38.3 | 8.6 | 7.1 | 5.2 (80.1) | -22.1 (41.5) | 1.3 | 1.1 | 0.1 (79.7) | 1.1 (93.4) | 16.5 | 16.5 | 15.3 (72.1) | 16.5 (50.5) |
| | | | 0.3 | 38.0 | 13.4 | 10.3 | 11.4 (80.3) | -16.7 (53.7) | 2.8 | 1.8 | 2.5 (78.1) | 1.8 (91.5) | 17.6 | 16.8 | 17.6 (74.9) | 16.8 (66.2) |
| | | | 0.7 | 37.5 | 19.3 | 12.3 | 11.9 (80.6) | -7.1 (71.6) | 3.6 | -0.6 | 0.2 (79.2) | -0.6 (93.8) | 18.0 | 14.4 | 15.0 (75.7) | 14.5 (80.0) |

Results of $NN_P$, $HN_P$, and $CBN_P$ are based on the population studies; results of $NN_O$, $HN_O$, and $CBN_O$ are based on the published studies; CN and CS indicate the Copas-N and Copas-Shi methods; $HN^t$*extProp* and $CBN^t$*extProp* indicate the proposed HN or CBN model based sensitivity analysis methods; $HN^{Hu}$ and $CBN^{Hu}$ indicate the $t$-statistic based sensitivity analysis methods CP indicates the coverage probability.

**TABLE B9** Convergence proportion (%) of estimations among different models under the SGBN model based data-generating process.

| $S$ | Patients | $\tau^2$ | $N$ | $NN_P$ | $NN_O$ | CN | CS | $1SBN_P$ | $1SBN_O$ | $1SBN^{prop}$ | $1SBN^{Hu}$ |
|---|---|---|---|---|---|---|---|---|---|---|---|
| 15 | U[15, 30] | 0.1 | 10.6 | 100.0 | 99.9 | 100 | 100 | 78.3 | 75.5 | 75.1 | 72.6 |
| | | 0.3 | 10.6 | 99.7 | 99.8 | 100 | 100 | 92.9 | 88.9 | 89.7 | 73.6 |
| | | 0.7 | 10.6 | 100.0 | 100.0 | 100 | 100 | 98.1 | 96.0 | 96.8 | 72.3 |
| | U[25, 100] | 0.1 | 10.8 | 100.0 | 99.9 | 100 | 100 | 91.3 | 88.4 | 88.0 | 75.3 |
| | | 0.3 | 10.8 | 100.0 | 99.9 | 100 | 100 | 98.4 | 97.9 | 97.8 | 73.9 |
| | | 0.7 | 10.8 | 100.0 | 100.0 | 100 | 100 | 100.0 | 99.5 | 99.7 | 74.0 |
| 50 | U[15, 30] | 0.1 | 36.0 | 99.5 | 99.9 | 100 | 100 | 90.4 | 86.6 | 86.9 | 85.4 |
| | | 0.3 | 36.1 | 100.0 | 99.6 | 100 | 100 | 99.6 | 98.1 | 98.5 | 87.8 |
| | | 0.7 | 36.2 | 100.0 | 100.0 | 100 | 100 | 100.0 | 99.8 | 99.8 | 88.3 |
| | U[25, 100] | 0.1 | 37.0 | 100.0 | 100.0 | 100 | 100 | 99.3 | 97.9 | 98.7 | 89.8 |
| | | 0.3 | 37.1 | 100.0 | 100.0 | 100 | 100 | 100.0 | 100.0 | 99.9 | 90.4 |
| | | 0.7 | 37.0 | 100.0 | 100.0 | 100 | 100 | 100.0 | 100.0 | 100.0 | 90.8 |

$NN_P$ and $BN_P$ are the estimates based on the population studies; $NN_O$ and $BN_O$ are the estimates based on the published studies; CN and CS are the Copas-N and Copas-Shi methods; $1SBN^{prop}$ are the proposed 1SBN model based sensitivity analysis methods; $1SBN^{Hu}$ indicate the $t$-statistic based sensitivity analysis methods.

**TABLE B10** Averages of estimates of $\tau^2$ among different models under the SGBN model based data-generating process.

| $S$ | Patients | $\tau^2$ | $N$ | $NN_P$ | $NN_O$ | CN | CS | $1SBN_P$ | $1SBN_O$ | $1SBN^{prop}$ | $1SBN^{Hu}$ |
|---|---|---|---|---|---|---|---|---|---|---|---|
| 15 | U[15, 30] | 0.1 | 10.6 | 0.04 | 0.04 | 0.52 | 0.00 | 0.14 | 0.14 | 0.18 | 0.10 |
| | | 0.3 | 10.6 | 0.12 | 0.12 | 0.67 | 0.00 | 0.30 | 0.29 | 0.35 | 0.26 |
| | | 0.7 | 10.6 | 0.32 | 0.30 | 0.83 | 0.01 | 0.59 | 0.53 | 0.59 | 0.57 |
| | U[25, 100] | 0.1 | 10.8 | 0.06 | 0.06 | 0.26 | 0.00 | 0.10 | 0.09 | 0.12 | 0.08 |
| | | 0.3 | 10.8 | 0.20 | 0.19 | 0.44 | 0.05 | 0.28 | 0.25 | 0.28 | 0.25 |
| | | 0.7 | 10.8 | 0.47 | 0.45 | 0.73 | 0.22 | 0.61 | 0.54 | 0.59 | 0.57 |
| 50 | U[15, 30] | 0.1 | 36.0 | 0.03 | 0.03 | 0.55 | 0.00 | 0.11 | 0.10 | 0.13 | 0.09 |
| | | 0.3 | 36.1 | 0.12 | 0.11 | 0.72 | 0.00 | 0.29 | 0.26 | 0.30 | 0.25 |
| | | 0.7 | 36.2 | 0.35 | 0.34 | 0.93 | 0.00 | 0.66 | 0.59 | 0.65 | 0.60 |
| | U[25, 100] | 0.1 | 37.0 | 0.06 | 0.06 | 0.26 | 0.00 | 0.10 | 0.08 | 0.10 | 0.08 |
| | | 0.3 | 37.1 | 0.22 | 0.21 | 0.47 | 0.07 | 0.29 | 0.26 | 0.29 | 0.26 |
| | | 0.7 | 37.0 | 0.52 | 0.50 | 0.84 | 0.30 | 0.68 | 0.60 | 0.67 | 0.60 |

$NN_P$ and $BN_P$ are the estimates based on the population studies; $NN_O$ and $BN_O$ are the estimates based on the published studies; CN and CS are the Copas-N and Copas-Shi methods; $1SBN^{prop}$ are the proposed 1SBN model based sensitivity analysis methods; $1SBN^{Hu}$ indicate the $t$-statistic based sensitivity analysis methods.

**TABLE** B11 Averages of estimation bias of the log odds ($\theta = -2$) among different models under the 1SBN model based data-generating process and the Hedges-type selection model (B5). All estimates were multiplied by 100.

| $S$ | Patients | $\tau^2$ | $N$ | $NN_P$ | $NN_O$ | CN (CP) | CS (CP) | $1SBN_P$ | $1SBN_O$ | $1SBN^{prop}$ (CP) | $1SBN^{Hu}$ (CP) |
|---|---|---|---|---|---|---|---|---|---|---|---|
| 15 | U[15, 30] | 0.1 | 11.0 | 18.7 | 10.7 | -14.9 (77.5) | -16.6 (81.4) | -1.5 | -7.2 | -10.6 (88.5) | -6.7 (95.2) |
| | | 0.3 | 10.6 | 24.7 | 12.7 | -20.3 (75.4) | -21.1 (75.3) | -0.2 | -9.1 | -9.7 (88.9) | -8.0 (92.6) |
| | | 0.7 | 10.3 | 29.7 | 14.1 | -22.2 (77.3) | -24.1 (75.6) | 0.8 | -14.4 | -11.8 (91.0) | -12.6 (93.7) |
| | U[25, 100] | 0.1 | 13.0 | 7.5 | 6.4 | -6.6 (86.4) | 1.8 (75.2) | -1.1 | -2.0 | -4.8 (91.5) | -1.7 (94.0) |
| | | 0.3 | 12.9 | 10.5 | 7.8 | -7.8 (82.9) | -3.4 (71.8) | -0.1 | -2.7 | -5.1 (88.7) | -2.3 (92.2) |
| | | 0.7 | 12.6 | 13.2 | 5.2 | -14.2 (82.6) | -16.6 (65.6) | 0.1 | -8.8 | -7.6 (81.7) | -8.4 (89.7) |
| 50 | U[15, 30] | 0.1 | 36.8 | 20.6 | 12.9 | -12.6 (85.1) | -20.2 (41.1) | -0.4 | -5.9 | -7.0 (88.9) | -5.4 (95.1) |
| | | 0.3 | 35.8 | 25.4 | 15.7 | -17.1 (82.0) | -19.4 (46.2) | 0.1 | -7.6 | -6.9 (84.6) | -7.3 (93.1) |
| | | 0.7 | 34.3 | 30.1 | 15.6 | -21.5 (84.7) | -16.7 (61.7) | 0.6 | -13.2 | -10.5 (84.3) | -13.5 (90.9) |
| | U[25, 100] | 0.1 | 44.6 | 8.7 | 8.2 | 1.0 (87.0) | -3.2 (55.5) | 0.3 | -0.1 | -0.8 (83.5) | -0.1 (93.5) |
| | | 0.3 | 43.9 | 10.2 | 8.2 | 0.7 (80.4) | -13.5 (41.7) | -0.3 | -2.3 | -0.0 (77.3) | -2.2 (94.0) |
| | | 0.7 | 42.3 | 13.2 | 6.5 | -7.5 (88.4) | -25.9 (31.3) | 0.2 | -7.1 | -2.6 (81.8) | -7.0 (92.0) |

$NN_P$ and $BN_P$ are the estimates based on the population studies; $NN_O$ and $BN_O$ are the estimates based on the published studies; CN and CS are the Copas-N and Copas-Shi methods; $1SBN^{prop}$ are the proposed 1SBN model based sensitivity analysis methods; CP indicates the coverage probability.

# C ESTIMATIONS IN APPLICATION

## C.1 Example 1: rare-event meta-analysis of odds ratios

As mentioned in Section 6.1, we presented the data of example 1 in Table C12. The estimates of parameters were presented in Table C13-C16.

## C.2 Example 2: rare-event meta-analysis of odds ratios

As mentioned in Section 6.2, we presented the data of example 2 in Table C17. The estimates of parameters were shown in Table C18-C21.

## C.3 Example 3: rare-event meta-analysis of proportions

As mentioned in Section 6.3, the data of example 3 were shown in Table C12. The estimates of parameters were shown in Table C22-C25.

## C.4 Example 4: rare-event meta-analysis of proportions

As mentioned in Section 6.4, we presented the data of example 4 in Table C26. The estimates of parameters were shown in Table C27-C30.

## C.5 Computational time

As mentioned in Discussion of the main text, we summarized the computational time of the proposed methods and the t-statistic based method by setting 10 scenarios of sensitivity parameters. The computations were implemented by R (version 4.0.3) on system of Intel Xeon Platinum 8368. Parallel computations were conducted using R function `mclapply` by setting 10 cores.

**TABLE C12** Data of catheter-related bloodstream infection

| | Standard catheter | | AIT catheter | |
|---|---|---|---|---|
| Study | CRBSI ($y_{i0}$) | Patients ($n_{i0}$) | CRBSI ($y_{i1}$) | Patients ($n_{i1}$) |
| 1 | 3 | 117 | 0 | 116 |
| 2 | 3 | 35 | 1 | 44 |
| 3 | 9 | 195 | 2 | 208 |
| 4 | 7 | 136 | 0 | 130 |
| 5 | 6 | 157 | 5 | 151 |
| 6 | 4 | 139 | 1 | 98 |
| 7 | 3 | 177 | 1 | 174 |
| 8 | 2 | 39 | 1 | 74 |
| 9 | 19 | 103 | 1 | 97 |
| 10 | 2 | 122 | 1 | 113 |
| 11 | 7 | 64 | 0 | 66 |
| 12 | 1 | 58 | 0 | 70 |
| 13 | 5 | 175 | 3 | 188 |
| 14 | 11 | 180 | 6 | 187 |
| 15 | 0 | 105 | 0 | 118 |
| 16 | 1 | 262 | 0 | 252 |
| 17 | 3 | 362 | 1 | 345 |
| 18 | 1 | 69 | 4 | 64 |

**TABLE C13** Example 1: summary of the estimations of different sensitivity analysis methods

| | | The proposed HN model based method | | | The proposed CBN model based method | | |
|---|---|---|---|---|---|---|---|
| $(P_{\min}, P_{\max})$ | $M$ | $\theta$ (95% CI) | $\tau$ | $\rho$ | $\theta$ (95% CI) | $\tau$ | $\rho$ |
| (0.99, 0.999) | 0 | -1.352 (-2.047, -0.657) | 0.833 | -0.121 | -1.301 (-1.972, -0.631) | 0.775 | -0.119 |
| (0.90, 0.999) | 1 | -1.345 (-2.060, -0.629) | 0.834 | -0.154 | -1.295 (-1.985, -0.605) | 0.776 | -0.152 |
| (0.80, 0.999) | 1 | -1.337 (-2.073, -0.601) | 0.834 | -0.169 | -1.288 (-1.998, -0.578) | 0.776 | -0.168 |
| (0.70, 0.999) | 2 | -1.330 (-2.086, -0.573) | 0.835 | -0.179 | -1.281 (-2.010, -0.552) | 0.777 | -0.179 |
| (0.60, 0.999) | 3 | -1.321 (-2.098, -0.545) | 0.835 | -0.186 | -1.273 (-2.022, -0.525) | 0.777 | -0.186 |
| (0.50, 0.999) | 4 | -1.312 (-2.110, -0.515) | 0.835 | -0.190 | -1.265 (-2.034, -0.496) | 0.777 | -0.190 |
| (0.40, 0.999) | 6 | -1.302 (-2.123, -0.482) | 0.835 | -0.193 | -1.256 (-2.046, -0.465) | 0.777 | -0.193 |
| (0.30, 0.999) | 9 | -1.291 (-2.137, -0.446) | 0.835 | -0.194 | -1.245 (-2.060, -0.430) | 0.776 | -0.194 |
| (0.20, 0.999) | 14 | -1.277 (-2.152, -0.402) | 0.834 | -0.193 | -1.232 (-2.076, -0.388) | 0.776 | -0.193 |
| (0.10, 0.999) | 26 | -1.258 (-2.174, -0.342) | 0.832 | -0.187 | -1.214 (-2.097, -0.331) | 0.774 | -0.188 |

$M$ indicates the number of potentially unpublished studies; CI indicates the confidence interval.

**TABLE C14** Example 1: summary of the estimations of different sensitivity analysis methods

| | The Copas-N method (only0) | | | | The Copas-N method (all) | | | |
|---|---|---|---|---|---|---|---|---|
| $(P_{\min}, P_{\max})$ | $M_1$ | $\theta$ (95% CI) | $\tau$ | $\rho$ | $M_2$ | $\theta$ (95% CI) | $\tau$ | $\rho$ |
| (0.99, 0.999) | 0 | -1.177 (-1.669, -0.685) | 1.052 | -0.051 | 0 | -1.077 (-1.540, -0.615) | 0.987 | -0.077 |
| (0.90, 0.999) | 1 | -1.172 (-1.683, -0.662) | 1.052 | -0.071 | 1 | -1.071 (-1.554, -0.589) | 0.988 | -0.102 |
| (0.80, 0.999) | 2 | -1.168 (-1.697, -0.638) | 1.053 | -0.079 | 2 | -1.065 (-1.567, -0.563) | 0.988 | -0.112 |
| (0.70, 0.999) | 3 | -1.163 (-1.711, -0.614) | 1.053 | -0.084 | 3 | -1.059 (-1.580, -0.538) | 0.989 | -0.117 |
| (0.60, 0.999) | 4 | -1.157 (-1.725, -0.589) | 1.053 | -0.087 | 4 | -1.052 (-1.592, -0.512) | 0.989 | -0.120 |
| (0.50, 0.999) | 6 | -1.152 (-1.740, -0.563) | 1.053 | -0.089 | 5 | -1.045 (-1.604, -0.486) | 0.989 | -0.121 |
| (0.40, 0.999) | 9 | -1.145 (-1.755, -0.536) | 1.053 | -0.090 | 8 | -1.038 (-1.617, -0.459) | 0.989 | -0.121 |
| (0.30, 0.999) | 13 | -1.138 (-1.772, -0.505) | 1.053 | -0.090 | 11 | -1.029 (-1.630, -0.429) | 0.989 | -0.120 |
| (0.20, 0.999) | 21 | -1.130 (-1.790, -0.470) | 1.053 | -0.088 | 18 | -1.019 (-1.644, -0.395) | 0.989 | -0.117 |
| (0.10, 0.999) | 40 | -1.119 (-1.814, -0.423) | 1.053 | -0.085 | 35 | -1.006 (-1.663, -0.349) | 0.989 | -0.111 |

$M_1$ and $M_2$ indicate the number of potentially unpublished studies; only 0 indicates continuity correction for only studies with 0 cells; all indicates continuity correction for all the studies; CI indicates the confidence interval.

**TABLE C15** Example 1: summary of the estimations of different sensitivity analysis methods

| | The Copas-Shi method (only0) | | | | The Copas-Shi method (all) | | | |
|---|---|---|---|---|---|---|---|---|
| $(P_{\min}, P_{\max})$ | $M_1$ | $\theta$ (95% CI) | $\tau$ | $\rho$ | $M_2$ | $\theta$ (95% CI) | $\tau$ | $\rho$ |
| (0.99, 0.999) | 0 | -0.950 (-1.412, -0.488) | 0.001 | -0.369 | 0 | -0.857 (-1.280, -0.434) | 0.001 | -0.409 |
| (0.90, 0.999) | 1 | -0.918 (-1.389, -0.446) | 0.001 | -0.516 | 1 | -0.830 (-1.259, -0.401) | 0.001 | -0.565 |
| (0.80, 0.999) | 2 | -0.882 (-1.363, -0.400) | 0.001 | -0.574 | 2 | -0.801 (-1.236, -0.365) | 0.001 | -0.622 |
| (0.70, 0.999) | 3 | -0.845 (-1.338, -0.351) | 0.001 | -0.601 | 3 | -0.771 (-1.214, -0.328) | 0.001 | -0.646 |
| (0.60, 0.999) | 4 | -0.808 (-1.320, -0.296) | 0.001 | -0.603 | 4 | -0.741 (-1.194, -0.288) | 0.001 | -0.647 |
| (0.50, 0.999) | 6 | -0.776 (-1.311, -0.240) | 0.001 | -0.582 | 5 | -0.715 (-1.183, -0.247) | 0.001 | -0.623 |
| (0.40, 0.999) | 9 | -0.751 (-1.311, -0.191) | 0.001 | -0.540 | 8 | -0.695 (-1.182, -0.208) | 0.001 | -0.577 |
| (0.30, 0.999) | 13 | -0.734 (-1.311, -0.157) | 0.001 | -0.484 | 11 | -0.682 (-1.185, -0.179) | 0.001 | -0.513 |
| (0.20, 0.999) | 21 | -0.722 (-1.310, -0.134) | 0.001 | -0.420 | 18 | -0.674 (-1.187, -0.161) | 0.001 | -0.441 |
| (0.10, 0.999) | 40 | -0.711 (-1.308, -0.114) | 0.001 | -0.347 | 35 | -0.667 (-1.187, -0.148) | 0.001 | -0.360 |

$M_1$ and $M_2$ indicate the number of potentially unpublished studies; only 0 indicates continuity correction for only studies with 0 cells; all indicates continuity correction for all the studies; CI indicates the confidence interval.

**TABLE C16** Example 1: summary of the estimations of different sensitivity analysis methods

| | | The t-statistic and HN model based method (only0) | | | The t-statistic and HN model based method (all) | |
|---|---|---|---|---|---|---|
| $p$ | $M_1$ | $\theta$ (95% CI) | $\tau$ | $M_2$ | $\theta$ (95% CI) | $\tau$ |
| 1.00 | 0 | -1.353 (-2.041, -0.665) | 0.833 | 0 | -1.353 (-2.041, -0.665) | 0.833 |
| 0.90 | 2 | -1.204 (-1.939, -0.470) | 0.970 | 2 | -1.209 (-1.945, -0.473) | 0.963 |
| 0.80 | 4 | -1.033 (-1.897, -0.168) | 1.079 | 4 | -1.042 (-1.870, -0.215) | 1.070 |
| 0.70 | 8 | -0.842 (-1.891, 0.208) | 1.173 | 8 | -0.856 (-1.908, 0.195) | 1.162 |
| 0.60 | 12 | -0.631 (-1.996, 0.734) | 1.255 | 12 | -0.652 (-1.980, 0.677) | 1.243 |
| 0.50 | 18 | -0.404 (-2.227, 1.419) | 1.323 | 18 | -0.429 (-2.154, 1.296) | 1.312 |
| 0.40 | 27 | -0.171 (-2.690, 2.349) | 1.370 | 27 | -0.192 (-2.461, 2.077) | 1.365 |
| 0.30 | 42 | 0.027 (-3.554, 3.608) | 1.377 | 42 | 0.043 (-2.952, 3.039) | 1.394 |
| 0.20 | 72 | 0.093 (-4.498, 4.684) | 1.313 | 72 | 0.238 (-3.658, 4.134) | 1.382 |
| 0.10 | 162 | 0.017 (-4.927, 4.961) | 1.207 | 162 | 0.302 (-4.339, 4.944) | 1.307 |

*M* indicates the number of potentially unpublished studies; only 0 indicates continuity correction for only studies with 0 cells; all indicates continuity correction for all the studies; CI indicates the confidence interval.

**TABLE C17** Data of catheter-related bloodstream infection

| | Magnesium group | | Control group | |
|---|---|---|---|---|
| Study | Deaths ($y_{i0}$) | Patients ($n_{i0}$) | Deaths ($y_{i1}$) | Patients ($n_{i1}$) |
| 1 | 1 | 40 | 2 | 36 |
| 2 | 9 | 135 | 23 | 135 |
| 3 | 2 | 200 | 7 | 200 |
| 4 | 1 | 48 | 1 | 46 |
| 5 | 10 | 150 | 8 | 148 |
| 6 | 1 | 59 | 9 | 56 |
| 7 | 1 | 25 | 3 | 23 |
| 8 | 0 | 22 | 1 | 21 |
| 9 | 6 | 76 | 11 | 75 |
| 10 | 1 | 27 | 7 | 27 |
| 11 | 2 | 89 | 12 | 80 |
| 12 | 5 | 23 | 13 | 33 |
| 13 | 4 | 130 | 8 | 122 |
| 14 | 90 | 1159 | 118 | 1157 |
| 15 | 4 | 107 | 17 | 108 |
| 16 | 2216 | 29011 | 2103 | 29039 |

**TABLE C18** Example 2: summary of the estimations of different sensitivity analysis methods

| | | The proposed HN model based method | | | The proposed CBN model based method | | |
|---|---|---|---|---|---|---|---|
| $(P_{\min}, P_{\max})$ | $M$ | $\theta$ (95% CI) | $\tau$ | $\rho$ | $\theta$ (95% CI) | $\tau$ | $\rho$ |
| (0.99, 0.999) | 0 | -0.841 (-1.295, -0.388) | 0.569 | -0.999 | -0.750 (-1.174, -0.325) | 0.510 | -0.990 |
| (0.90, 0.999) | 1 | -0.796 (-1.227, -0.364) | 0.592 | -0.999 | -0.711 (-1.114, -0.308) | 0.530 | -0.990 |
| (0.80, 0.999) | 3 | -0.733 (-1.132, -0.334) | 0.608 | -0.999 | -0.654 (-1.025, -0.283) | 0.547 | -0.990 |
| (0.70, 0.999) | 6 | -0.658 (-1.022, -0.294) | 0.625 | -0.999 | -0.586 (-0.926, -0.247) | 0.561 | -0.990 |
| (0.60, 0.999) | 9 | -0.573 (-0.909, -0.237) | 0.641 | -0.999 | -0.511 (-0.827, -0.194) | 0.574 | -0.990 |
| (0.50, 0.999) | 13 | -0.477 (-0.799, -0.154) | 0.656 | -0.999 | -0.427 (-0.735, -0.120) | 0.584 | -0.990 |
| (0.40, 0.999) | 19 | -0.368 (-0.700, -0.037) | 0.667 | -0.999 | -0.334 (-0.655, -0.014) | 0.590 | -0.990 |
| (0.30, 0.999) | 30 | -0.243 (-0.615, 0.130) | 0.674 | -0.999 | -0.228 (-0.590, 0.133) | 0.592 | -0.990 |
| (0.20, 0.999) | 50 | -0.089 (-0.545, 0.366) | 0.675 | -0.999 | -0.101 (-0.541, 0.338) | 0.587 | -0.990 |
| (0.10, 0.999) | 107 | 0.118 (-0.490, 0.726) | 0.662 | -0.999 | 0.065 (-0.511, 0.640) | 0.566 | -0.990 |

*M* indicates the number of potentially unpublished studies; CI indicates the confidence interval.

**TABLE C19** Example 2: summary of the estimations of different sensitivity analysis methods

| | The Copas-N method (only0) | | | | The Copas-N method (all) | | | |
|---|---|---|---|---|---|---|---|---|
| $(P_{\min}, P_{\max})$ | $M_1$ | $\theta$ (95% CI) | $\tau$ | $\rho$ | $M_2$ | $\theta$ (95% CI) | $\tau$ | $\rho$ |
| (0.99, 0.999) | 0 | -1.013 (-1.387, -0.640) | 0.756 | -0.990 | 0 | -0.905 (-1.232, -0.577) | 0.661 | -0.990 |
| (0.90, 0.999) | 1 | -0.870 (-1.190, -0.551) | 0.865 | -0.990 | 1 | -0.768 (-1.052, -0.484) | 0.768 | -0.990 |
| (0.80, 0.999) | 3 | -0.808 (-1.206, -0.410) | 0.871 | -0.871 | 3 | -0.731 (-1.089, -0.373) | 0.755 | -0.843 |
| (0.70, 0.999) | 6 | -0.704 (-1.130, -0.277) | 0.905 | -0.863 | 6 | -0.645 (-1.042, -0.249) | 0.781 | -0.834 |
| (0.60, 0.999) | 9 | -0.589 (-1.081, -0.098) | 0.935 | -0.860 | 9 | -0.555 (-1.023, -0.086) | 0.802 | -0.828 |
| (0.50, 0.999) | 13 | -0.466 (-1.072, 0.140) | 0.959 | -0.856 | 13 | -0.460 (-1.034, 0.114) | 0.817 | -0.819 |
| (0.40, 0.999) | 19 | -0.337 (-1.123, 0.449) | 0.975 | -0.846 | 19 | -0.362 (-1.068, 0.345) | 0.825 | -0.806 |
| (0.30, 0.999) | 29 | -0.210 (-1.238, 0.817) | 0.977 | -0.825 | 29 | -0.261 (-1.114, 0.592) | 0.826 | -0.787 |
| (0.20, 0.999) | 49 | -0.093 (-1.359, 1.172) | 0.959 | -0.790 | 48 | -0.154 (-1.162, 0.855) | 0.816 | -0.760 |
| (0.10, 0.999) | 107 | 0.018 (-1.427, 1.463) | 0.918 | -0.732 | 104 | -0.037 (-1.208, 1.135) | 0.791 | -0.715 |

$M_1$ and $M_2$ indicate the number of potentially unpublished studies; only 0 indicates continuity correction for only studies with 0 cells; all indicates continuity correction for all the studies; CI indicates the confidence interval.

**TABLE C20** Example 2: summary of the estimations of different sensitivity analysis methods

| | The Copas-Shi method (only0) | | | | The Copas-Shi method (all) | | | |
|---|---|---|---|---|---|---|---|---|
| $(P_{\min}, P_{\max})$ | $M_1$ | $\theta$ (95% CI) | $\tau$ | $\rho$ | $M_2$ | $\theta$ (95% CI) | $\tau$ | $\rho$ |
| (0.99, 0.999) | 0 | -0.737 (-1.187, -0.288) | 0.500 | -0.561 | 0 | -0.683 (-1.092, -0.273) | 0.459 | -0.541 |
| (0.90, 0.999) | 1 | -0.647 (-1.103, -0.190) | 0.459 | -0.828 | 1 | -0.603 (-1.019, -0.186) | 0.424 | -0.817 |
| (0.80, 0.999) | 3 | -0.534 (-0.977, -0.090) | 0.409 | -0.931 | 3 | -0.499 (-0.905, -0.092) | 0.378 | -0.928 |
| (0.70, 0.999) | 6 | -0.405 (-0.859, 0.050) | 0.356 | -0.990 | 6 | -0.377 (-0.796, 0.041) | 0.328 | -0.990 |
| (0.60, 0.999) | 9 | -0.256 (-0.716, 0.205) | 0.281 | -0.990 | 9 | -0.238 (-0.656, 0.180) | 0.259 | -0.990 |
| (0.50, 0.999) | 13 | -0.132 (-0.476, 0.213) | 0.191 | -0.957 | 13 | -0.121 (-0.440, 0.197) | 0.182 | -0.962 |
| (0.40, 0.999) | 19 | 0.045 (-0.015, 0.106) | 0.001 | -0.936 | 19 | 0.046 (-0.015, 0.106) | 0.001 | -0.939 |
| (0.30, 0.999) | 29 | 0.046 (-0.016, 0.108) | 0.001 | -0.875 | 29 | 0.046 (-0.016, 0.108) | 0.001 | -0.876 |
| (0.20, 0.999) | 49 | 0.046 (-0.015, 0.108) | 0.001 | -0.796 | 48 | 0.046 (-0.015, 0.108) | 0.001 | -0.796 |
| (0.10, 0.999) | 107 | 0.046 (-0.015, 0.108) | 0.001 | -0.697 | 104 | 0.046 (-0.015, 0.108) | 0.001 | -0.695 |

$M_1$ and $M_2$ indicate the number of potentially unpublished studies; only 0 indicates continuity correction for only studies with 0 cells; all indicates continuity correction for all the studies; CI indicates the confidence interval.

**TABLE C21** Example 2: summary of the estimations of different sensitivity analysis methods

| | The t-statistic and HN model based method (only0) | | | The t-statistic and HN model based method (all) | | |
|---|---|---|---|---|---|---|
| $p$ | $M_1$ | $\theta$ (95% CI) | $\tau$ | $M_2$ | $\theta$ (95% CI) | $\tau$ |
| 1.00 | 0 | -0.951 (-1.392, -0.510) | 0.457 | 0 | -0.951 (-1.392, -0.510) | 0.457 |
| 0.90 | 2 | -0.945 (-1.388, -0.502) | 0.463 | 2 | -0.945 (-1.394, -0.496) | 0.462 |
| 0.80 | 4 | -0.917 (-1.223, -0.611) | 0.548 | 4 | -0.918 (-1.254, -0.582) | 0.537 |
| 0.70 | 7 | -1.041 (-1.465, -0.616) | 0.535 | 7 | -1.033 (-1.440, -0.626) | 0.521 |
| 0.60 | 11 | -1.127 (NaN, NaN) | 0.510 | 11 | -1.113 (NaN, NaN) | 0.495 |
| 0.50 | 16 | -1.185 (NaN, NaN) | 0.479 | 16 | -1.168 (NaN, NaN) | 0.464 |
| 0.40 | 24 | -1.224 (-2.633, 0.186) | 0.444 | 24 | -1.204 (-1.603, -0.806) | 0.430 |
| 0.30 | 37 | -1.249 (-1.474, -1.024) | 0.408 | 37 | -1.229 (-1.424, -1.034) | 0.395 |
| 0.20 | 64 | -1.265 (-1.424, -1.106) | 0.368 | 64 | -1.246 (-1.394, -1.098) | 0.357 |
| 0.10 | 144 | -1.276 (-1.416, -1.136) | 0.319 | 144 | -1.259 (-1.394, -1.124) | 0.311 |

$M$ indicates the number of potentially unpublished studies; only 0 indicates continuity correction for only studies with 0 cells; all indicates continuity correction for all the studies; CI indicates the confidence interval.

**TABLE C22** Example 3: summary of the estimations of different sensitivity analysis methods

| | | The proposed 1SBN model based method | | |
|---|---|---|---|---|
| $(P_{\min}, P_{\max})$ | $M$ | $\theta$ (95% CI) | $\tau$ | $\rho$ |
| (0.99, 0.999) | 0 | -4.818 (-5.515, -4.122) | 0.912 | 0.990 |
| (0.90, 0.999) | 1 | -4.850 (-5.554, -4.146) | 0.929 | 0.990 |
| (0.80, 0.999) | 1 | -4.885 (-5.599, -4.170) | 0.945 | 0.990 |
| (0.70, 0.999) | 2 | -4.923 (-5.650, -4.195) | 0.960 | 0.990 |
| (0.60, 0.999) | 3 | -4.965 (-5.709, -4.221) | 0.974 | 0.990 |
| (0.50, 0.999) | 4 | -5.013 (-5.779, -4.247) | 0.986 | 0.990 |
| (0.40, 0.999) | 6 | -5.069 (-5.863, -4.275) | 0.996 | 0.990 |
| (0.30, 0.999) | 9 | -5.136 (-5.968, -4.304) | 1.001 | 0.990 |
| (0.20, 0.999) | 14 | -5.096 (-6.206, -3.987) | 0.950 | 0.729 |
| (0.10, 0.999) | 27 | -5.088 (-6.124, -4.051) | 0.921 | 0.558 |

$M$ indicates the number of potentially unpublished studies; CI indicates the confidence interval.

**TABLE C23** Example 3: summary of the estimations of different sensitivity analysis methods

| | The Copas-N method (only0) | | | | The Copas-N method (all) | | | |
|---|---|---|---|---|---|---|---|---|
| $(P_{\min}, P_{\max})$ | $M_1$ | $\theta$ (95% CI) | $\tau$ | $\rho$ | $M_2$ | $\theta$ (95% CI) | $\tau$ | $\rho$ |
| (0.99, 0.999) | 0 | -4.652 (-5.069, -4.235) | 0.896 | 0.985 | 0 | -4.460 (-4.894, -4.026) | 0.934 | 0.990 |
| (0.90, 0.999) | 1 | -4.652 (-5.069, -4.235) | 0.896 | 0.990 | 1 | -4.460 (-4.894, -4.026) | 0.934 | 0.990 |
| (0.80, 0.999) | 2 | -4.652 (-5.069, -4.235) | 0.896 | 0.990 | 2 | -4.460 (-4.894, -4.027) | 0.934 | 0.990 |
| (0.70, 0.999) | 4 | -4.652 (-5.067, -4.238) | 0.896 | 0.990 | 3 | -4.474 (-4.863, -4.085) | 0.940 | 0.990 |
| (0.60, 0.999) | 6 | -4.671 (-5.029, -4.313) | 0.901 | 0.990 | 5 | -4.549 (-4.857, -4.241) | 0.965 | 0.990 |
| (0.50, 0.999) | 9 | -4.751 (-5.028, -4.474) | 0.918 | 0.990 | 8 | -4.670 (-4.932, -4.407) | 0.995 | 0.990 |
| (0.40, 0.999) | 13 | -4.876 (-5.111, -4.641) | 0.938 | 0.990 | 11 | -4.819 (-5.058, -4.581) | 1.028 | 0.990 |
| (0.30, 0.999) | 20 | -5.036 (-5.255, -4.816) | 0.960 | 0.990 | 17 | -5.004 (-5.241, -4.768) | 1.066 | 0.990 |
| (0.20, 0.999) | 34 | -5.245 (-5.481, -5.010) | 0.987 | 0.990 | 29 | -5.054 (-5.417, -4.691) | 1.104 | 0.927 |
| (0.10, 0.999) | 71 | -5.424 (-5.743, -5.105) | 1.017 | 0.957 | 62 | -5.021 (-5.781, -4.262) | 1.022 | 0.775 |

$M_1$ and $M_2$ indicate the number of potentially unpublished studies; only 0 indicates continuity correction for only studies with 0 cells; all indicates continuity correction for all the studies; CI indicates the confidence interval.

**TABLE C24** Example 3: summary of the estimations of different sensitivity analysis methods

| | The Copas-Shi method (only0) | | | | The Copas-Shi method (all) | | | |
|---|---|---|---|---|---|---|---|---|
| $(P_{\min}, P_{\max})$ | $M_1$ | $\theta$ (95% CI) | $\tau$ | $\rho$ | $M_2$ | $\theta$ (95% CI) | $\tau$ | $\rho$ |
| (0.99, 0.999) | 0 | -4.229 (-4.786, -3.672) | 0.576 | -0.481 | 0 | -4.023 (-4.516, -3.531) | 0.510 | -0.556 |
| (0.90, 0.999) | 1 | -4.156 (-4.714, -3.598) | 0.533 | -0.713 | 1 | -3.965 (-4.459, -3.471) | 0.473 | -0.762 |
| (0.80, 0.999) | 2 | -4.072 (-4.624, -3.520) | 0.481 | -0.813 | 2 | -3.906 (-4.392, -3.419) | 0.435 | -0.837 |
| (0.70, 0.999) | 4 | -3.981 (-4.524, -3.438) | 0.419 | -0.879 | 3 | -3.845 (-4.322, -3.368) | 0.393 | -0.889 |
| (0.60, 0.999) | 6 | -3.877 (-4.415, -3.340) | 0.337 | -0.930 | 5 | -3.778 (-4.246, -3.310) | 0.344 | -0.933 |
| (0.50, 0.999) | 9 | -3.723 (-4.055, -3.390) | 0.001 | -0.977 | 8 | -3.636 (-3.907, -3.365) | 0.001 | -0.990 |
| (0.40, 0.999) | 13 | -3.684 (-4.035, -3.333) | 0.001 | -0.990 | 11 | -3.601 (-3.877, -3.326) | 0.001 | -0.990 |
| (0.30, 0.999) | 20 | -3.454 (-3.781, -3.126) | 0.001 | -0.990 | 17 | -3.442 (-3.757, -3.126) | 0.001 | -0.990 |
| (0.20, 0.999) | 34 | -3.483 (-3.913, -3.053) | 0.001 | -0.829 | 29 | -3.472 (-3.847, -3.097) | 0.001 | -0.817 |
| (0.10, 0.999) | 71 | -3.479 (-3.918, -3.040) | 0.001 | -0.701 | 62 | -3.461 (-3.845, -3.077) | 0.001 | -0.699 |

$M_1$ and $M_2$ indicate the number of potentially unpublished studies; only 0 indicates continuity correction for only studies with 0 cells; all indicates continuity correction for all the studies; CI indicates the confidence interval.

**TABLE C25** Example 3: summary of the estimations of different sensitivity analysis methods

| | | The t-statistic and HN model based method (only0) | | The t-statistic and HN model based method (all) | |
|---|---|---|---|---|---|
| $p$ | $M$ | $\theta$ (95% CI) | $\tau$ | $\theta$ (95% CI) | $\tau$ |
| 1.00 | 0 | -4.812 (-5.508, -4.116) | 0.908 | -4.812 (-5.508, -4.116) | 0.908 |
| 0.90 | 2 | -4.672 (-5.078, -4.266) | 0.929 | -4.557 (-4.787, -4.326) | 0.961 |
| 0.80 | 4 | -4.418 (-5.149, -3.687) | 0.863 | -4.513 (-5.124, -3.903) | 0.889 |
| 0.70 | 8 | -4.431 (-4.996, -3.866) | 0.834 | -4.438 (-5.068, -3.807) | 0.859 |
| 0.60 | 12 | -4.357 (-4.927, -3.786) | 0.798 | -4.369 (-5.032, -3.705) | 0.833 |
| 0.50 | 18 | -4.285 (-4.869, -3.701) | 0.764 | -4.304 (-5.004, -3.603) | 0.808 |
| 0.40 | 27 | -4.214 (-4.820, -3.608) | 0.729 | -4.240 (-4.980, -3.500) | 0.783 |
| 0.30 | 42 | -4.139 (-4.767, -3.512) | 0.691 | -4.173 (-4.953, -3.392) | 0.756 |
| 0.20 | 72 | -4.055 (-4.706, -3.404) | 0.646 | -4.097 (-4.926, -3.268) | 0.724 |
| 0.10 | 162 | -3.943 (-4.618, -3.269) | 0.582 | -3.995 (-4.876, -3.114) | 0.678 |

$M$ indicates the number of potentially unpublished studies; only 0 indicates continuity correction for only studies with 0 cells; all indicates continuity correction for all the studies; CI indicates the confidence interval.

**TABLE C26** Data of Example 4

| Study | Improved ($y_{i0}$) | Patients ($n_{i1}$) | Not improved ($y_{i1}$) |
|---|---|---|---|
| 1 | 16 | 17 | 1 |
| 2 | 10 | 12 | 2 |
| 3 | 4 | 8 | 4 |
| 4 | 43 | 58 | 15 |
| 5 | 10 | 10 | 0 |
| 6 | 25 | 42 | 17 |
| 7 | 13 | 14 | 1 |
| 8 | 12 | 12 | 0 |
| 9 | 22 | 41 | 19 |
| 10 | 4 | 5 | 1 |
| 11 | 5 | 6 | 1 |
| 12 | 18 | 23 | 5 |
| 13 | 58 | 68 | 10 |
| 14 | 6 | 10 | 4 |

**TABLE C27** Example 4: summary of the estimations of different sensitivity analysis methods

| | | The proposed 1SBN model based method | | |
|---|---|---|---|---|
| $(P_{\min}, P_{\max})$ | $M$ | $\theta$ (95% CI) | $\tau$ | $\rho$ |
| (0.99, 0.999) | 0 | -1.374 (-1.941, -0.808) | 0.774 | -0.990 |
| (0.90, 0.999) | 1 | -1.338 (-1.893, -0.782) | 0.805 | -0.990 |
| (0.80, 0.999) | 1 | -1.291 (-1.826, -0.755) | 0.813 | -0.990 |
| (0.70, 0.999) | 2 | -1.265 (-1.830, -0.700) | 0.805 | -0.871 |
| (0.60, 0.999) | 3 | -1.245 (-1.831, -0.659) | 0.798 | -0.776 |
| (0.50, 0.999) | 5 | -1.224 (-1.827, -0.621) | 0.790 | -0.709 |
| (0.40, 0.999) | 7 | -1.203 (-1.823, -0.583) | 0.783 | -0.658 |
| (0.30, 0.999) | 10 | -1.180 (-1.817, -0.542) | 0.776 | -0.614 |
| (0.20, 0.999) | 16 | -1.153 (-1.811, -0.495) | 0.767 | -0.570 |
| (0.10, 0.999) | 32 | -1.119 (-1.803, -0.434) | 0.754 | -0.516 |

$M$ indicates the number of potentially unpublished studies; CI indicates the confidence interval.

**TABLE C28** Example 4: summary of the estimations of different sensitivity analysis methods

| | The Copas-N method (only0) | | | | The Copas-N method (all) | | | |
|---|---|---|---|---|---|---|---|---|
| $(P_{\min}, P_{\max})$ | $M_1$ | $\theta$ (95% CI) | $\tau$ | $\rho$ | $M_2$ | $\theta$ (95% CI) | $\tau$ | $\rho$ |
| (0.99, 0.999) | 0 | -1.512 (-2.055, -0.969) | 0.998 | -0.990 | 0 | -1.393 (-1.911, -0.876) | 0.947 | -0.990 |
| (0.90, 0.999) | 1 | -1.424 (-1.885, -0.964) | 1.059 | -0.990 | 1 | -1.326 (-1.767, -0.885) | 0.994 | -0.990 |
| (0.80, 0.999) | 1 | -1.361 (-2.087, -0.635) | 1.081 | -0.836 | 1 | -1.146 (-1.562, -0.729) | 1.110 | -0.990 |
| (0.70, 0.999) | 2 | -1.406 (-2.100, -0.711) | 1.026 | -0.483 | 2 | -1.315 (-2.017, -0.612) | 0.963 | -0.375 |
| (0.60, 0.999) | 4 | -1.392 (-2.083, -0.702) | 1.021 | -0.417 | 3 | -1.307 (-1.993, -0.621) | 0.960 | -0.316 |
| (0.50, 0.999) | 5 | -1.376 (-2.080, -0.672) | 1.018 | -0.379 | 5 | -1.295 (-1.990, -0.601) | 0.958 | -0.287 |
| (0.40, 0.999) | 8 | -1.358 (-2.080, -0.636) | 1.015 | -0.351 | 7 | -1.282 (-1.991, -0.573) | 0.956 | -0.266 |
| (0.30, 0.999) | 12 | -1.338 (-2.081, -0.594) | 1.011 | -0.326 | 10 | -1.267 (-1.993, -0.541) | 0.955 | -0.249 |
| (0.20, 0.999) | 19 | -1.314 (-2.082, -0.546) | 1.007 | -0.302 | 15 | -1.249 (-1.995, -0.502) | 0.952 | -0.232 |
| (0.10, 0.999) | 39 | -1.283 (-2.082, -0.484) | 1.002 | -0.273 | 31 | -1.224 (-1.997, -0.451) | 0.949 | -0.212 |

$M_1$ and $M_2$ indicate the number of potentially unpublished studies; only 0 indicates continuity correction for only studies with 0 cells; all indicates continuity correction for all the studies; CI indicates the confidence interval.

**TABLE C29** Example 4: summary of the estimations of different sensitivity analysis methods

| | The Copas-Shi method (only0) | | | | The Copas-Shi method (all) | | | |
|---|---|---|---|---|---|---|---|---|
| $(P_{\min}, P_{\max})$ | $M_1$ | $\theta$ (95% CI) | $\tau$ | $\rho$ | $M_2$ | $\theta$ (95% CI) | $\tau$ | $\rho$ |
| (0.99, 0.999) | 0 | -1.102 (-1.577, -0.626) | 0.535 | -0.990 | 0 | -1.042 (-1.474, -0.611) | 0.495 | -0.990 |
| (0.90, 0.999) | 1 | -1.037 (-1.492, -0.583) | 0.495 | -0.990 | 1 | -0.994 (-1.413, -0.576) | 0.466 | -0.990 |
| (0.80, 0.999) | 1 | -0.992 (-1.434, -0.549) | 0.475 | -0.990 | 1 | -0.958 (-1.369, -0.548) | 0.451 | -0.990 |
| (0.70, 0.999) | 2 | -0.953 (-1.386, -0.519) | 0.465 | -0.990 | 2 | -0.926 (-1.330, -0.522) | 0.442 | -0.990 |
| (0.60, 0.999) | 4 | -0.916 (-1.339, -0.492) | 0.459 | -0.985 | 3 | -0.893 (-1.289, -0.497) | 0.436 | -0.990 |
| (0.50, 0.999) | 5 | -0.869 (-1.278, -0.459) | 0.451 | -0.990 | 5 | -0.855 (-1.241, -0.468) | 0.430 | -0.990 |
| (0.40, 0.999) | 8 | -0.804 (-1.194, -0.414) | 0.441 | -0.990 | 7 | -0.803 (-1.176, -0.431) | 0.423 | -0.990 |
| (0.30, 0.999) | 12 | -0.693 (-1.060, -0.326) | 0.435 | -0.990 | 10 | -0.718 (-1.068, -0.368) | 0.410 | -0.990 |
| (0.20, 0.999) | 19 | -0.736 (-1.209, -0.264) | 0.439 | -0.810 | 15 | -0.729 (-1.176, -0.282) | 0.423 | -0.829 |
| (0.10, 0.999) | 39 | -0.742 (-1.212, -0.273) | 0.439 | -0.671 | 31 | -0.733 (-1.177, -0.289) | 0.422 | -0.685 |

$M_1$ and $M_2$ indicate the number of potentially unpublished studies; only 0 indicates continuity correction for only studies with 0 cells; all indicates continuity correction for all the studies; CI indicates the confidence interval.

**TABLE C30** Example 4: summary of the estimations of different sensitivity analysis methods

| | | The t-statistic and HN model based method (only0) | | The t-statistic and HN model based method (all) | |
|---|---|---|---|---|---|
| $p$ | $M$ | $\theta$ (95% CI) | $\tau$ | $\theta$ (95% CI) | $\tau$ |
| 1.00 | 0 | -1.377 (-1.942, -0.811) | 0.768 | -1.377 (-1.942, -0.811) | 0.768 |
| 0.90 | 2 | -1.446 (-2.013, -0.879) | 0.776 | -1.439 (-2.002, -0.876) | 0.770 |
| 0.80 | 4 | -1.500 (-2.079, -0.922) | 0.771 | -1.490 (-2.058, -0.922) | 0.763 |
| 0.70 | 6 | -0.721 (-1.110, -0.332) | 1.417 | -0.721 (-1.109, -0.332) | 1.418 |
| 0.60 | 9 | -0.374 (-0.693, -0.055) | 1.631 | -0.373 (-0.693, -0.054) | 1.632 |
| 0.50 | 14 | 0.052 (-0.280, 0.383) | 1.853 | 0.052 (-0.279, 0.383) | 1.853 |
| 0.40 | 21 | -1.670 (-2.302, -1.038) | 0.718 | -1.651 (-2.261, -1.041) | 0.707 |
| 0.30 | 33 | -1.710 (-2.360, -1.060) | 0.700 | -1.689 (-2.314, -1.065) | 0.687 |
| 0.20 | 56 | -1.752 (-2.424, -1.081) | 0.677 | -1.731 (-2.372, -1.089) | 0.664 |
| 0.10 | 126 | -1.803 (-2.507, -1.100) | 0.648 | -1.781 (-2.448, -1.113) | 0.633 |

$M$ indicates the number of potentially unpublished studies; only 0 indicates continuity correction for only studies with 0 cells; all indicates continuity correction for all the studies; CI indicates the confidence interval.

**TABLE C31** Computational time (seconds) of the proposed methods and $t$-statistic based method

| | Example 1 | Example 2 | Example 3 | Example 4 |
|---|---|---|---|---|
| Proposed methods (both the HN and BN models based) | 17 | 34 | 4 | 3 |
| The t-statistic based method (only the HN model based) | 97 | 3071 | 419 | 84 |

# References


1. Copas J. What Works?: Selectivity Models and Meta-Analysis. *J. R. Stat. Soc. Ser. A Stat. Soc.* 1999;162(1):95–109.

2. Hu T, Zhou Y, Hattori S. Sensitivity analysis for publication bias in meta-analysis of sparse data based on exact likelihood. *Biometrics.* 2024;80(3):1–17.

3. Fog A. Calculation Methods for Wallenius' Noncentral Hypergeometric Distribution. *Commun. Stat. - Simul. Comput.*. 2008;37(2):258–273.

4. Fog A. Sampling Methods for Wallenius' and Fisher's Noncentral Hypergeometric Distributions. *Commun. Stat. - Simul. Comput.*. 2008;37(2):241–257.

5. Narasimhan B, Johnson SG, Hahn T, Bouvier A, Kiêu K. *cubature: Adaptive Multivariate Integration over Hypercubes*. 2025. R package version 2.1.4.
